# Advancing conflict research and response through satellite-derived data

**DOI:** 10.1038/s41586-026-11004-6

**Published:** 2026-09-09

**Authors:** Valerie Sticher, Corinne Bara, Jenniina Kotajoki, Lars Bromley, Thierry Crevoisier, Emanuele Dalsasso, Olivier Dietrich, Karsten Donnay, Filip Dorm, Manuel Fiol, Xi Li, Mikael Hiberg Naghizadeh, Corey Scher, Jamon Van Den Hoek, Konrad Schindler, Devis Tuia, Jan Dirk Wegner

**Affiliations:** 1 Center for Security Studies, ETH Zurich, Zurich, Switzerland; 2 Department of Political Science, University of Zurich, Zurich, Switzerland; 3 United Nations Satellite Centre (UNOSAT), Geneva, Switzerland; 4 World Food Programme (WFP), Rome, Italy; 5 Environmental Computational Science and Earth Observation Laboratory, EPFL, Sion, Switzerland; 6 Université Grenoble Alpes, Inria, CNRS, Grenoble INP, LJK, Grenoble, France; 7 Photogrammetry and Remote Sensing, ETH Zurich, Zurich, Switzerland; 8 Digital Society Initiative, University of Zurich, Zurich, Switzerland; 9 State Key Laboratory of Information Engineering in Surveying, Mapping and Remote Sensing, Wuhan University, Wuhan, China; 10 German Institute of Global and Area Studies, Hamburg, Germany; 11 College of Earth, Ocean, and Atmospheric Sciences, Oregon State University, Corvallis, OR USA; 12 EcoVision Lab, Department of Mathematical Modeling and Machine Learning, University of Zurich, Zurich, Switzerland

**Keywords:** Politics, Interdisciplinary studies, Environmental social sciences, Computer science

## Abstract

With armed conflicts at a historic high and attacks on civilians rising^1^, understanding the evolving nature of conflict is a critical research priority. The current paradigm in conflict research relies heavily on text-based data, using fatalities as the primary—and often sole—proxy for violence intensity. Although these data have expanded our ability to study armed conflict, they exhibit inherent limitations due to uneven human reporting^2–6^. War damage assessments based on satellite data^7–13^ offer a complementary perspective. Satellite-derived data have their own limitations, but these arise from different mechanisms, creating distinct, complementary strengths that can be leveraged through data integration. Here we propose three concrete approaches to integration: improvement, enrichment and fusion. Each bridges a different gap in the underlying data sources. We use case studies from Ukraine and Myanmar to illustrate how integration can be implemented in practice and the novel analytical insights that emerge. Prioritizing data integration enables a paradigm shift away from fatality-centric research towards a broader spectrum of violence, revealing the complexity of conflict dynamics.

## Abstract (web summary)

Integrating satellite-derived war-damage data with text-based fatality records through improvement, enrichment and fusion mitigates limitations inherent in each source, revealing complex violence dynamics beyond fatality-centric paradigms, as case studies from Ukraine and Myanmar illustrate.

## Main

We are witnessing a record number of armed conflicts, and attacks on civilians are increasing in number^1^. In conflicts such as those in Gaza, Ukraine, Sudan and Myanmar, the use of heavy weaponry, drone warfare and arson has caused widespread death, destruction and displacement, leaving lasting scars on entire countries. This makes understanding the evolving nature of armed conflicts and developing strategies to reduce violence a critical research priority.

Despite the urgency of studying armed conflict, there are important limitations in the available data. Over the past 15 years, conflict research has made substantial progress with the emergence of detailed conflict event datasets based primarily on media sources^14–16^. However, these datasets exhibit well-documented limitations and biases that reflect the uneven availability of human reporting^2–6^. Most notably, because media reporting is attention-driven, there is a lack of reliable data from places and periods that receive little media coverage, and from types of event that often go unreported or are reported only in unspecific terms^17,18^, such as the destruction of dwellings and crops or the displacement of populations. Accordingly, quantitative studies of conflict-related violence focus predominantly on fatalities and often leave other aspects of violence and their social and environmental effects unaddressed.

We propose an approach to broaden this focus through the integration of text-based conflict event data with satellite-derived data. Here we use the term satellite-derived data to refer to information generated through the analysis of satellite data (see Supplementary Information, section B). Advances in automated satellite analysis^7–10,19–22^ and increasingly available open-access satellite data make this a pivotal moment for such data integration. Prior work has highlighted the potential of satellite data for humanitarian action^23–25^ and provided remote-sensing-based perspectives on specific conflicts^9,10,26–29^. In this Analysis, we demonstrate how the fundamentally different data generation processes of text-based and satellite-derived data lead to unique strengths and limitations that can be overcome only through data integration. We propose three approaches to integration—improvement, enrichment and fusion—and use empirical examples from Ukraine and Myanmar to illustrate their feasibility and the novel empirical insights that they provide. We advocate for prioritizing this integration to enable a paradigm shift away from fatality-centric research towards a broader spectrum of violence, to reveal the complex, interactive dynamics that shape conflict landscapes.

## Expanding our view on violence

To reduce impact on civilians and the long-term consequences of war, we must understand when and where conflict actors use violence to advance their military or political aims. This makes data on conflict-related violence central to many research efforts. Although approaches such as fieldwork and surveys offer invaluable insights into various conflict dynamics, large-scale analyses across time and space require techniques for systematic, retrospective data collection. The current paradigm in conflict research relies almost exclusively on data derived from human reporting. Yet uneven reporting means that we currently focus primarily on a relatively narrow, although highly important, spectrum of conflict-related violence. Emerging approaches that automatically analyse satellite data offer a complementary perspective and promise to expand both the scope and depth of conflict research. To realize this potential, we must understand what each data source captures, how the data are generated, and how the generation process shapes what we observe and with what precision.

### Text-based data

**Fig. 1:**
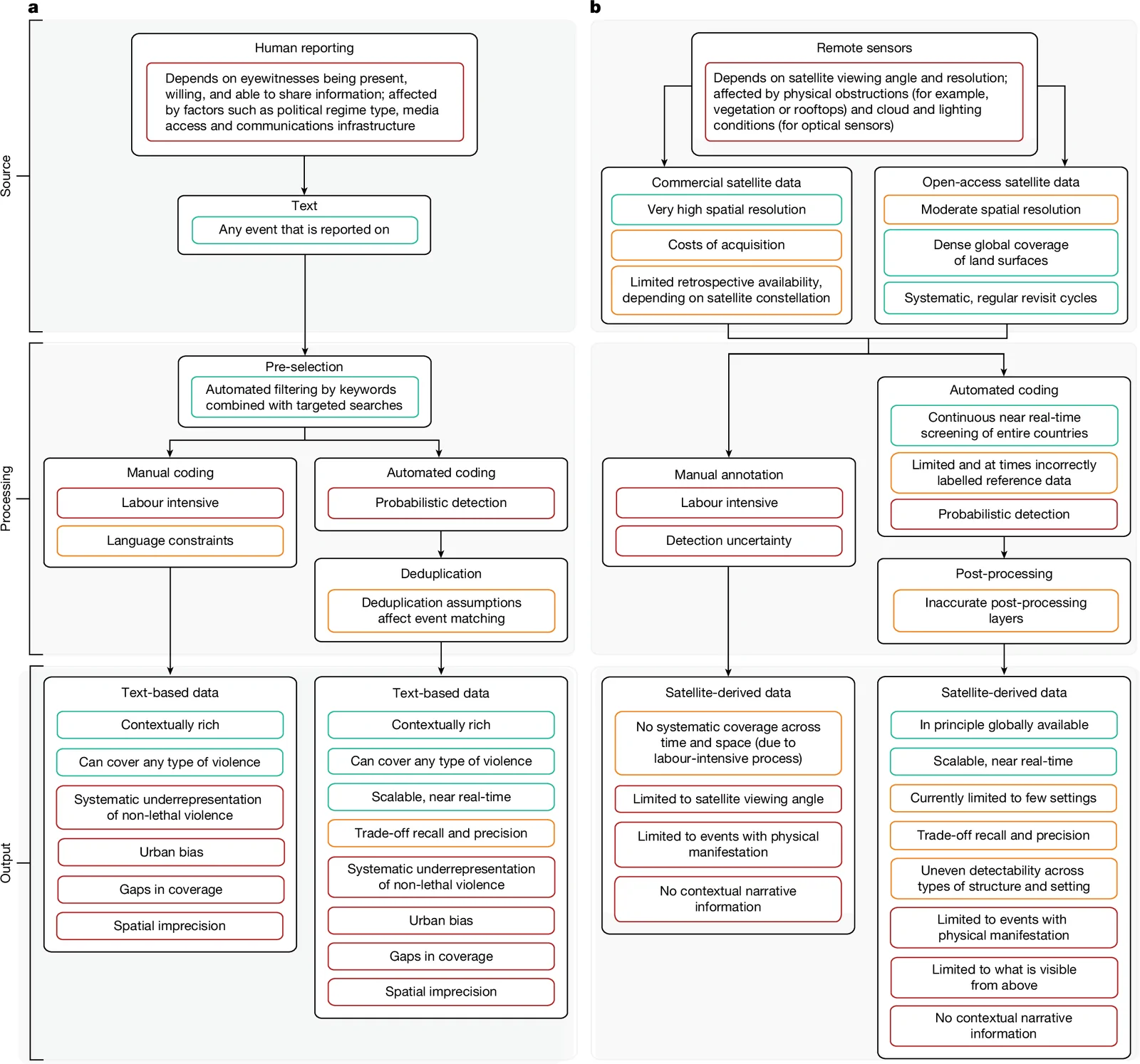
Data generation processes and associated limitations. **a**, Text-based data. **b**, Satellite-derived data. Outlines indicate strengths (green), current challenges and limitations that may be mitigated through technical, conceptual or organizational progress (orange), and challenges and limitations inherent to each data generation process (red). Although specific steps vary across methodologies, the primary limitations remain the same.

Disaggregated conflict event data based on human reporting, which we refer to as text-based data, form the empirical backbone of most quantitative-comparative studies of conflict dynamics. Figure 1a shows how such data are typically produced. Most studies rely on manually coded data^14–16^ (Fig. 1a, left). Human coders record individual instances of violence based primarily on news articles and reports from sources on the ground^30^, documenting event location and date, involved actors and resulting fatalities. Advances in natural language processing and large language models have enabled automated approaches to detecting conflict events from text^31–34^ (Fig. 1a, right). These approaches are faster and scalable, and draw on sources in multiple languages, but introduce new challenges, most notably reduced corroboration and validation of events^4^ (Supplementary Information, section C).

Coverage varies across geography and time, shaped by factors such as regime openness, media presence and access to communication technologies^2–4,6,17,35^. Because reporting is attention-driven, these biases are particularly pronounced for non-lethal violence^14,17,18^, which often goes unreported or is reported only at aggregate level. As a result, datasets that aim to include events beyond fatalities^15,31,34,36,37^ tend to severely undercount them^14,17,18^. One core provider of disaggregated conflict data records only events that involve fatalities, precisely because of this bias^1,14^.

Neither manual nor automated advances can resolve these core limitations of text-based data, which arise from the uneven availability of human reporting. Professionalized coding procedures, source selection and transparency inform engagement with these biases^4^ but cannot overcome the underlying constraints^38^. Triangulating across event datasets only highlights varied blind spots in overall coverage^39^. Emerging approaches, such as crowd-sourced reporting platforms and digital fieldwork infrastructures^40,41^, bypass media intermediaries and may improve coverage of underreported forms of violence, such as sexual violence. However, these too depend on eyewitnesses being present, willing and able to share information.

### Satellite-derived data

We see promise in complementing existing conflict data with data derived from satellite imagery (Fig. 1b). Satellites collect data during conflicts independently of physical access or perceived newsworthiness. Images are acquired at known times and georeferenced, tying each pixel to an exact time and location. This enables data collection in areas where information has been difficult to access, with spatial precision beyond text-based sources, and on events that often go unreported, particularly violence against material targets.

In theory, satellites can capture a wide range of information related to violence: actors and instruments of violence, such as troop movements or weapons systems; direct effects of violence, such as destruction of the built-up and natural environment; and indirect effects, such as degradation of agricultural activity or decline in market activity (Supplementary Information, section A). However, automated detection of actors and their instruments at fine-grained spatial and temporal resolution carries a high risk of dual use^24^, and we do not advocate for public research in this area. Given its direct relevance to conflict research and response, and its complementarity to text-based fatality data, we focus on data capturing the direct effects of violence on the built-up and natural environment, which we term war damage. Capturing such damage matters because, although lives lost are among the most tragic consequences of war, they are neither the only nor always the most relevant indicator of violence severity. The torching of homes and destruction of livelihoods may produce few direct deaths if populations have fled, yet they reshape realities on the ground, with long-term societal consequences, and often contribute to large numbers of indirect conflict-related fatalities^42,43^.

Traditionally, war damage data were collected through manual annotation of very high-resolution satellite images (Fig. 1b, left), a costly and labour-intensive approach that limited the scope of such data collections. Progress in the automated analysis of multi-temporal satellite data^7–10,19–22^ now makes large-scale, long-term data collections feasible (Fig. 1b, right), even  though the field is still in its early stages. One area that has emerged as a strong field in its own right is the detection of building damage^8–13^. Other work examines violence targeting the natural environment, including deforestation to deny cover to armed groups and attacks on cropland and water resources to disrupt rural livelihoods^26,44–47^. Studies document vegetation damage from rocket fire and munitions detonations^48–50^, fires set to trees and crops^26,51^, and direct vegetation clearance^52^ (Supplementary Information, section B).

Satellite-derived data also face important constraints. Whereas a decade of scrutiny has produced a clear understanding of the limitations of text-based conflict data^2–6^, satellite-derived data have been less systematically analysed, given the recency of the field. We see three main types of limitation.

The first type arises from limited reference data to train and validate war damage detection. As a result, approaches are fine-tuned for only a small number of conflicts, and we lack methods and analysis-ready data for information-poor settings, precisely where satellite-derived data would be most valuable. The availability of reference data is linked to international attention, but we can deliberately expand reference data and invest in approaches that generalize to underrepresented conflicts. Moreover, satellite archives span decades, meaning that even when events go undetected or patterns are overlooked, satellite data provide a record of the effects of armed conflict that can be analysed retrospectively as methods improve.

The second type arises because events are not equally detectable across forms of violence and settings. For example, in peripheral areas, buildings are often more isolated, smaller and surrounded by vegetation, making it more difficult to identify destruction. For automated building damage assessments based on moderate resolution data, this results in an urban bias that mirrors that of text-based data, even though it arises from a different mechanism. Technical improvements such as multi-modal approaches or higher-resolution open-access imagery can mitigate this limitation over time. For now, researchers must account for these biases, especially when they correlate with those of text-based data.

The third type differs from the first two in that it is inherent to the source and cannot be overcome through technical, conceptual or organizational innovation. Satellites can only capture phenomena that have a physical presence or visible effect on the Earth’s surface, and they cannot provide contextual narrative information. Similar to the inherent limitations of text-based data, which arise from the uneven availability of human reporting, this third type of limitation reflects the nature of the source itself rather than the methods of analysis. The implication is clear: advancing the field rests on our ability to leverage the strengths and mitigate the limitations of each data type through data integration.

## Data integration

The inherent limitations of single-source data motivate different forms of integration. Prior research on data integration in conflict research^39,53^ has focused on combining text-based event datasets, emphasizing their complementary coverage and use for cross-validation and enrichment across sources. Focusing on integration at scale, we propose three main approaches for combining text-based and satellite-derived data; the choice depends on the gap that needs to be bridged. Improvement addresses cases in which one data type covers the violence of interest but lacks a specific feature, such as the spatial precision missing from text-based data or the contextual narrative information that is absent from satellite-derived data. Enrichment is needed when no single source captures the full scope of relevant violence, as in campaigns that combine killings, destruction and displacement. Fusion, the deepest level of integration, jointly models text and satellite data to infer phenomena that neither source captures directly.

We illustrate these approaches in two contrasting contexts: Ukraine, an information-rich setting where automated war damage data are available, and Myanmar, an information-poor setting where we rely primarily on manually annotated data. Integration adds value in both contexts, although the gaps that it bridges differ. In information-poor environments, where text-based data are sparse, satellite-derived data carry more of the analytical weight. Conversely, some integration applications require fine-grained text-based data and are most feasible in information-rich settings.

### Improvement

The inherent limitations of single-source data (Fig. 1) mean that even when we focus on a specific form of violence, such as lethal violence or the targeting of livelihoods, reliance on one data type can yield biased results or preclude certain research questions. For example, analysing neighbourhood-level diffusion of violence or identifying targeting patterns requires spatial precision that text-based fatality data typically lack. Improvement addresses such cases by using one data type to add the missing feature to another, enabling new forms of analysis.

**Fig. 2:**
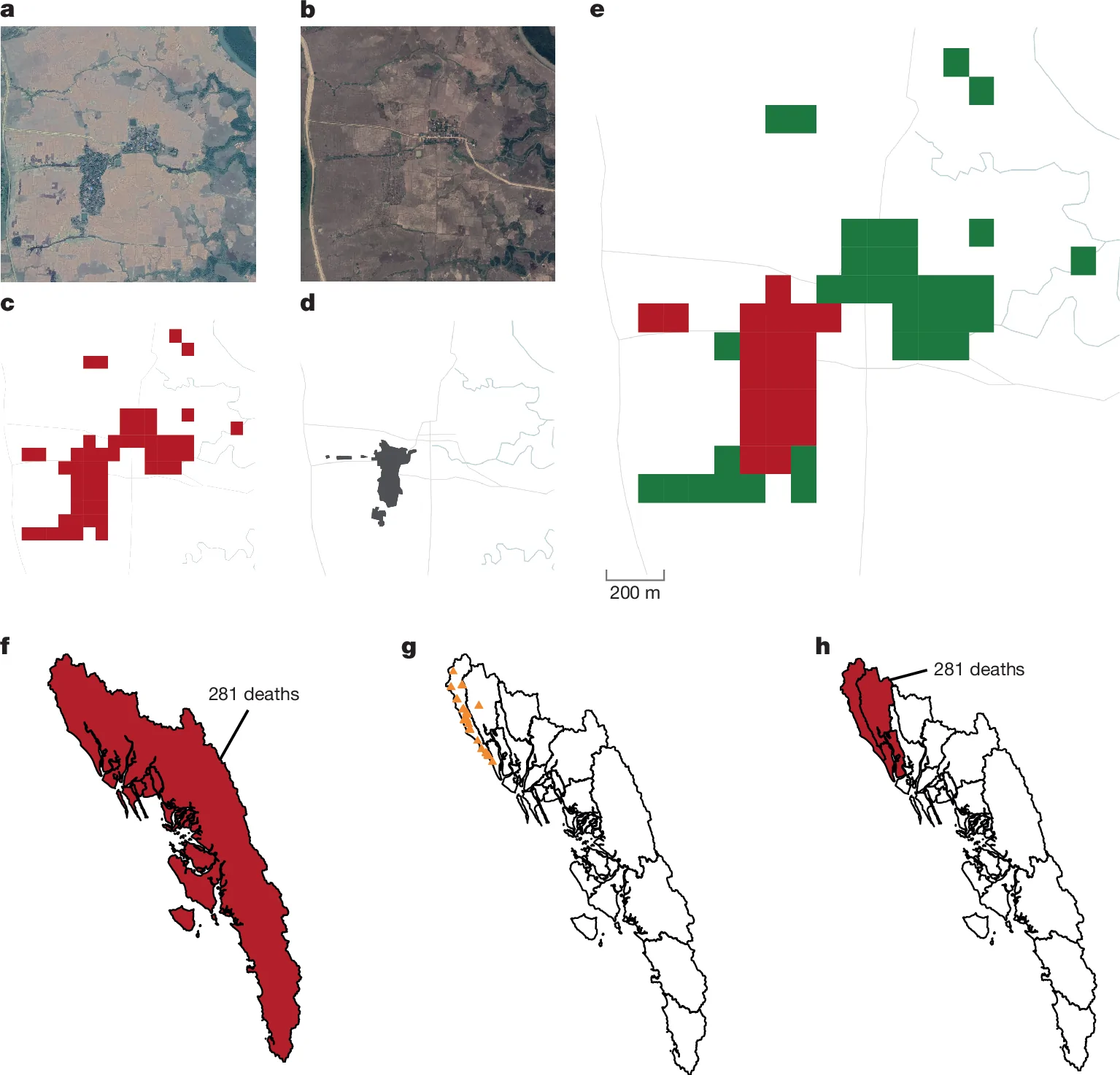
Spatial violence attribution in Rakhine State. **a**–**e**, Chut Pyin, Rakhine State. **a**, Pre-event optical imagery (27 December 2016). **b**, Post-event optical imagery (19 April 2019) showing selective destruction. **c**, Spatial distribution of reported fatalities at the settlement level. **d**, Satellite-detected destruction footprints. **e**, Refined fatality locations (red) derived from intersecting reported events with destruction data, excluding unaffected areas (green). **f**–**h**, Rakhine State. **f**, Fatalities recorded only at the Admin 1 (state) level. **g**, Thermal anomalies detected between 25 August and 9 September 2017 based on National Aeronautics and Space Administration (NASA) Fire Information for Resource Management System (FIRMS) data. **h**, Improved attribution of state-level fatalities to specific sub-districts on the basis of thermal anomalies. **a** and **b** reproduced from Google Earth, CNES/Airbus and Maxar Technologies (2025). **c**–**e**, Basemap adapted from OpenStreetMap contributors, ODbL (https://opendatacommons.org/licenses/odbl/1-0/). Data sources: refs. ^1,14,70–72^.

**Extended Data Fig. 1:**
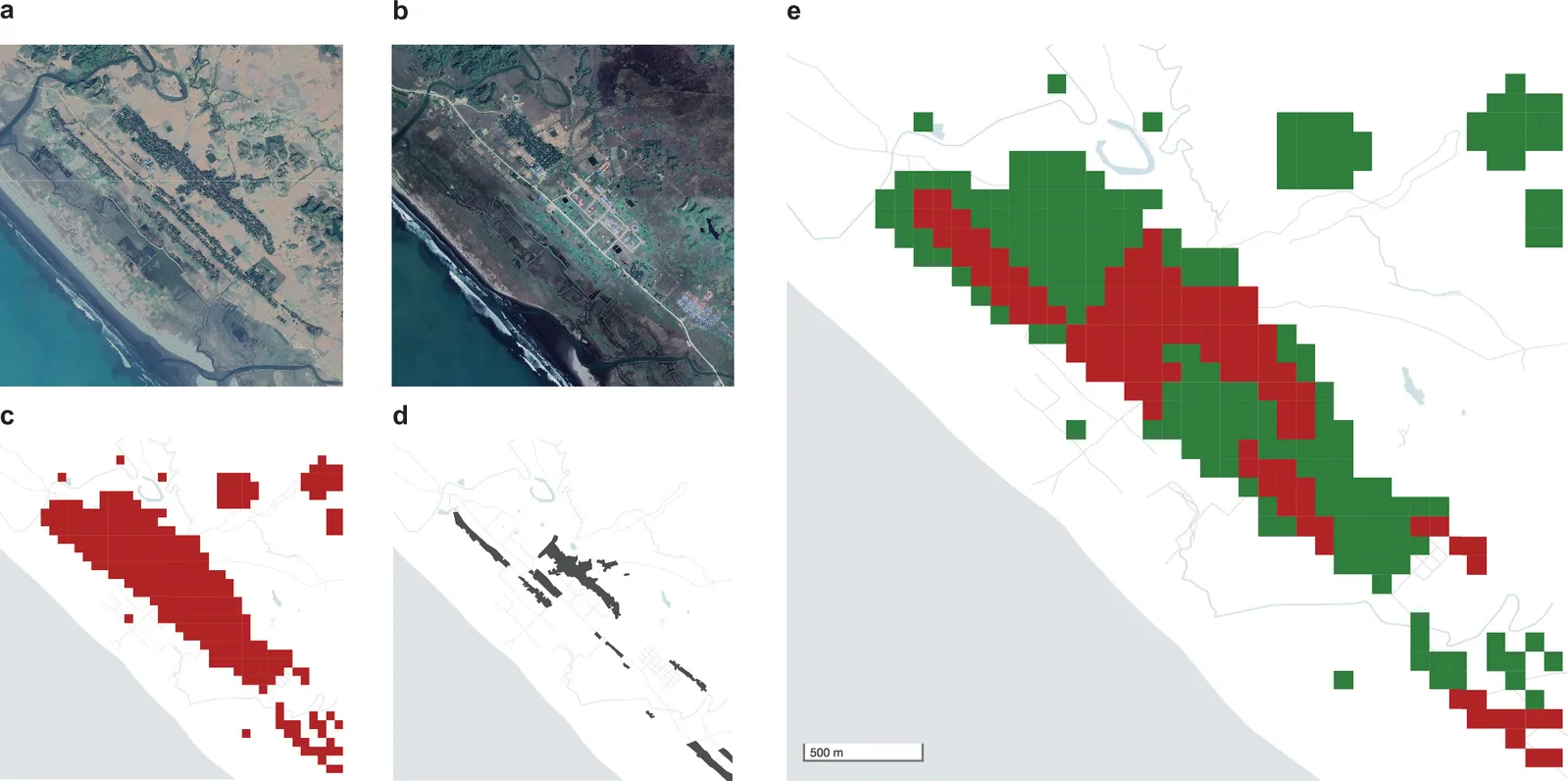
Local-level spatial violence attribution in Inn Din, Rakhine State. **a**, Pre-event optical imagery (27 December 2016) of Inn Din, a mixed-ethnicity village. **b**, Post-event optical imagery (1 October 2020) showing selective destruction. The entirely destroyed areas are Rohingya areas within the village, while the ethnic Rakhine area in the North of the village is left completely intact^56^. **c**, Spatial distribution of reported fatalities at the settlement level: all populated cells are presumed affected. **d**, Satellite-detected destruction footprints. **e**, Refined fatality locations (red) derived from intersecting reported events with destruction data, excluding unaffected areas (green). Images: Google ©2025 CNES/Airbus. Basemap: © OpenStreetMap contributors, ODbL. Data sources:^1,14,70–72^.

**Extended Data Fig. 2:**
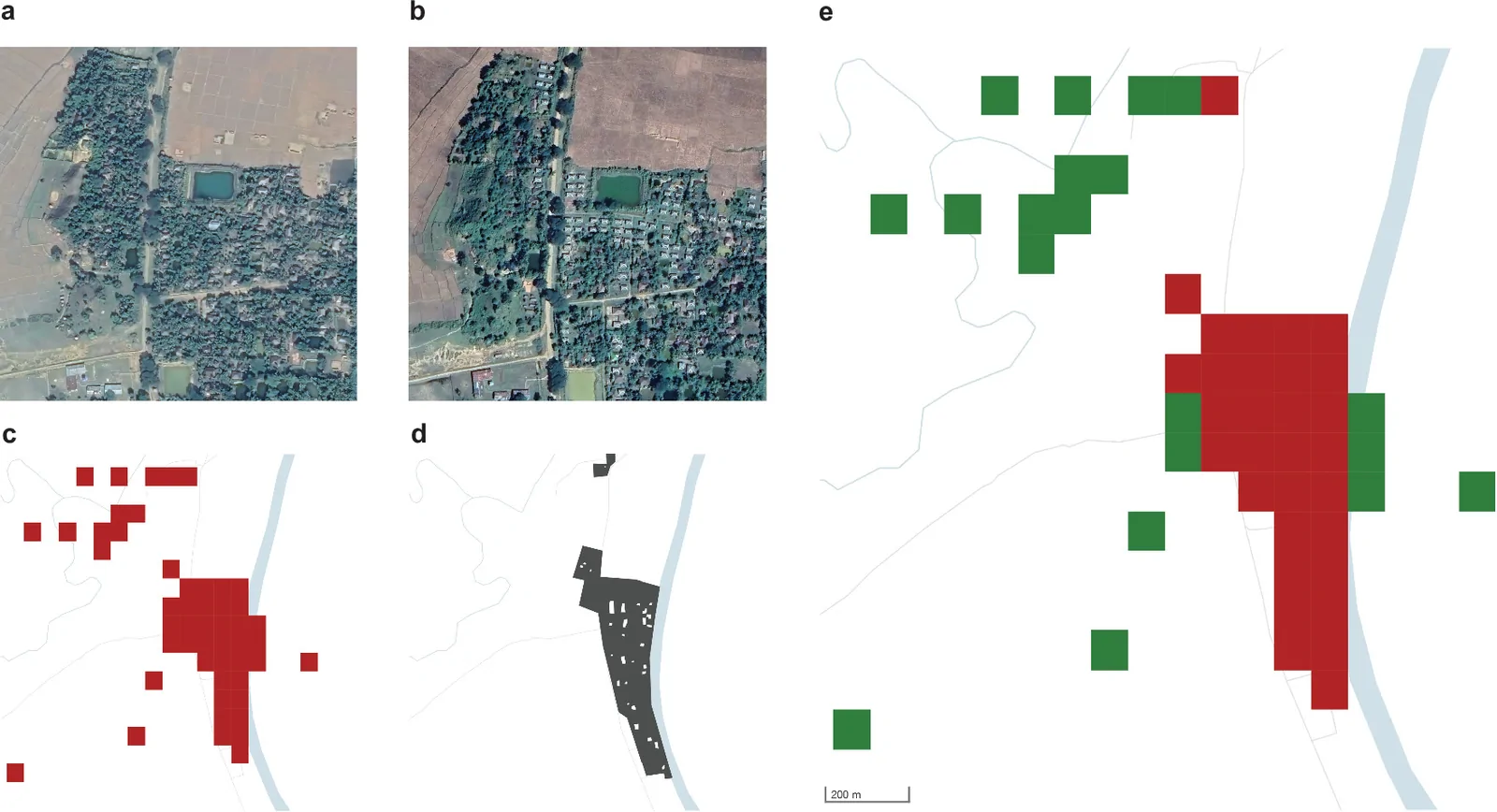
Local-level spatial violence attribution in Gu Dar Pyin, Rakhine State. Gu Dar Pyin was the site of a mass killing involving several hundred casualties^1,14^. **a**, Pre-event optical imagery of the north end of the village (27 December 2016), showing traditional dwellings in irregular spatial arrangements and partly covered by vegetation. **b**, Post-event optical imagery of the north end of the village (26 November 2019) showing newly constructed houses with highly uniform designs arranged in a regular pattern, suggesting coordinated planning. **c**, Spatial distribution of reported fatalities at the settlement level: all populated cells are presumed affected. **d**, Satellite-detected destruction footprints. **e**, Refined fatality locations (red) derived from intersecting reported events with destruction data, excluding unaffected areas (green). In this case, the spatial data show the almost complete destruction of the village. The before-after images show the subsequent transformation of the village in a pattern that is consistent with reports of coordinated resettlements of ethnic Rakhine populations following the displacement of the original inhabitants^82^. Images: Google ©2025 CNES/Airbus. Basemap: © OpenStreetMap contributors, ODbL. Data sources:^1,14,70–72^.

A key opportunity is to use satellite-derived data to improve the spatial precision of text-based data (for a related approach, see the literature on dasymetric mapping^54,55^). In many contexts, armed actors pursue specific objectives rather than engaging in violence at random; knowing where they fight is central to understanding the logic of violence. Because satellite-derived data are spatially explicit, we can use them to refine the locations of text-based data, provided that the form of violence captured in the text-based data co-occurs with the form captured in the satellite-derived data (Supplementary Information, section C)—for example, killings that take place alongside dwelling destruction. We illustrate this with examples from violence against the Rohingya people in Myanmar at the local (Fig. 2a–e) and more aggregate (Fig. 2f–h) levels. Our local-level example focuses on Chut Pyin in Rakhine State, where several hundred people were killed in a large-scale massacre; Extended Data Figs. 1 and 2 provide two additional village-level examples.

Text-based data^14,15^ form the backbone of our understanding of this violence and the actors involved, but are spatially imprecise. The best available fatality data are reported at the settlement level (Fig. 2c, red raster footprint). Because violence during this period involved coordinated attacks—killing civilians and destroying their homes in the same operation—we use satellite-derived war damage data (Fig. 2d) to indicate where fatalities are likely to have occurred. This enables attribution of fatalities to specific neighbourhoods (Fig. 2e). In this case, the divided spatial pattern, revealed only through improved spatial precision, points to ethnic targeting, indicating that Rohingya civilians were killed, whereas ethnic Rakhine residents in the same settlement were probably spared^56^. This process can be done at scale, contingent on the availability of satellite-derived data on destruction (Supplementary Information, section D). Because text-based data are typically limited to settlement level, local-level improvement opens up within-settlement variation analysis across information environments.

Figure 2f–h illustrates an approach to improving the spatial precision of text-based data at a more aggregate level. During the peak of lethal violence in Rakhine State, a substantial share (13%) of reported fatalities was coded only at the state level (Fig. 2f), forcing researchers to analyse at coarse spatial scales—where results are less meaningful—or to exclude these events, risking biased inference. Because most killings in this period occurred alongside the burning of Rohingya houses^56^, we use satellite-derived fire data to narrow down the area where these events are likely to have taken place. In this case, fire data (Fig. 2g) point only to the three sub-districts already identified through spatially precise data. We can infer that the imprecisely coded events took place in the same areas (Fig. 2h). This type of aggregate-level refinement is particularly valuable in information-poor settings, where a larger share of reported events is likely to be coded at coarse spatial units. The refinement enables analysis at smaller geographic units, allowing researchers and policymakers to identify where violence is actually unfolding rather than where it is reported.

**Fig. 3:**
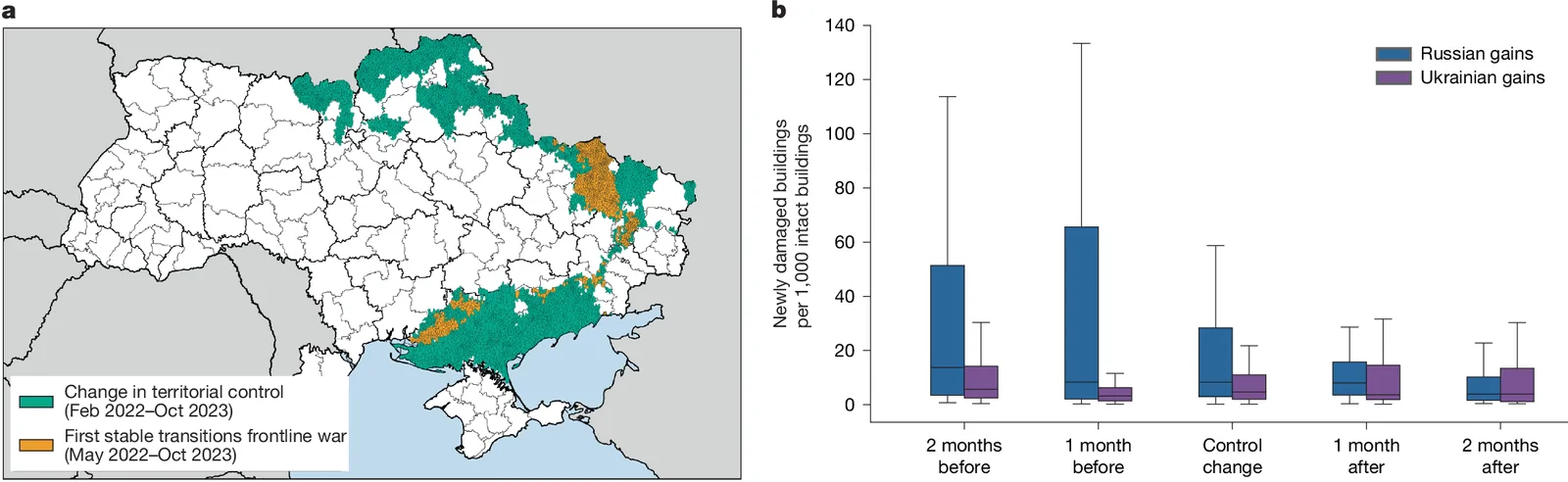
Building damage associated with territorial gains in Ukraine, frontline war. **a**, Areas with at least one change in territorial control (February 2022 to October 2023), overlaid with the first stable transitions occurring during frontline war (May 2022 to October 2023;  Methods). **b**, Box plots of newly damaged buildings per 1,000 intact buildings (start of month) across populated areas, in a five-month window centred on the month of control change, stratified by Russian (blue) and Ukrainian (purple) gains (May 2022–October 2023). Centre line shows median, box spans interquartile range and whiskers extend to the most extreme observation lying within 1.5 × IQR of the nearest edge of the box. These include only areas containing mapped building footprints with first stable transitions and radar-detected damage (Methods). The asymmetry is statistically significant from two months before (*m*_−2_) to one month after (*m*_+1_) control change and converges by two months after control change (*m*_+2_) (Kolmogorov–Smirnov, two-sided, *m*_−2_: *D* = 0.313, *P* = 0.008; *m*_−1_: *D* = 0.372, *P* < 0.001; *m*_0_: *D* = 0.237, *P* = 0.039; *m*_+1_: *D* = 0.275, *P* = 0.007; *m*_+2_: *D* = 0.151, *P* = 0.367). A Mann–Whitney test confirms the same pattern; full results in Extended Data Table 2, spatial autocorrelation analysis and its interpretation in Supplementary Table 1. The full time series is shown in Extended Data Fig. 3, and phase-specific analyses associated with Russian gains are presented in Extended Data Fig. 4. Data sources: refs. ^9,73,74^.

**Extended Data Fig. 3:**
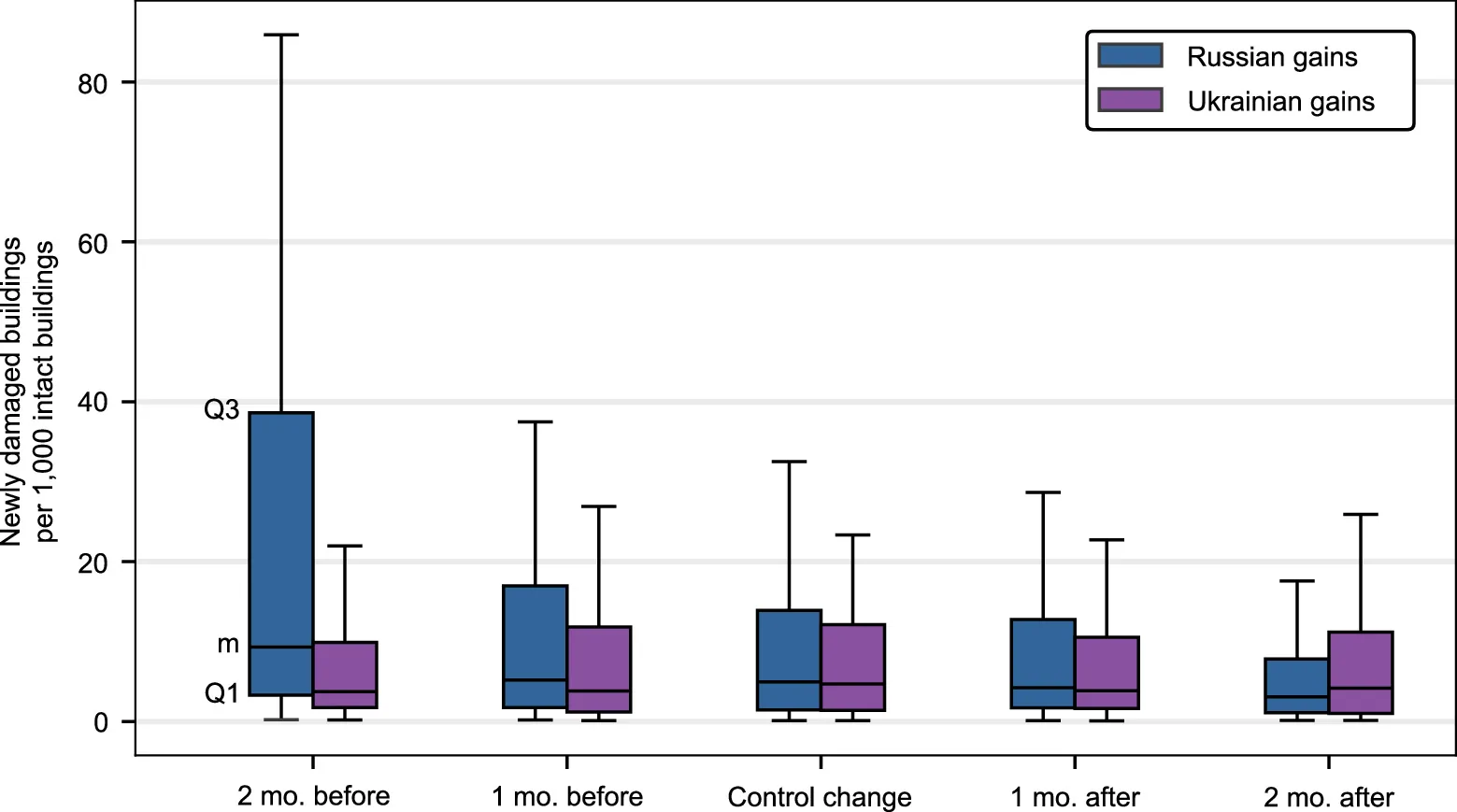
Building damage associated with territorial gains in Ukraine, full time series. Box plots of newly damaged buildings per 1,000 intact buildings (start of month) across populated areas, in a five-month window centered on the month of control change, stratified by Russian (blue) and Ukrainian (purple) gains from February 2022 to October 2023. Includes only populated areas with first stable transitions and damage; see Methods. Data sources:^9,73^. Centre line shows median, box spans interquartile range and whiskers extend to the most extreme observation lying within 1.5 × IQR of the nearest edge of the box.

**Extended Data Fig. 4:**
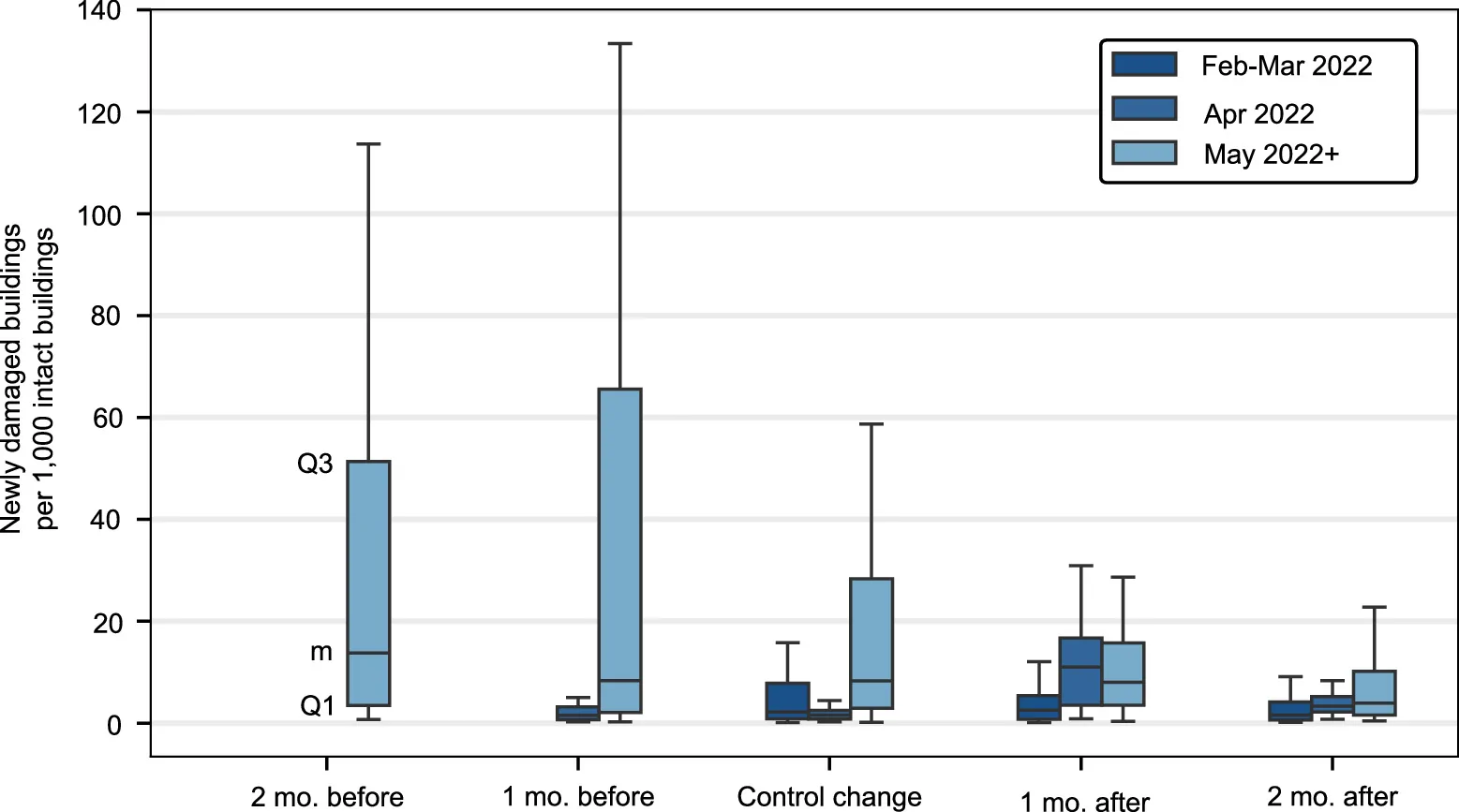
Building damage associated with Russian territorial gains in Ukraine, by operational phases. Box plots of newly damaged buildings associated with Russian territorial gains, per 1,000 intact buildings (start of month) across populated areas, in a five-month window centered on the month of control change, disaggregated across three distinct operational phases: the initial invasion (February–March 2022), Russia’s strategic reorientation toward the Donbas around April 2022, and subsequent frontline war (May 2022–October 2023). These phases reveal substantial variation in destruction intensity associated with Russian advances. Note that our damage data begin in March 2022. The absence of pre-invasion observations is unlikely to substantially affect these patterns, as the full-scale invasion represented an abrupt military escalation in most captured territories. Includes only populated areas with first stable transitions and damage; see Methods. Data sources:^9,73^. Centre line shows median, box spans interquartile range and whiskers extend to the most extreme observation lying within 1.5 × IQR of the nearest edge of the box.

**Extended Data Fig. 5:**
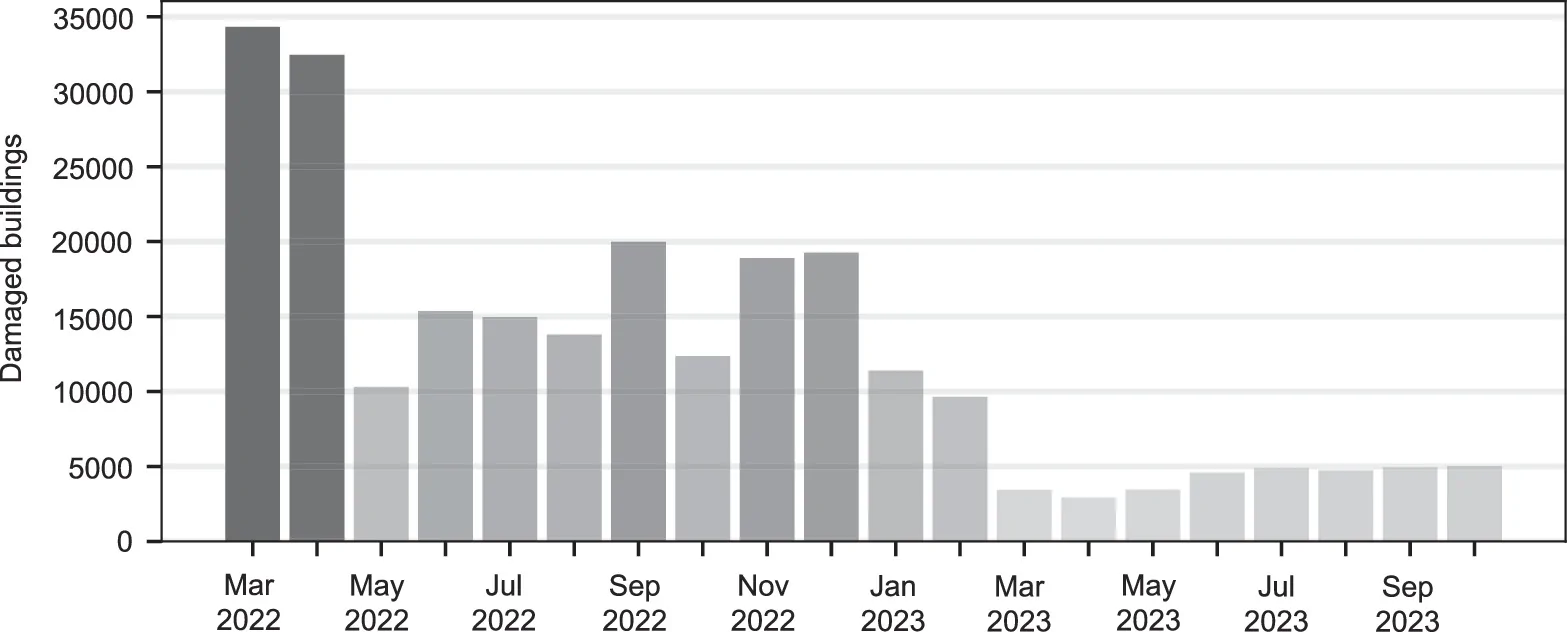
Timeline of building damage in Ukraine. Total detected newly damaged buildings per month (March 2022 to October 2023). Data sources:^9^.

Conversely, text-based data can enhance satellite-derived analysis by capturing contextual information. Details about perpetrators, victims or the context of an event can be drawn only from text-based data. In information-rich settings where fine-grained text-based data are available, this enables insights that are not possible with satellite-derived data alone. We demonstrate this through an analysis of building destruction in Ukraine during the frontline war (Fig. 3; see Extended Data Fig. 3 for full time series and Extended Data Fig. 4 for disaggregation of Russian gains into operational phases). Although satellite-derived war damage data reveal the spatio-temporal distribution of building destruction, only the integration of text-based territorial control data exposes asymmetries in targeting patterns between Russian and Ukrainian forces. During Ukraine’s 2022 counteroffensive, for example, the absolute number of newly destroyed buildings remained high (Extended Data Fig. 5). Comparing first stable transitions during the frontline war (Methods), our analysis shows that populated areas gained by Russian forces experienced building damage more often than those retaken by Ukrainian forces. In the month of territorial transition, 70.5% of populated areas gained by Russia exhibited newly damaged buildings, compared with 43.9% of populated areas regained by Ukraine (*χ*^2^(1) = 16.5, *P* < 0.001, *φ* = 0.26; full results for the 5-month window are in Extended Data Table 1). Where damage occurred, it was also more severe: among the subset of areas that sustained damage, Russian gains experienced more building destruction than Ukrainian gains, from two months before to one month after the control change (Fig. 3b; statistical analysis in Extended Data Table 2). These patterns remain hidden without text-based data on territorial control. Such integrated analyses, which single-source data cannot support, provide an empirical basis for assessing how different forces wage war and reveal differences that have systematic consequences for the scale of destruction endured by civilians.

### Data enrichment

Our second level of integration—enrichment—enables researchers to examine how different forms of violence relate to and capture spatio-temporal dynamics that cannot be observed from one data type in isolation. Unlike improvement, where one primary data source is supplemented by an auxiliary one, enrichment links text-based and satellite-derived data by a common time and location while keeping them analytically distinct.

A policy-relevant example is ethnic cleansing: actors target individuals from ethnic or religious groups through killings, rape and intimidation while simultaneously razing cultural or religious buildings and destroying homes. As people are attacked and livelihoods are destroyed, people are forced to flee, which subsequently reduces fatalities and violence against persons more broadly. Only data integration can capture these complex relationships.

**Fig. 4:**
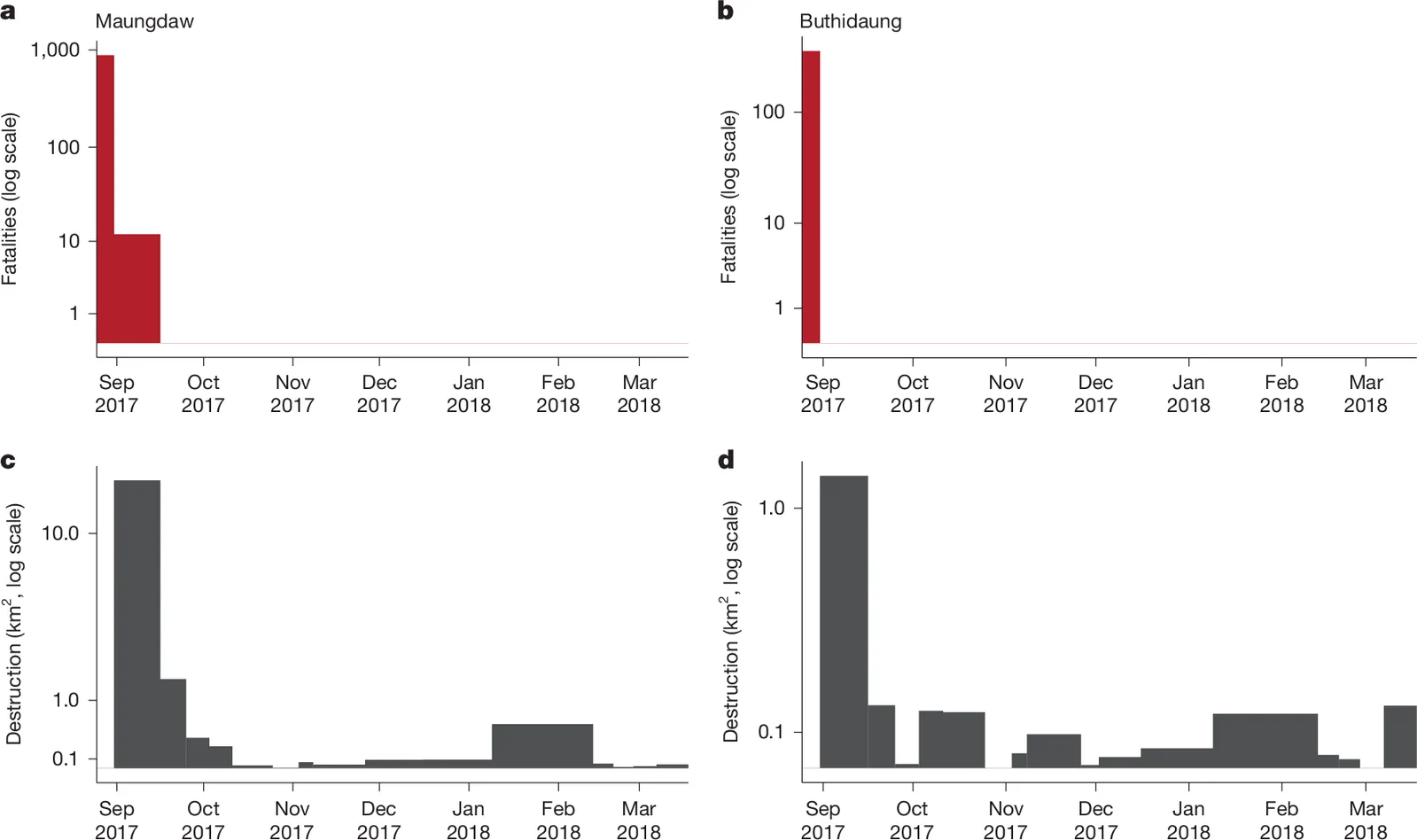
Killings and settlement destruction in Rakhine State. **a**,**b**, Reported fatalities (25 August 2017 to 18 March 2018; Uppsala Conflict Data Program (UCDP) Georeferenced Event Dataset (GED)) in Maungdaw township (**a**) and Buthidaung township (**b**). **c**,**d**, Settlement destruction (25 August 2017 to 18 March 2018; UNOSAT) in Maungdaw township (**c**) and Buthidaung township (**d**). Reproducing these panels with Armed Conflict Location and Event Data (ACLED) rather than UCDP GED data confirms the patterns (Extended Data Fig. 7). Note that *y*-axis scales differ across townships to accommodate varying absolute levels of violence. The campaign exhibited higher levels of violence in Maungdaw than in Buthidaung. Data sources: refs. ^1,14,71,72^.

**Extended Data Fig. 6:**
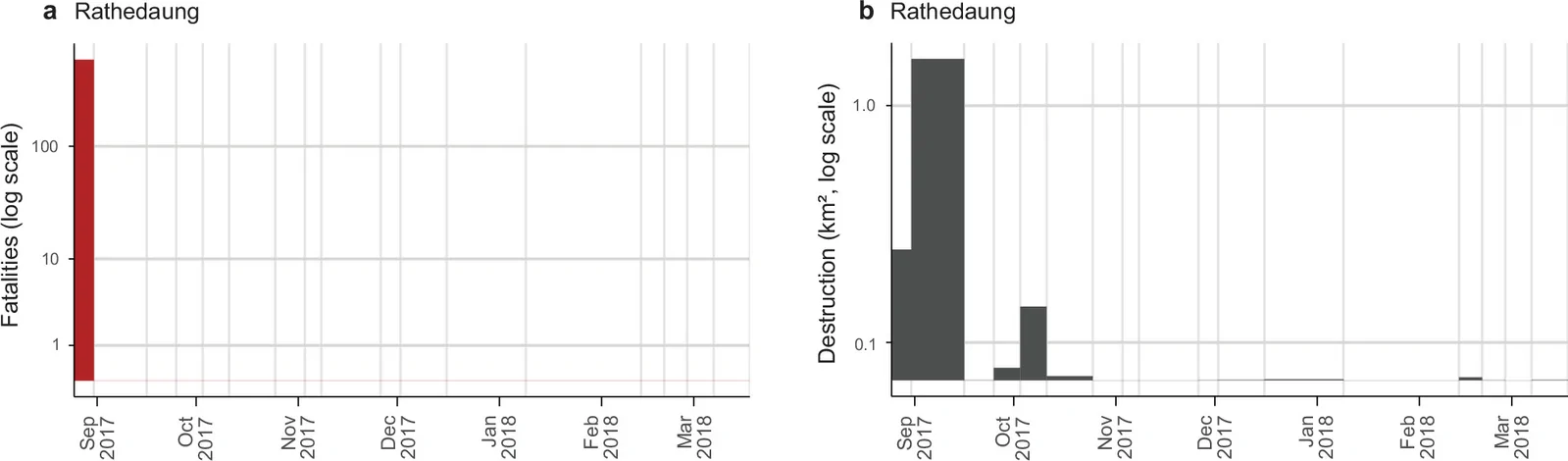
Killings and settlement destruction in Rathedaung township. **a**, Reported fatalities (25 August 2017 to 18 March 2018). **b**, Settlement destruction (25 August 2017–18 March 2018). Data sources:^1,14,71,72^.

**Extended Data Fig. 7:**
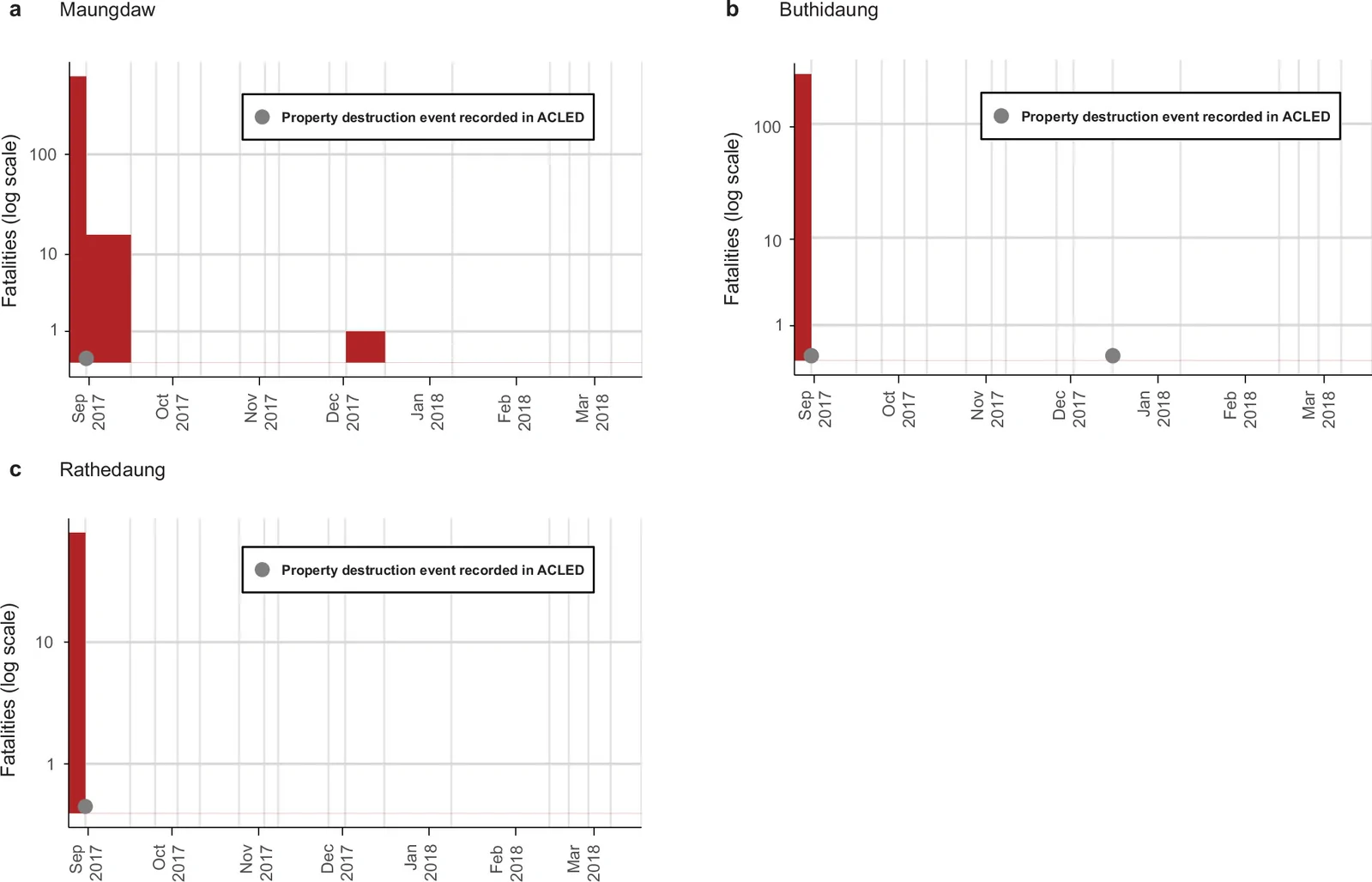
Fatalities in Maungdaw, Buthidaung, and Rathedaung townships with ACLED data. **a**, Maungdaw. **b**, Buthidaung. **c**, Rathedaung. All panels show reported fatalities between 25 August 2017 and 18 March 2018. Patterns closely mirror those in Fig. 4 and Extended Data Fig. 6, suggesting that results are robust across the two principal conflict event datasets. Grey dots indicate months with reports of property destruction in ACLED. Data sources:^15,72^.

Figure 4 illustrates how integrating fatality and war damage data changes our understanding of the ethnic cleansing campaign against the Rohingya population in Myanmar. Figure 4a,b shows that all reported killings in the two most affected townships occurred during the first week of the campaign (third-most affected township in Extended Data Fig. 6; same pattern using alternative conflict event data in Extended Data Fig. 7). Relying on fatality data alone would suggest that violence quickly subsided. Satellite-derived war damage data (Fig. 4c,d), however, reveal that violence continued for at least six months after the killing apparently subsided, albeit with a clear shift in form and intensity. As hundreds of thousands fled amid reports of violence and massacres, the government proceeded to remake Rakhine State by bulldozing the remains of burned villages, clearing trees and other vegetation, and in some cases constructing new security bases or villages reserved for other Rakhine communities^56^. This phase of the campaign is consequential for the surviving Rohingya population, as it hinders their return and continued existence in Rakhine State. Viewed through fatality data alone, the campaign appears as a week of violence; integrated analysis reveals a months-long process of permanent displacement. In information-poor environments, satellite-derived data carry more of the analytical weight, and even more so when violence itself decreases reporting as populations are killed and flee.

**Fig. 5:**
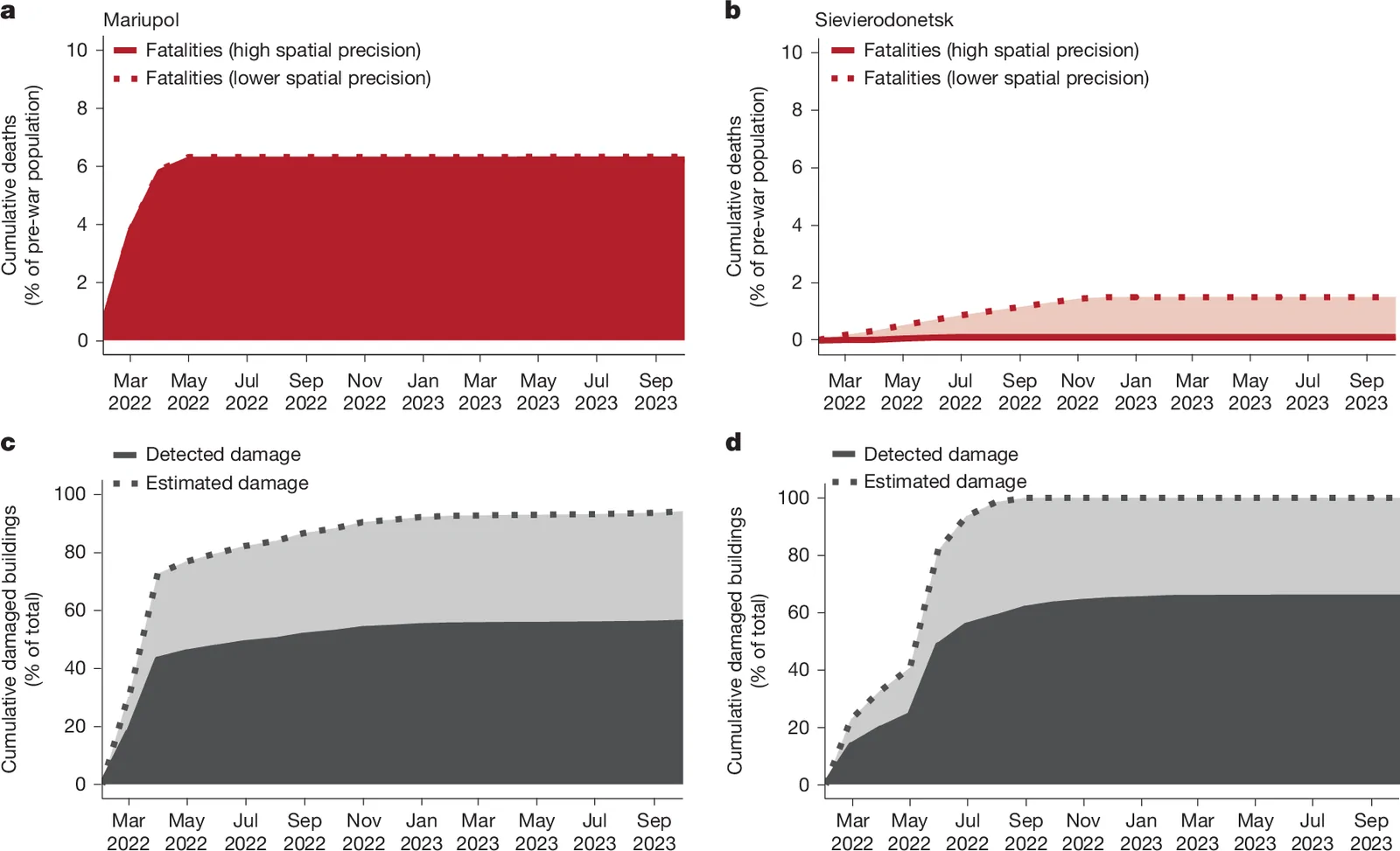
Scope of fatalities and building damage in Mariupol and Sievierodonetsk. **a**,**b**, Cumulative deaths in Mariupol (**a**) and Sievierodonetsk (**b**) as a percentage of the respective pre-war populations (data from February 2022 to October 2023). Solid lines include only events coded at settlement-level spatial precision; dotted lines additionally include events coded within 25 km. Both use UCDP GED best estimates. See Extended Data Fig. 8 for the same analysis with best and high estimates. **c**,**d**, Cumulative building damage in Mariupol (**c**) and Sievierodonetsk (**d**) as a percentage of the total number of buildings in each city (data from March 2022 to October 2023). Solid lines show detected damage and dotted lines show estimates adjusting for true positive rates (see Methods). Data sources: refs. ^1,9,14^.

**Extended Data Fig. 8:**
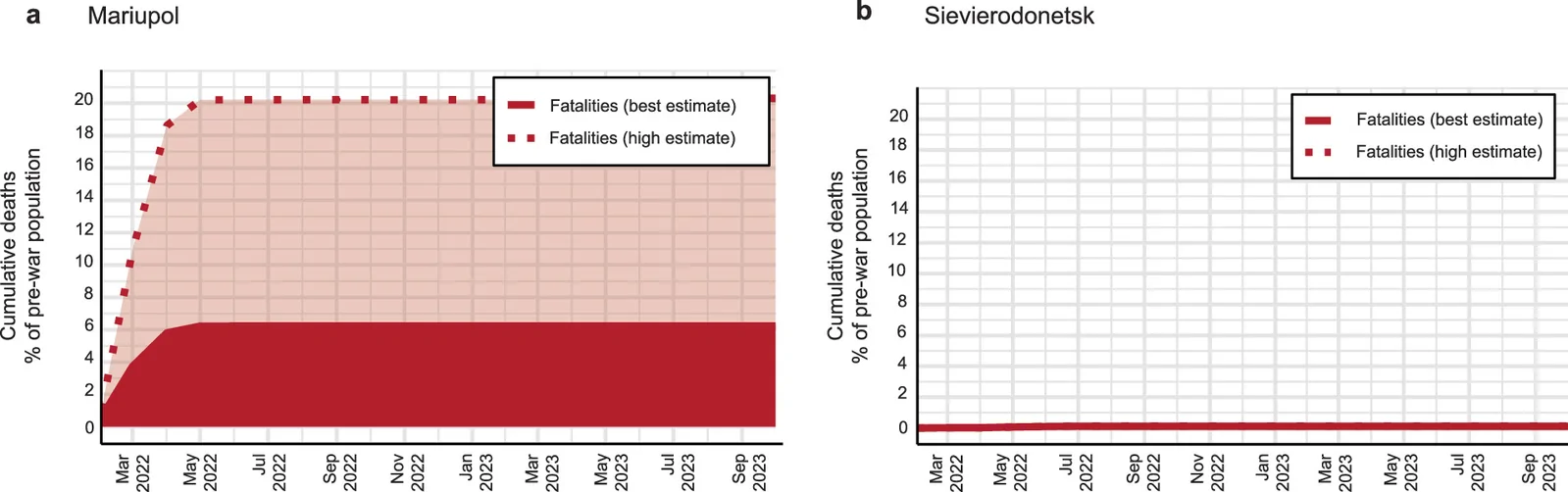
Cumulative fatalities in Mariupol and Sievierodonetsk, best and high estimates. **a**, Mariupol. **b**, Sievierodonetsk. Panels show cumulative deaths as a percentage of the pre-war population, including best and high estimates, for the period February 2022–October 2023. Only events coded at the settlement level are included. Data source:^1,14^.

Our second enrichment example, from Ukraine (Fig. 5; see Extended Data Fig. 8 for alternative estimates of fatalities), illustrates how, without data integration, we miss not only temporal but also spatial variation. The analysis compares text-based fatality data (Fig. 5a,b) and satellite-derived war damage data (Fig. 5c,d) for two large Ukrainian cities: Mariupol and Sievierodonetsk. Although both cities were devastated by Russian bombing and shelling, clear differences emerge, owing in part to the speed and timing of Russian advances. Mariupol was almost fully encircled within a week of the full-scale invasion, which hindered evacuation efforts and contributed to high casualties early on. By contrast, the Russian ground assault on Sievierodonetsk began in late May 2022, leaving more time to prepare evacuations. By the time violence peaked, only 10–15% of the pre-war population remained. Considering satellite-derived war damage data alone would obscure key differences in levels of lethal violence, whereas relying only on fatality data would miss much of the non-lethal violence endured by residents of Sievierodonetsk, including extensive destruction and sustained suffering by civilians who remained. The implications extend from research—such as understanding when destruction translates into mass casualties—to post-conflict planning. Both cities face massive reconstruction needs, but the populations who will return, and the trauma that they carry, differ profoundly.

Enrichment requires careful spatial and temporal aggregation or disaggregation, which involves consequential assumptions^57^ (Methods). Where conceptually meaningful, however, enrichment enables researchers to capture relationships between forms of violence that would remain invisible in single-source analyses.

### Cross-modal data fusion

Data fusion, our deepest level of integration, jointly models text and satellite data to infer an underlying phenomenon, such as violence in general, or specific manifestations such as civilian targeting. Whereas improvement and enrichment occur at the data output level (text-based and satellite-derived data), fusion occurs at the source level (text and satellite data), enabling the synthesis of insights across heterogeneous sources at scale. This makes fusion especially promising for information-poor settings, where patterns learned in data-rich contexts can support inference where direct evidence is sparse.

**Fig. 6:**
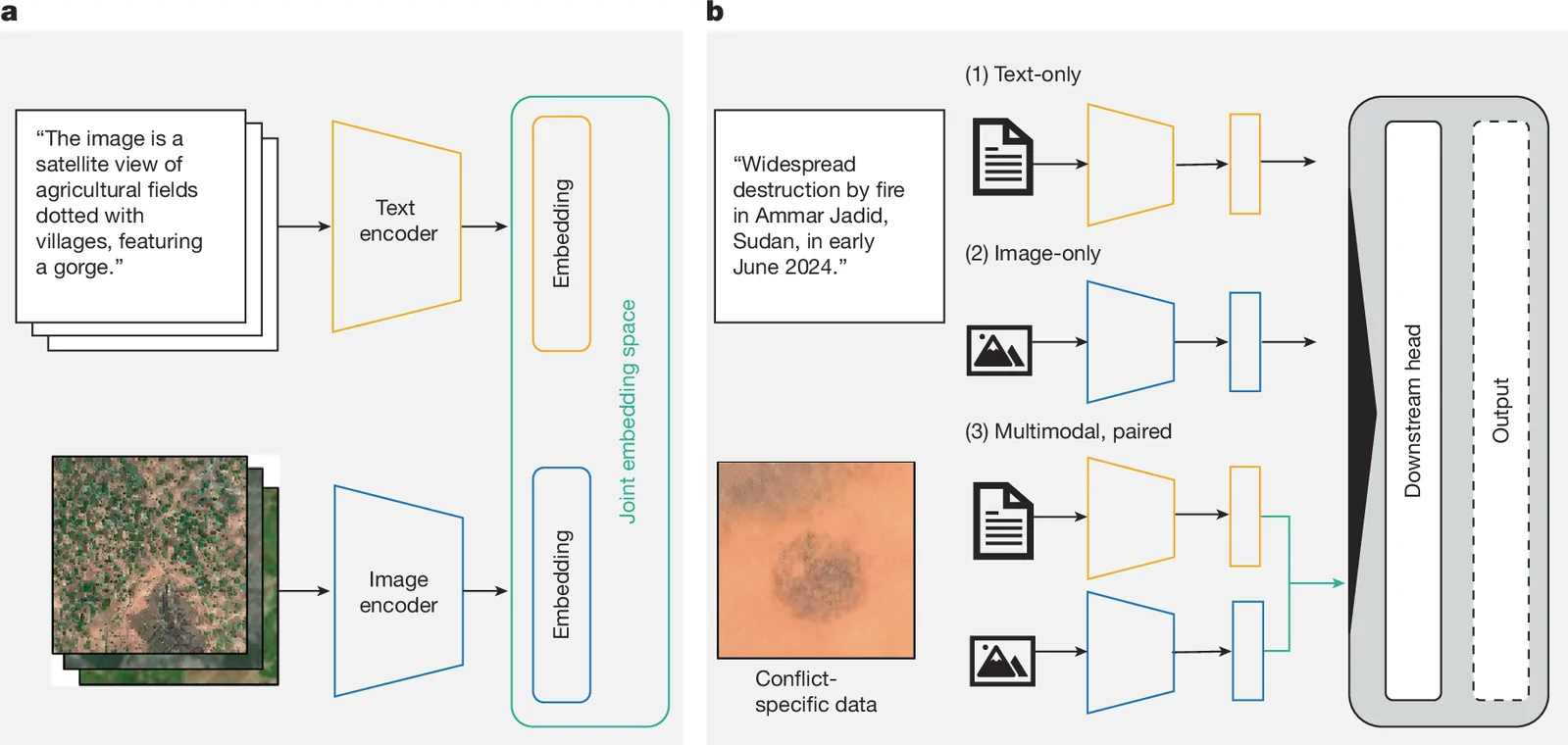
Vision–language pre-training and supervised fine-tuning under uneven data coverage. **a**, Pre-training learns vision and language encoders by aligning large-scale image–text pairs in a joint embedding space, bringing matched pairs closer and pushing mismatched pairs apart. **b**, Under supervised fine-tuning, pre-trained encoders are adapted to conflict-specific tasks using (1) text only, (2) image only or (3) paired image–text data, and connected to downstream task-specific heads to produce conflict-related outputs. Example imagery is derived from Copernicus Sentinel-2 data.

Although traditional data fusion has proven effective in other domains, the most promising path for conflict research lies in emerging vision–language foundation models (VLFMs)^58,59^, which generalize earlier fusion paradigms. Traditional fusion models combine input data sources (modalities) for specific tasks, relying on fixed, task-specific designs. By contrast, modern vision–language foundation models map different data types into a joint representation space (embeddings) using per-source encoders, learning relationships between concepts, space and time from co-located pairs (Fig. 6a). These general-purpose vision–language representations can be transferred to specific downstream tasks with minimal fine-tuning, enabling us to detect, classify, localize, and eventually understand conflict processes (Fig. 6b).

For example, campaigns of ethnic cleansing manifest through multiple tactics—killings, destruction and displacement—each of which leaves traces in different data sources. General-purpose joint modelling of these signals could enable fusion models to infer such campaigns even when individual indicators are sparse. Fusion approaches may also learn associations between textual descriptions and visual patterns that elude human analysts. In Myanmar, for instance, Rohingya and Rakhine communities typically construct distinct housing types^56^. After fine-tuning, a VLFM could learn associations between ethnicity, building typologies and targeting patterns. Spatially clustered destruction of one structure type may indicate group-based targeting, whereas destruction of individual buildings could signal selective violence^60^. Identifying such patterns at scale could advance long-standing debates about indiscriminate versus selective violence and inform policies to protect civilians under different targeting logics.

VLFMs are an emerging area and have not yet been applied to conflict research. The most mature applications are in medicine, where models trained on medical image–text data are fine-tuned to jointly interpret medical imaging and clinical text to detect disease processes^61–63^. In remote sensing, emerging approaches in areas such as ecology^64–66^ typically treat text as auxiliary to satellite imagery, rather than jointly modelling both as independent evidence of a latent phenomenon.

One challenge in adapting VLFMs for conflict research is that data vary widely in spatial and temporal resolution and exhibit uneven coverage across modalities, requiring careful spatio-temporal alignment^67^. Given the scarcity of conflict-specific aligned image–text pairs, training VLFMs from scratch on conflict data is unrealistic. The most promising path is to adapt pre-trained vision and language encoders^67^ through supervised fine-tuning with conflict-relevant data (Fig. 6). Such fine-tuning connects pre-trained vision and language encoders to task-specific downstream model blocks (heads) using conflict-relevant supervision, such as labels for detection, classification or localization. As shown in Fig. 6, encoders can be fine-tuned independently using text-only or image-only data when paired data are lacking, or jointly when aligned image–text annotations exist. This flexibility enables pre-trained representations to be tailored to conflict-relevant analytical objectives despite uneven data coverage. When image–text pairs are available, models learn cross-modal associations, combining the contextual richness of text reports and the localization of satellite images. Improvement and enrichment thus also help generate the aligned annotations needed for paired supervised fine-tuning.

Given its novelty and rapid development, fusion is likely to remain a methodological research frontier before it can be reliably deployed in conflict research or humanitarian applications. Beyond challenges of domain transfer, key concerns include explainability and trustworthiness^68^, as fusion may amplify biases in non-transparent ways^69^. Careful conceptualization of the target phenomenon is essential for data curation, model design and validation, especially where multiple processes co-occur (for example, natural hazard disasters and violence). Yet such settings may be where fusion adds most value, combining textual context with imagery to disentangle complex situations that single sources cannot.

Although foundation models for conflict research promise to be transformative, their development raises difficult ethical questions. With more narrow applications, researchers can control training data and impose targeted limitations. The versatile nature of foundation models, however, means that the same approaches used to understand conflicts and support populations in need could be repurposed to target vulnerable populations or identify military assets. Moreover, advances in generative AI now make it possible to create realistic synthetic satellite imagery. Although the risk to researchers using official providers remains low, this potential for forgery could erode public trust in satellite evidence and underscores the need for future authenticity certification. Addressing these risks requires careful ethical assessment, technical safeguards, and robust governance structures, which are likely to include gated release and access restrictions, particularly in early stages when risks remain poorly understood.

## Way forward

Given the strengths and inherent limitations of text-based and satellite-derived data, data integration is central to developing a more complete understanding of armed conflict. For now, integration at the output level—through improvement and enrichment—is the most feasible path, while data fusion promises even greater insights, especially in information-poor environments. Our aim of enabling large-scale analysis based on text and satellite data leads to two central priorities: expanding what automated approaches can capture across conflict settings and integrating satellite-derived data effectively with text-based data.

First, research with satellite data must prioritize approaches that generalize across forms of violence and conflict settings. Choices about which events and locations are used to train models directly shape what is detectable, and where. Current research focuses heavily on a few locations, such as Ukraine and Gaza, leaving many of the world’s conflicts overlooked. Although models are more difficult to train and validate in areas with limited reference data and informal settlements, ensuring that methods generalize is essential for systematic data collection and for humanitarian actors to respond to the needs of vulnerable populations.

Second, because the most fundamental limits of both data types are inherent to the source and cannot be overcome through innovation, researchers must pursue deeper interdisciplinary collaboration to establish the technical and conceptual foundations for data integration. Both technical expertise and social science insights are essential for adapting techniques to new settings and violence types, and understanding what the data represent. Once we pursue data integration, conceptual clarity becomes even more critical, because the deeper the integration, the richer the data become—but the more potential sources of error and misinterpretation we introduce, for example, when assumptions about spatial or temporal co-occurrence do not hold. Ultimately, broad and genuinely interdisciplinary perspectives are required to navigate the ethical challenges that this work inevitably raises and to ensure that data and code are developed, released and governed in responsible ways that protect vulnerable populations.

By pursuing this agenda across disciplinary boundaries, we can adopt a multi-modal, multi-dimensional approach that moves quantitative conflict research towards a fuller picture of violence in armed conflict—one that enables more effective protection of civilians and more informed policy responses.

## Methods

### Ukraine data collection

For our analyses of building destruction during the Ukraine war, we draw on three existing datasets.

#### War damage data

We use war-related damage data from ref. ^9^, which identifies probable damage across Ukrainian human settlements between March 2022 and October 2023. This dataset was generated using a coherent change detection framework applied to Sentinel-1 SAR data^75^.

#### Building footprints

Building footprint data were sourced from Overture Maps, providing a comprehensive dataset of 26,714,652 building footprints in Ukraine. The primary sources for these footprints include Microsoft ML Building Footprints (72.8% of buildings) and OpenStreetMap (27.2% of buildings).

#### Tessellated geometries

The unit of analysis is tessellated geometries around populated places. We use the polygons provided by the VIINA dataset^73^, which are based on the GeoNames gazetteer and include 33,141 tessellated geometries in Ukraine.

#### Boundaries

Spatial data on admin1 and admin2 boundaries, and settlement boundaries for Mariupol and Sievierodonetsk, are from State Scientific Production Enterprise ‘Kartographia’, as provided by the Humanitarian Data Exchange platform^74^.

#### Areas of control

For areas of control, we used the daily data provided by Violent Incident Information from News Articles (VIINA)^73^, which draws on four sources: VIINA event reports, DeepStateMap, the Institute for the Study of War, and Wikipedia.

#### Pre-war population data

Pre-war population statistics for Mariupol and Sievierodonetsk are taken from the State Statistics Service of Ukraine (2021, Number of Present Population of Ukraine).

#### Conflict event data

Information on fatalities is taken from the UCDP GED v25.1^1,14^.

### Ukraine data analysis

#### Building-level damage data

We disaggregate damage data based on ref. ^9^ to the building level using Overture Maps data. The original damage data are provided as 10-m projected pixel grids corresponding to built-up area identified by Global Human Settlement Layer^76^. We attribute damage to Overture building footprint data if a building footprint fully (>99%) overlaps with pixel-wise damage data. This resulted in 246,777 likely damaged Overture Maps building footprints.

#### Improvement section

To examine differences in building destruction before and after territorial control changes between Russian and Ukrainian gains (Fig. 3), we first identify changes in control at the level of tessellated geometries and then link these to the war damage data for the corresponding month–geometry combinations.

We aggregate control patterns to a monthly timestep to align with the monthly timestep of damage data. We classify a given month as under full control by one actor if that actor maintained control throughout the entire month; as transitioning from actor A to actor B if A controlled the territory on the first day of the month and B on the last day; and as ‘other’ for all remaining patterns, including contested status at the beginning or end of the month, or control by actor A at both the beginning and end but back and forth in control in between. This yields 569,606 month–geometry combinations under full Ukrainian control, 107,094 under full Russian control, 5,731 for Russian gains, 4,372 for Ukrainian gains, and 19,261 for contested or mixed control during the period of analysis (February 2022 to October 2023).

Next, we identify first stable transitions, defined as the first instance in which a territory transitions from Ukraine to Russia (Russian gains) or from Russia back to Ukraine (Ukrainian gains). A transition is considered stable if it was followed (and for Ukraine regaining territory, also preceded) by stable control. For Ukrainian gains, the territory had to be under full Russian control for at least two months before and under full Ukrainian control for at least two months after the transition. For Russian gains, we require at least two months of full Russian control following the transition. We do not require Russian gains to be preceded by two months of full Ukrainian control because the VIINA control data are only available from February 2022 onward. We are confident that Russian gains in the analysis had been under Ukrainian control prior to the transition because, by construction, month–geometry combinations for Russian gains must begin with Ukrainian control, which excludes territories already under Russian control in February 2022. To control for any buildings damaged prior to our analysis window, we only assess newly damaged buildings across the comparison window and further normalize per 1,000 undamaged buildings at the start of each month.

By focusing on clear and stable transitions, excluding areas that fluctuated between the two sides, and controlling for earlier building damage, we can more confidently attribute damage before and after control changes to Russian and Ukrainian gains separately. A total of 2,438 transitions meet our criteria for first stable Russian gains, of which 2,130 occurred in the initial phase of the invasion (February and March 2022), 108 in April 2022, and 200 between May 2022 and October 2023. For Ukraine, we identify 967 first stable transitions that meet our criteria, all of which fall in the period between May 2022 and October 2023. For the box plots in Fig. 3b and Extended Data Figs. 3 and  4, and the statistical analyses presented in Extended Data Tables 1 and 2 and Supplementary Table 1 in Supplementary Information, we restrict the analyses to populated areas, removing geometries with no buildings in our building layer. For Russian gains, this leaves 273 populated areas in February and March 2022, 29 in April 2022, and 95 between May 2022 and October 2023. For Ukraine, it leaves 148 populated areas between May 2022 and October 2023.

For the map of territorial control change (Fig. 3a), we visualize all first stable transitions that occurred during the frontline war (May 2022 to October 2023). In addition, we also visualize all tessellated areas that experienced at least one monthly change in territorial control during the study period. For the latter, we include areas that were (1) not under full Russian control in February 2022 but were under full Russian control for at least one month between March 2022 and October 2023, or (2) under full Russian control in February 2022 but were under full Ukrainian control for at least one month between March 2022 and October 2023.

To visualize the distribution of damage, we normalize the timelines by assigning month 0 to the month of the control change (first stable transition), with a five-month window spanning two months before to two months after (*m*_−2_ to *m*_+2_). Using the tessellated month–geometry combinations with normalized timelines, we attribute damage to Russian versus Ukrainian gains and visualize the distributions using box plots (Fig. 3b). Each box aggregates the newly damaged-building counts across the cells with at least one newly damaged building in that specific relative month. Cells with zero new damage in a given month do not contribute to that month’s box but may contribute to others.

To assess the difference in targeting patterns, we conduct a two-step statistical analysis. First, we compare, at each relative month in the five-month window (*m*_−2_ to *m*_+2_), the proportion of populated areas that experienced damage around control change between areas gained by Russia and those retaken by Ukraine (two-sided Pearson chi-squared test, results reported in the main text and Extended Data Table 1). Second, we conduct two-sided Kolmogorov–Smirnov and Mann–Whitney tests to compare levels of damage associated with Russian and Ukrainian gains, conditional on at least one newly damaged building in month *m* (reported in Fig. 3 and Extended Data Table 2). The results of the two-step analysis show that, in the months around the transition, Russian territorial gains are more often associated with building damage than Ukrainian gains (Extended Data Table 1), and the median number of newly damaged buildings per 1,000 intact buildings is roughly twice as high for Russian gains, a significant difference (Extended Data Table 2). To check the independence assumption underlying the per-month tests, we compute Moran’s *I* on log1p(newly damaged buildings) using a spatio-temporal weights matrix (full results and interpretation in Supplementary Information, section E).

The war damage data are available from March 2022 to October 2023. For the main analysis, we include only control changes from May 2022 onward, which is approximately when the frontline war began. Many sources date the beginning of the ‘Battle of Donbas’ to 18 April 2022; however, because our war damage data are at monthly resolution, we use May 2022 as the starting point. This approach has two advantages: it aligns with the temporal resolution of our damage data, and it ensures that damage data for the two months preceding control changes are available (as the damage data only extend back to March 2022). In the extended data, we present the same analysis for the full period covered by the war damage data (Extended Data Fig. 3) and further disaggregate Russian gains into three phases: the initial multi-front invasion, the reorientation phase and the frontline war (Extended Data Fig. 4).

#### Enrichment section

For the enrichment analysis presented in Fig. 5, we use the month-settlement unit as the common unit of analysis to compare fatalities and war damage over time. To identify relevant conflict events, we perform a spatial intersection between the UCDP GED event coordinates and the settlement boundaries. While the majority of fatalities were recorded with a temporal precision of one month or finer, some events involving high fatality counts were recorded with a lower temporal precision level (level 5), indicating a range exceeding one month. Excluding these data points would have distorted the overall level of fatalities, particularly in Mariupol. To maintain the month-settlement unit, we disaggregate these entries by distributing fatalities uniformly across the days within the reported date range.

Regarding spatial precision, our primary estimates include only events coded at the settlement level (the highest precision level). Given the documented urban bias in conflict event data, we are confident that most events occurring within Mariupol and Sievierodonetsk are captured at this level. However, to account for events occurring within settlement boundaries that may have been coded with lower spatial precision, we also provide a broader estimate using spatial precision level 2 (event occurred within 25 km), represented by a dotted line. All fatality figures are based on the UCDP GED ‘best’ estimates. In the Extended Data Fig. 8, we also provide ‘high’ estimates reported by UCDP GED (events coded at settlement-level precision only), which show even more pronounced contrasts in fatality levels between the two cities. To facilitate comparison between cities of different size, we report cumulative fatalities as a percentage of pre-war inhabitants, using 2021 population baselines.

The war damage data, originally reported at monthly intervals, required only spatial aggregation via intersection with the settlement boundaries. We report these results as the percentage of the total buildings affected, providing both detected damage and adjusted estimates based on reported true positive rates^9^, capped at 100%.

### Myanmar data collection

For our analyses of civilian targeting of the Rohingya population in Myanmar, we draw on five existing datasets.

#### Conflict event data

Information on violent events, including involved actors and reported fatalities, is sourced from the UCDP Georeferenced Event Dataset (GED) v25.1^1,14^. In Extended Data Fig. 7 we replicate our enrichment analyses using ACLED^15^.

#### War damage

To capture building destruction, we rely on damage assessment data produced by United Nations Institute for Training and Research (UNITAR)–United Nations Satellite Centre (UNOSAT), which record satellite-detected destroyed and damaged settlements in the Buthidaung, Maungdaw and Rathedaung townships of Rakhine State^71^. These data are based on a manual analysis of satellite imagery collected on multiple dates between 31 August and 11 October 2017.

#### Thermal anomalies

To identify thermal anomalies, we used data from the near-real time VIIRS 375 m Active Fire product (VNP14IMGT)^77^.

#### Populated areas

To distinguish populated from unpopulated areas, we use raster data from WorldPop^70^, drawing on the constrained estimates of total population per grid square at 3 arcsec resolution (approximately 100 m at the equator), R2025A v1. We use estimates for 2016—the year preceding the 2017 violent campaign—because violence in Rakhine State led to large-scale population displacement. To select thermal anomalies detected in or near populated areas, we also use village point locations from the Myanmar Information Management Unit (MIMU) v9.6^78^.

#### Administrative units

Spatial data on the administrative boundaries of Myanmar are taken from MIMU v01, via the Humanitarian Data Exchange platform^72^. We use administrative levels one (state), two (district) and three (township).

### Myanmar data analysis

#### Improvement section, settlement level

We conduct local analyses at the level of individual settlements (villages or towns) across multiple locations in northern Rakhine State. In a first step, we use text-based conflict event data (UCDP GED) to identify settlements in which government violence against civilians involving killings was reported during the initial phase of the 2017 campaign. The majority of lethal conflict events occurred between 25 August and 9 September 2017. Our analysis focuses on the window of 25 August to 16 September 2017, representing the narrowest temporal interval that captures the peak violence while also aligning with the capture dates of satellite-derived war damage data. We restrict the conflict event data to observations with the highest level of spatial precision—that is, events for which the affected settlement was recorded.

For each settlement, we define a settlement-specific spatial window based on the visually interpreted settlement extent. We then integrate gridded population data at 100 m resolution from WorldPop, clipped to each settlement’s spatial window, to delineate inhabited space prior to violence. Population cells with zero values are excluded, and the remaining cells are converted to polygons to enable spatial intersection with satellite-derived damage assessments. Prior to this intersection with the more spatially precise satellite-derived damage data, the entire populated polygon is assumed to be affected by violence.

To further refine this area, we incorporate UNITAR-UNOSAT damage polygons and restrict these data to observations dated on or before the second available satellite image acquisition date (16 September 2017). We spatially intersect the damage polygons with each settlement’s populated polygon to classify each populated image pixel within the spatial window as damaged or undamaged. This enables us to distinguish between settlements that experienced physical damage and those that did not within a tightly bounded local context, thereby refining the spatial footprint implied by settlement-level conflict event reporting.

The article presents the results of this analysis for one such settlement, Chut Pyin (Fig. 2), where a large-scale massacre occurred during the analysis period^56^. In Extended Data Figs. 1 and 2, we present the same analyses for additional settlements, and in Supplementary Information, section D we discuss how they can be conducted at scale. Study locations were selected based on the availability of satellite-derived damage assessments in an otherwise information-poor conflict environment.

#### Improvement section, state level

To illustrate how satellite-derived data can be used to improve spatial precision in cases where reported events are located at coarse administrative levels, we again focus on the 2017 violence in Rakhine State, Myanmar.

We restrict the UCDP GED conflict event data to events recorded in Rakhine State. Of the 54 events reported in 2017, 42 (78%) occurred within a two-week period between 25 August and 9 September 2017; we therefore limited our analysis to this time frame.

For approximately half of these events, we know within which village or town they occurred. All these events with high spatial precision occurred in Maungdaw and Sittwe districts in the northern part of Rakhine State. For an additional third of events, the district is known; in all cases, this is Maungdaw district. The remaining events—accounting for 281 reported fatalities (best estimate)—are recorded as occurring in Rakhine State without any sub-state location information.

To narrow down the areas likely affected by the violent campaign, we analysed active fire detections from the NRT VIIRS 375 m product (VNP14IMGT) between 25 August and 9 September 2017. These detections are point features, which we restricted to the Rakhine State boundary using administrative level-one shapefiles. We further intersected the fire detections with a 500-m radial buffer around settlement locations to differentiate potential village burning from wildfires. To further distinguish these events from non-conflict-related events, such as agricultural burning or industrial heat emissions, we conducted visual verification using publicly available historical very high-resolution imagery available through Google Earth and optical imagery from the Copernicus Sentinel-2 mission^79^. Fires were positively attributed to the campaign only when thermal detections co-occurred with visible evidence of settlement destruction, such as scorch marks, burned structures, or collapsed roofs.

We then aggregated confirmed thermal anomalies to townships by intersecting detections with third-level administrative polygons. We constructed a binary indicator for each township denoting whether it contained at least one confirmed thermal anomaly during the analysis period. This produced a state-wide map of administrative units with physical evidence consistent with widespread settlement burning. We detected thermal anomalies exclusively in the townships in which spatially precisely coded killings took place. No thermal anomalies were detected elsewhere in Rakhine State during the period under investigation.

Using this exclusion-based logic, we infer that the imprecisely coded conflict events are likely to have occurred in the same northern part of the state as the precisely located events, rather than elsewhere in Rakhine. Importantly, this process does not reassign individual events to specific locations. Rather, it demonstrates how satellite-derived data can be used to narrow the plausible geography of coarse event locations for descriptive and statistical downstream analyses in cases where lethal violence and the burning of structures spatially and temporally co-occur. Because VIIRS detections capture thermal activity rather than violence per se, and because fires may go undetected due to cloud cover or timing, the absence of detections should not be interpreted as definitive evidence of the absence of violence.

#### Enrichment section

For the enrichment analyses presented in Fig. 4 and Extended Data Fig. 6, we integrate text-based conflict event data from UCDP GED with satellite-derived damage data from UNITAR-UNOSAT into a single tabular dataset. The spatial unit of analysis is the township. We focus on three townships in northern Rakhine State—Maungdaw, Buthidaung and Rathedaung—for which UNOSAT data are available.

To compare fatalities and war damage over time, we first align the damage data and the conflict event data temporally. Damage assessments are tied to specific image acquisition dates, which are irregular over time. Conflict event data, by contrast, are temporally more fine-grained and, in the large majority of cases, report the exact date of an event. Because the UNOSAT data have the coarser temporal resolution, they define the temporal unit of analysis. In our case, the UNOSAT data contained 22 distinct acquisition dates, with intervals between acquisitions ranging from 1 to 35 days. For interpretability of the time series, we collapse closely spaced acquisition dates, resulting in 17 analysis periods with intervals between 5 and 35 days. These periods constitute the temporal unit of analysis used throughout the enrichment example.

To aggregate the conflict event data to these periods, we first subset the data to events that are known to have occurred in Rakhine State. For approximately 80% of events, the precise date on which they occurred is reported (temporal precision 1). The rest of events are recorded with lower temporal precision and information about the period within which they occurred. To allow alignment with the UNOSAT periods, we distribute fatalities uniformly across the days within the reported date range—a common procedure in conflict research for temporally disaggregating event data^80^. After constructing the daily conflict event data, we restrict the sample to the period of UNOSAT damage data availability (ending 18 March 2018). Although the violent campaign began on 25 August 2017, the first UNOSAT image was not acquired until 31 August 2017. Consequently, damage detected between the first two image acquisitions is attributed to the full period from the campaign’s onset to the date of the second image. We assign each daily observation to the corresponding UNOSAT acquisition period, enabling direct aggregation of fatalities to the satellite-defined temporal units.

Next, we spatially intersect UNOSAT damage polygons with township boundaries (administrative level 3) to assign each observation to a specific administrative unit. In instances where a single damage polygon spanned multiple townships, we retain only the fragment with the largest overlap to ensure unique assignment. We apply a similar filtering process to the UCDP GED conflict data, restricting the sample to events with spatial precision levels 1 (exact location) and 2 (known location within a 25 km radius). Events at precision level 3 (second-level unit known) are excluded as they are too spatially imprecise for our township-level analysis. This results in the exclusion of over half of the recorded events, underscoring how improvements in spatial precision (such as those demonstrated in Fig. 2) facilitate more advanced forms of data integration.

We then aggregate both datasets to a common township-period unit of analysis. For the damage data, we calculate the total destroyed or damaged area in m^2^ and convert to km^2^. For the conflict event data, we compute the sum of the best-estimate fatalities. To enable consistent comparisons over time, we complete both panels by explicitly adding missing township–period combinations and setting damaged area and fatalities to zero where no observations were recorded for a given township-period.

Finally, we merge the two datasets into a single township-by-period panel, which forms the basis for the township-level time series shown in Fig. 4 and Extended Data Figs. 6 and 7. Given the highly skewed distribution of both variables, we visualize both series using a log_10_(*x* + 1) transformation. This approach preserves zero values while facilitating comparison across periods of varying magnitudes. The main text presents the comparative plots for Maungdaw and Buthidaung townships. In Extended Data Fig. 6, we present the corresponding visualizations for Rathedaung, and in Extended Data Fig. 7 the fatality counts using ACLED data instead of UCDP GED.

### Reporting summary

Further information on research design is available in the Nature Portfolio Reporting Summary linked to this article.

## Online content

Any methods, additional references, Nature Portfolio reporting summaries, source data, extended data, supplementary information, acknowledgements, peer review information; details of author contributions and competing interests; and statements of data and code availability are available at https://doi.org/10.1038/s41586-026-11004-6.

## Supplementary information

Supplementary Information This file contains five sections: (A) Data on three aspects of violence; (B) War damage and human toll of conflict; (C) Data generation processes and limitations; (D) Automated spatial improvement; and (E) Spatial autocorrelation analysis (including Supplementary Table 1).

Reporting Summary

Peer Review file

## Figures and Tables

**Extended Data Table 1:**
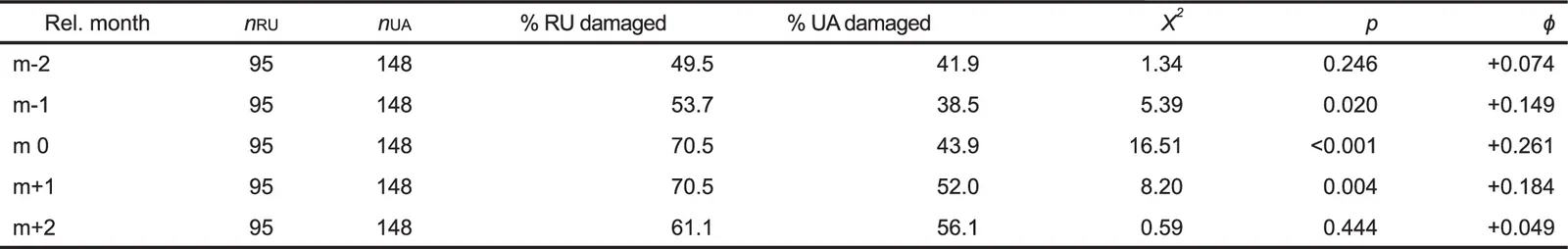
Statistical differences in the proportion of populated areas experiencing destruction between Russian and Ukrainian gains

Two-sided Pearson’s chi-squared tests on the proportion of populated areas with at least one newly damaged building. Positive effect sizes indicate a higher incidence of damage in Russian-gain cells. The asymmetry is statistically significant in the month before, of, and after the transition (${m}_{-1}$, ${m}_{0}$, ${m}_{+1}$), and largest at the month of the transition itself. Differences at ${m}_{-2}$ and ${m}_{+2}$ are not statistically significant.

**Extended Data Table 2:**
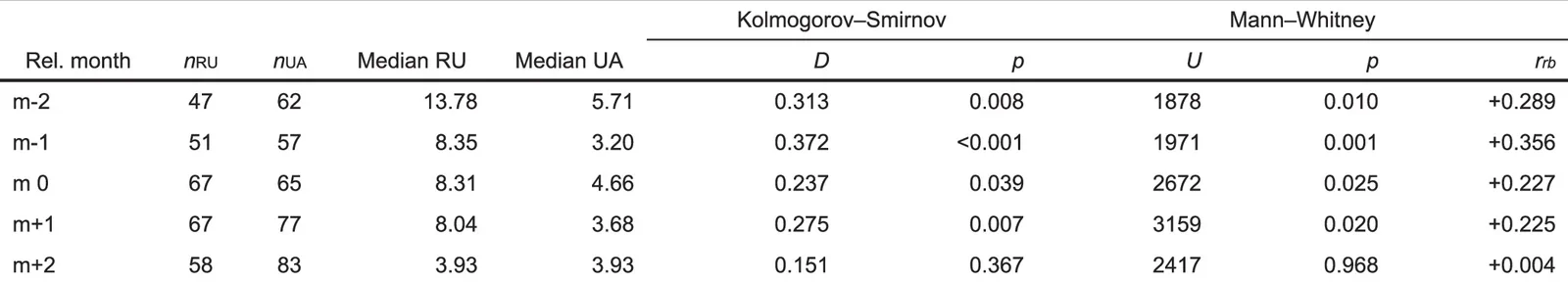
Statistical differences in levels of destruction between Russian and Ukrainian gains

Distribution of newly damaged buildings per 1,000 intact buildings, conditional on at least one newly damaged building in month m, using counts normalized per 1,000 buildings intact at the start of month m. We report the Kolmogorov–Smirnov D statistic with its p-value, and the Mann–Whitney U with rank-biserial correlation as a non-parametric cross-check. Positive effect sizes indicate that Russian-gain cells exceed Ukrainian-gain cells. The asymmetry is statistically significant from ${m}_{-2}$ through ${m}_{+1}$ under both tests, and converges by ${m}_{+2}$.

## Data Availability

All data necessary to reproduce results and figures are available at https://doi.org/10.5281/zenodo.21809900 (ref. ^81^).

## Code availability

All scripts and information necessary to reproduce results and figures are available at https://doi.org/10.5281/zenodo.21809900 (ref. ^81^).
