# Supplementary material for: Advancing conflict research and response through satellite-derived data
Source: Nature. 2026 Sep 9;657(8133):891–9. doi: 10.1038/s41586-026-11004-6 (PMC13600924; doi:10.1038/s41586-026-11004-6)
Supplement: Supplementary file 1 [file 41586_2026_11004_MOESM1_ESM.pdf]

nature portfolio

https://doi.org/10.1038/s41586-026-11004-6

Supplementary information

Advancing conflict research and response through satellite-derived data

In the format provided by the authors and unedited

Supplementary information

A Data on three aspects of violence

We can broadly distinguish three measurable aspects of conflict-related violence that help us study armed conflict. The first concerns the perpetration of violence and includes information on the actors and instruments used to conduct violence. Data on this aspect encompass information on the groups or individuals who carry out violent acts, such as combatants, and the means through which they do so, such as tanks or weapon systems.

The second aspect concerns the direct effects of violence. Conflict actors target military forces to degrade an opponent's capabilities and resolve. Civilians are often harmed as collateral damage and, increasingly, are deliberately targeted[1]—despite explicit prohibitions under international humanitarian law. Conflict actors also frequently attack material targets, producing physical destruction. They strike military assets such as armored personnel carriers, barracks, or tanks, and civilian infrastructure critical to military operations, like roads, bridges, or radio towers. As we observe across conflicts, armed groups also often target the foundations of civilian life, including homes[28], cultural and religious sites[82], and essential infrastructure such as hospitals and water facilities[83–86]. In contexts such as Syria, South Sudan, and Ethiopia, armed actors have extended violence to the natural environment, destroying crops and other resources vital for livelihoods[27,47,87,88].

The third aspect concerns the indirect consequences of violence. While some indicators of indirect consequences overlap with direct effects, they capture impacts that occur through more diffuse pathways. Armed conflict causes deaths not only through direct attack but also indirectly, for example when violence drives famine or prevents access to essential health services[43]. Similarly, while the built and natural environments may be directly destroyed, indirect consequences can arise from failures to maintain critical infrastructure or from reduced agricultural activity. Violence may also have indirect consequences for individuals who survive in conflict-affected environments including psychological issues or antisocial behavior. Data on this third aspect include measures such as indirect mortality, individuals' well-being, patterns of agricultural production, economic activity, and displacement camps.

To directly understand and respond to violence, data on the second aspect—ideally combined with information on perpetrators and instruments (the first aspect)—are most critical, as they reveal how violence unfolds over time and space. In our Analysis, we therefore focus on approaches that enable the collection of data on the second aspect—the direct effects of violence—while showing how combining these data with the first aspect can generate new insights. We acknowledge that the approaches presented in this Analysis are not equally suited to capture all forms of direct effects (for example, sexual violence). We also acknowledge that data on the third aspect are essential for understanding the long-term impacts of armed conflict and its intersections with issues such as development and health. We argue that data integration efforts similar to those described in the main article should be pursued to achieve a comprehensive understanding of the enduring consequences of violence.

B War damage and human toll of conflict

B.1 Approaches to capture war damage

As described in the main article, recent research has made significant progress in automatically capturing building damage from satellite imagery. Historically, automated damage assessment has focused primarily on destruction induced by natural disasters[89–93]. Adapting these methods to armed conflict presents unique challenges, as war-related damage is typically more spatially dispersed and spans longer timeframes. Consequently, systematic detection requires tools capable of repetitively screening large areas, while remaining accurate enough to isolate the few damaged structures in regions where the majority of the built environment remains intact[7,94]. These constraints render manual large-scale data collections infeasible and pose challenges for automated approaches.

Several recent studies address these challenges. Machine learning-based methods using very high-resolution (VHR) imagery can achieve high performance[7,49,50], but their broader applicability is constrained by high costs, demanding computing requirements, and often limited availability of VHR optical imagery[24] (see Fig. 1 in the main article). For large-scale data collection, approaches that use freely and globally available satellite data, currently limited to moderate resolution, are more realistic[8–12].

Approaches that rely on open optical images perform well in some contexts[8] but are restricted in areas prone to cloud coverage and mainly suited for detecting relatively large-scale damage. Other approaches employ Synthetic Aperture Radar (SAR) data, a fundamentally different form of satellite observations. Unlike optical sensors, SAR sensors emit microwave signals and capture their reflections from the surface. While SAR data are more difficult for human analysts to interpret than optical data, they offer distinct advantages, including penetrating cloud cover and operating independently of daylight. SAR-based change detection methods, notably those that leverage the phase of the SAR signal through SAR coherence or full interferometry (InSAR), are an established approach to detect damage following natural disasters[91,95–100]. Recent studies have begun to apply them successfully to conflict monitoring[9,11,12]. Other approaches rely on amplitude-only SAR time series, which are more lightweight to process[10].

A different approach uses fires detected through thermal imaging as indicators of possible war damage. Fires are prevalent across the spectrum of modern warfare, and fire data have supported assessments of dwelling destruction[101–104]. While many studies rely on manual interpretation validated with optical imagery, some use automated analysis[105,106]. Such approaches could potentially guide existing damage assessments to areas where damage likely occurred.

Satellite data also capture conflict-driven changes to the natural environment. Studies document vegetation damage from rocket fire or munitions detonations[48], fires set to trees and crops[26,51], and other forms of direct clearance of vegetation[52] that may be aimed at undermining food security[47,87], disrupting agricultural livelihoods[27,107], or removing cover for combatants[88]. Satellite observations also document the scale and speed of conflict-induced changes in reservoirs[108], waterways[109], and air quality[110], often linked to infrastructural damage[46] separately identified through image analysis. Indirect changes to soil[111], water quality[112], and broader land use patterns[44,113–115] during wartime are also readily detected; while sometimes unintended, they are no less detrimental to resource-dependent livelihoods and human health[116]. Long-term satellite monitoring further reveals the environmental

impacts of war preparation[117] and the enduring ecological legacies of war[45,118], which are not typically recorded in conflict event databases.

B.2 Capturing the human toll

Capturing violence against people through satellite data presents greater challenges, unless such violence results in material damage or other physical changes. Humanitarian actors use satellite imagery to identify new mass graves for operational needs[119]—information that can also serve as an indicator of conflict severity in the absence of reliable reporting. However, fatalities are usually better captured through text-based data.

One area where satellite-derived data can provide valuable insights is in identifying the place, time and potential scale of complex humanitarian emergencies that conflicts often trigger by disrupting essential services such as food, water, healthcare and mobility. Satellite data on nighttime light (NTL) can indirectly signal such disruptions to civilian life. Research shows that NTL fluctuations align spatially with shifting frontlines and temporally with escalations and lulls in violence[29,31,120–123]. This makes NTL a promising general proxy for humanitarian crises[124] while underscoring the need for more research to determine which phenomena it reliably captures.

The greatest potential of satellite-derived data for capturing the human toll of conflict, however, lies in capturing data on displacement, a key manifestation of conflict-related violence[125]. This is especially important because displacement is notoriously difficult to observe: traditional data collection approaches, often reliant on surveys or potentially biased administrative reporting, typically only yield infrequent, unsystematic information[126,127] at an aggregated level[128]. Such limitations hinder effective humanitarian response and obscure our understanding of displacement dynamics.

Satellite data cannot directly measure displacement, but can help us identify origins and destinations, estimate population sizes, and—in combination with other data—offer insights into population movements. Because dwellings and assets like crops are essential for shelter, livelihoods, and food security[52,87,129], georeferenced data on their destruction or abandonment can provide indirect cues about displacement origins[52,114,130], information that rarely exists[131]. One particular challenge is that large populations may be involuntarily immobile due to climate stress, poverty, legal barriers, or type of conflict[132], while others may flee in anticipation of violence even before damage occurs. Data on NTL[133–136] and potentially even on waste disposal sites[137] could complement displacement origin analysis by indicating whether humans still reside in an area. Similarly, satellite data can offer information on displacement destinations by detecting new structures. Satellite analysis has long been used to identify camps and informal settlements[138,139], with deep learning techniques using VHR imagery emerging more recently[140,141]. Beyond locating camps and estimating their sizes, studies using open-access data show that automatically detecting new settlement construction can help inform population count estimates[142–144].

C Data generation processes and limitations

Text-based data and satellite-derived data are collected through fundamentally different data generation processes (see Fig. 1), resulting in distinct strengths and core limitations.

C.1 Text-based data

Text-based data are currently the only type of conflict event data available in a systematic and global manner[14–16]. These datasets primarily draw on global and local media reporting[30] and provide coverage of violent events over multiple decades.

The dominant approach to collecting such data remains manual coding. Human coders generate conflict event data by triangulating newswire reports and incorporating other forms of human reporting, including information from non-governmental organizations, international organizations, and social media. The source material used for coding is selected through defined search strategies, such as systematic media monitoring using keywords, complemented by specialized sources and targeted additional searches[145]. Manual coding currently remains the benchmark for data quality, as trained coders cross-check sources and verify reported information. At the same time, manual coding faces inherent challenges. It is highly labor intensive and constrained in its coverage of languages[4]. Coders also frequently confront a sourcing dilemma[18]: proximity to an event tends to increase the level of detail available, but also raises the risk of bias.

With advances in natural language processing and large language models, automated approaches to detecting conflict events from text are beginning to emerge[31–33]. A key advantage of these approaches is scalability: once established, they require substantially less human labor and can process large volumes of text. Automated methods may also mitigate some limitations of manual coding, for example by incorporating local news sources in local languages, thereby increasing coverage of conflicts that receive limited international media attention, although the sourcing dilemma persists. At the same time, automation introduces new challenges, particularly due to reduced corroboration and validation of events. Partisan reporting, misinformation, and disinformation pose serious problems for text-based data collection[4]. While professional data collection efforts mitigate these risks through qualitative source assessment and triangulation, automated approaches remain more vulnerable to biased or misleading inputs. Existing methods also tend to rely on relatively simple procedures for de-duplication and event consolidation and involve trade-offs between performance and precision, with some uncertainty likely to persist even as detection methods continue to improve.

Improvements in neither manual nor automated approaches can resolve the core challenges associated with reliance on human reporting. These challenges stem from the underlying data sources themselves rather than from specific collection or coding procedures. As a result, they affect both the likelihood that violence is recorded in a dataset and the accuracy with which it is recorded. Three notable examples are:

Uneven coverage: the probability that conflict events are documented in text-based data varies widely across space and contexts. It depends on factors such as proximity to population centers, access to communication infrastructure, and the ability of media or other observers to reach affected areas[4–6,35,146]. These dynamics generate systematic gaps in coverage and a strong urban bias, both in terms of whether events are recorded at all and the spatial and temporal precision with which they are captured.

Newsworthiness: although text-based datasets can, in principle, record any aspect of political violence that is reported in their source material, some types of events are far more likely to be documented than others. Events involving fatalities are far more likely to be covered in news and other forms of human reporting than non-lethal events[14,17]. Consequently, widely used conflict datasets tend to focus on specific forms of violence, such as

events involving fatalities among combatants and civilians[14,147], or on other salient categories of political violence, including battles, violence against civilians, protests, explosions, and strategic developments[15]. Non-lethal forms of violence are strongly underrepresented in conflict event data[17]. Furthermore, events that involve large groups of victims are more likely to be reported than small events[148].

Spatial imprecision: text-based data can only approximate the location of violent events. While some reporting provides precise spatial information, in many cases it is not possible to identify the exact location where violence occurred[5,6]. In the most widely used datasets[14,15], the highest level of spatial precision is usually at the settlement, represented by a coordinate (e.g., the settlement centroid). Frequently, even this level of detail is unavailable, and coders assign locations at the level of districts, provinces, or entire countries. Data providers are transparent about these procedures and include information on spatial precision to reduce the risk of misleading inference. Nonetheless, spatial imprecision has important consequences for researchers, often constraining them to conduct analyses at more aggregate spatial levels when relying on existing conflict event data[6].

C.2 Satellite-derived data

In contrast to text-based conflict event data, satellite-derived data on violence are not yet available on a systematic and global basis. Historically, the dominant approach to collecting such data was the visual inspection of VHR images, but manual annotation is highly labor-intensive and VHR imagery is usually only available through expensive commercial providers. Because large-scale collection requires repeated screening of entire conflict zones, such resource-heavy and costly approaches are often not feasible. Emerging automated detection methods open the door to more systematic coverage. As noted in Appendix B, approaches to automatically detect damage are available using both VHR and moderate resolution imagery. At scale, however, open-access data are often the only option,[d] even if their currently lower spatial resolution makes detection more challenging.

Several factors shape how reliably violence can be detected in satellite data, including the form of violence, the physical environment, the satellite mission and sensor modality used, and researcher choices around where to train models.

Form of violence: Buildings that are torched, damaged by an airdropped bomb, or demolished by an armored bulldozer are more readily detected than those that sustain lateral damage from high-precision targeted strikes or small arms and light weapons. Some forms of violence leave relatively little impact on the built-up or natural environment. Cartel and gang violence in Mexico and Central America, for example, produces mass fatalities, but material destruction is sporadic and localized. Because the spatial and temporal co-occurrence of war damage and other manifestations of violence, such as fatalities, varies across conflict contexts and actors, researchers should use satellite-derived war damage data as a proxy for the intensity of violence only where strong assumptions about co-occurrence hold. Integrating satellite-derived and text-based data likewise requires a clear account of how different manifestations of violence relate in the specific case at hand.

Physical environment: Detectability varies across conflict settings and within them. Dense vegetation hides underlying structures; in heavily forested conflict settings such as Myanmar, Colombia, or the Democratic Republic of Congo, tree canopy makes it difficult to identify

[d] Unless commercial providers agree to support conflict research with access to imagery, and possibly also compute resources.

damaged buildings, unless the vegetation is burned along with the dwellings. Even within a single conflict zone, detectability may vary between urban centers and peripheral areas. Buildings in the periphery tend to be smaller and more dispersed. Furthermore, the use of organic construction materials, especially in comparatively less developed rural areas, can reduce the visibility of structures. For example, the use of clay and wood for walls and building foundations, along with thatched roofs made out of straw, grass, and similar materials in the context of Cameroon's Far North Region means that rural dwellings often have similar reflection signatures as the surrounding landscape. Smaller and more informal dwellings are also less reliably captured by the settlement and building layers. If such layers are used in post-processing, this can become an additional source of error both across geographic contexts and within individual countries. Some challenges related to the physical environment are more salient for moderate-resolution imagery than for VHR imagery: for example, damage to small structures is more challenging to detect in moderate resolution imagery as the structure may only occupy one pixel; accordingly, the urban bias is likely more pronounced for assessments based on moderate resolution data. As sensors improve and settlement and building layers are updated more regularly, such biases can be mitigated.

Satellite mission and sensor modality: Identifying the timing of physical damage or new structures typically requires at least two images to detect change. The archival availability of commercial satellite data varies by constellation. Open-access satellite data, such as those provided by NASA and ESA, have high revisit frequencies but are currently available only at medium to low spatial resolution. Cloud cover and other atmospheric issues may extend intervals between usable optical images to weeks or months, introducing seasonal patterns and disparities between cloud-prone regions, such as the Congo Basin, and arid regions, such as the Sahel. SAR-based approaches (see Appendix B) can mitigate cloud-cover constraints, although validation through very high-resolution optical imagery may remain challenging. Different satellite products also have varying levels of positional error, so simply overlaying data from different sources can produce highly uncertain results, requiring careful accounting for these errors in analysis.

Researcher choices and data availability: Beyond technical processing, the likelihood of detecting specific violence manifestations is also shaped by choices made by researchers who develop automated detection methods. Examples of these choices include decisions about the conflicts or geographical areas used to train statistical models, or the types of phenomena to investigate (e.g., urban damage versus cropland abandonment). Such decisions inherently influence which manifestations of political violence, and in which locations, are more or less likely to be detected and, consequently, their inclusion in satellite-derived datasets. Current approaches are typically trained in settings where reference data is available, such as Ukraine, Gaza, and Syria, and may not transfer easily to other settings, in particular to settings with other types of violence and less formal settlements. However, this problem can be mitigated by aligning research priorities with the goal of systematic large-scale data collections, including prioritizing transferability and generalization.

Yet ultimately, while technological advances offer potential improvement and mitigate some challenges, the core limitations related to satellite-derived data persist. The data remain limited to objects or impacts visible from specific satellite viewing angles and are restricted to events with physical manifestations. And while satellite imagery provides essential large-scale geographic context often not available through text-based data, it lacks other forms of contextual information that can only be obtained from textual sources.

D Automated spatial improvement

While the local-level spatial improvement analyses presented in the main article (Fig. 2 and Extended Data Figs. 1 and 2) were implemented manually to illustrate the analytical logic, the underlying workflow is fully scalable, contingent on the availability of satellite-derived damage data. In contexts where settlement polygons are available, such as the GRID3 settlement extents datasets for Sub-Saharan Africa[149], the procedure can be implemented systematically. In such settings, longitude and latitude information from conflict event data can be intersected with settlement polygons to identify affected settlements, which can then be spatially intersected with satellite-derived damage data to delineate destroyed and non-destroyed areas within each settlement.

For larger-scale analyses where visual inspection of individual settlements is not feasible, this pattern can be quantified using spatial indicators, such as the total area or proportion of the settlement affected by destruction. To distinguish between localized and dispersed targeting patterns, researchers can employ measures of damage concentration (such as the share of total destroyed area accounted for by the largest contiguous damage polygons or the density of damage clusters within a settlement). If detailed within-settlement spatial data on the spatial distribution of ethnic residents are available, this information can also be mapped onto the destroyed versus intact area information derived from the satellite imagery to identify correlations between ethnicity and destruction patterns.

In contexts where settlement polygons are unavailable, as in our Myanmar analysis, the same logic can be adapted using the smallest available administrative units to pre-filter affected versus non-affected areas using the conflict event data. These areas can subsequently be intersected with high-resolution population data to isolate inhabited space, which is then overlaid with satellite-derived damage data to classify populated areas as affected and non-affected. Summary indicators capturing the extent and spatial distribution of destruction can be derived from these data, enabling comparative analyses across locations.

E Spatial-autocorrelation analysis

In the main article, we assess differences in building damage between populated areas gained by Russia and those retaken by Ukraine, conducting statistical analyses within a five-month window of first stable transitions during the frontline war (results reported in Fig. 3 and Extended Data Tables 1 and 2). To check the independence assumption underlying the per-month tests, we compute Moran's I on $\log 1p$(newly damaged buildings) using a spatio-temporal weights matrix (see Table E1).

Moran's I is positive and statistically significant across all relative months, indicating spatial clustering of damage. However, values are essentially flat between $T = 0$ and $T = 2$, indicating that event timing is not driving the spatial pattern. We interpret this as evidence that the clustering reflects timing-invariant baseline structure such as frontline geography and terrain, rather than within-cohort spillover between contemporaneously affected cells. The per-relative-month tests are therefore informative about the asymmetry in building damage associated with Russian versus Ukrainian gains, not artefacts of localized contagion. Note that these tests describe the asymmetry observed in our sample of first stable transitions; they do not isolate targeting choices from operational differences between Russian advances and Ukrainian counteroffensives.

T	Group	Rel. month	n	n_{active}	I	z	P_{perm}
	Pooled	m-2	243	241	+0.405	11.44	0.001
	Pooled	m-1	243	241	+0.373	10.81	0.001
	Pooled	m 0	243	241	+0.247	7.42	0.001
	Pooled	m+1	243	241	+0.253	7.24	0.001
	Pooled	m+2	243	241	+0.197	5.82	0.001
	Russian gains	m-2	95	93	+0.453	6.48	0.001
	Russian gains	m-1	95	93	+0.419	6.40	0.001
0	Russian gains	m 0	95	93	+0.241	3.62	0.001
	Russian gains	m+1	95	93	+0.254	3.89	0.002
	Russian gains	m+2	95	93	+0.133	2.00	0.050
	Ukrainian gains	m-2	148	145	+0.287	6.82	0.001
	Ukrainian gains	m-1	148	145	+0.175	3.80	0.003
	Ukrainian gains	m 0	148	145	+0.149	3.51	0.004
	Ukrainian gains	m+1	148	145	+0.240	5.46	0.001
	Ukrainian gains	m+2	148	145	+0.281	6.29	0.001
	Pooled	m-2	243	243	+0.412	12.02	0.001
	Pooled	m-1	243	243	+0.365	11.06	0.001
	Pooled	m 0	243	243	+0.263	7.72	0.001
	Pooled	m+1	243	243	+0.247	7.11	0.001
	Pooled	m+2	243	243	+0.212	6.16	0.001
	Russian gains	m-2	95	95	+0.460	8.33	0.001
	Russian gains	m-1	95	95	+0.364	6.94	0.001
2	Russian gains	m 0	95	95	+0.270	5.16	0.001
	Russian gains	m+1	95	95	+0.194	3.81	0.001
	Russian gains	m+2	95	95	+0.115	2.18	0.034
	Ukrainian gains	m-2	148	148	+0.295	6.75	0.001
	Ukrainian gains	m-1	148	148	+0.206	4.84	0.001
	Ukrainian gains	m 0	148	148	+0.146	3.50	0.005
	Ukrainian gains	m+1	148	148	+0.247	5.75	0.001
	Ukrainian gains	m+2	148	148	+0.274	6.48	0.001

Table E1: **Moran's I on log1p(newly damaged buildings) using a spatio-temporal weights matrix.** For each populated area, neighbors are defined as the six nearest cells by centroid distance, restricted to those whose event month falls within $\pm T$ months of the target. We report results at $T = 0$ (strict same-event-month neighbours) and $T = 2$ (± 2 months), with 999-permutation p-values.

References

[1] Shawn Davies, Therése Pettersson, Margareta Sollenberg, and Magnus Öberg. "Organized violence 1989–2024, and the challenges of identifying civilian victims". In: *Journal of Peace Research* 62.4 (2025), pp. 1223–1240.

[4] Erin Miller, Roudabeh Kishi, Clionadh Raleigh, and Caitriona Dowd. "An agenda for addressing bias in conflict data". In: *Scientific Data* 9.1 (2022), p. 593.

[5] Nils B. Weidmann. "A Closer Look at Reporting Bias in Conflict Event Data". In: *American Journal of Political Science* 60.1 (2016), pp. 206–218.

[6] Nils B. Weidmann. "On the Accuracy of Media-based Conflict Event Data". In: *Journal of Conflict Resolution* 59.6 (2015), pp. 1129–1149.

[7] Hannes Mueller, Andre Groeger, Jonathan Hersh, Andrea Matranga, and Joan Serrat. "Monitoring war destruction from space using machine learning". In: *Proceedings of the National Academy of Sciences* 118.23 (2021), e2025400118.

[8] Zhengyang Hou et al. "War city profiles drawn from satellite images". In: *Nature Cities* 1 (2024), pp. 359–369.

[9] Corey Scher and Jamon Van Den Hoek. "Nationwide conflict damage mapping with interferometric synthetic aperture radar: A study of the 2022 Russia-Ukraine conflict". In: *Science of Remote Sensing* (2025), p. 100217.

[10] Olivier Dietrich et al. "An open-source tool for mapping war destruction at scale in Ukraine using Sentinel-1 time series". In: *Communications Earth & Environment* 6.1 (2025), p. 215.

[11] Daniel Racek, Qi Zhang, Paul Wilhelm Thurner, Xiao Xiang Zhu, and Göran Kauermann. "Unsupervised detection of building destruction during war from publicly available radar satellite imagery". In: *PNAS Nexus* 4.12 (2025), pgaf367.

[12] Corey Scher and Jamon Van Den Hoek. *Active InSAR monitoring of building damage in Gaza during the Israel-Hamas War*. arXiv preprint. https://arxiv.org/abs/2506.14730. 2025.

[14] Ralph Sundberg and Erik Melander. "Introducing the UCDP Georeferenced Event Dataset". In: *Journal of Peace Research* 50.4 (2013), pp. 523–532.

[15] Clionadh Raleigh, Roudabeh Kishi, and Andrew Linke. "Political instability patterns are obscured by conflict dataset scope conditions, sources, and coding choices". In: *Humanities and Social Sciences Communications* 10.1 (2023), p. 74.

[16] Gary LaFree and Laura Dugan. "Introducing the global terrorism database". In: *Terrorism and Political Violence* 19.2 (2007), pp. 181–204.

[17] Mihai Croicu and Kristine Eck. "Reporting of non-fatal conflict events". In: *International Interactions* 48.3 (2022), pp. 450–470.

[18] Magnus Öberg and Mert Can Yilmaz. "Measurement issues in conflict event data: Addressing some misconceptions about what drives differences between human-coded event datasets". In: *Research & Politics* 12.3 (2025), p. 20531680251362440.

[24] Valerie Sticher, Jan D Wegner, and Birke Pfeifle. "Toward the Remote Monitoring of Armed Conflicts". In: *PNAS Nexus* (2023), pgad181.

[26] Hadi Jaafar, Lara Sujud, and Eckart Woertz. "Scorched earth tactics of the "Islamic State" after its loss of territory: intentional burning of farmland in Iraq and Syria". In: *Regional Environmental Change* 22.4 (2022), p. 120.

[27] Nataliia Kussul et al. "Assessment of war-induced agricultural land use changes in Ukraine using machine learning applied to Sentinel satellite data". In: *International Journal of Applied Earth Observation and Geoinformation* 140 (2025), p. 104551.

[28] Yara Asi et al. "'Nowhere and no one is safe': spatial analysis of damage to critical civilian infrastructure in the Gaza Strip during the first phase of the Israeli military campaign, 7 October to 22 November 2023". In: *Conflict and Health* 18.1 (2024), p. 24.

[29] Yuehan Yu, Shirao Liu, Yuanqing Li, and Kaifang Shi. "Satellite remotely sensed nighttime lights reveal spatiotemporal dynamics of the Ukrainian-Russian conflict". In: *IEEE Geoscience and Remote Sensing Letters* 20 (2023), pp. 1–5.

[30] Mihai Croicu. "Forecasting battles: New machine learning methods for predicting armed conflict". PhD thesis. Uppsala, Sweden: Uppsala University, 2025.

[31] Yuri Zhukov. "Near-real time analysis of war and economic activity during Russia's invasion of Ukraine". In: *Journal of Comparative Economics* (2023), pp. 1232–1243.

[32] Laura Braun and Christian Oswald. *Automated information extraction from text variables in event datasets with large language models.* OSF Preprints. https://osf.io/yxp8k$_v$2. 2025.

[33] Étienne Simon et al. "Abstractive Event Analysis of Armed Conflicts: Introducing the UCDP-AEC Dataset". In: *Proceedings of the 21st Conference on Natural Language Processing (KONVENS 2025): Workshops.* Ed. by Christian Wartena and Ulrich Heid. Hannover, Germany: HsH Applied Academics, 2025, pp. 104–119.

[35] Kristine Eck. "In data we trust? A comparison of UCDP GED and ACLED conflict events datasets". In: *Cooperation and Conflict* 47.1 (2012), pp. 124–141.

[43] Hazem Adam Ghobarah, Paul Huth, and Bruce Russett. "Civil wars kill and maim people—long after the shooting stops". In: *American Political Science Review* 97.2 (2003), pp. 189–202.

[44] Emnet Negash et al. "Remote sensing reveals how armed conflict regressed woody vegetation cover and ecosystem restoration efforts in Tigray (Ethiopia)". In: *Science of Remote Sensing* 8 (2023), p. 100108.

[45] Florian Krampe, Joakim Kreutz, and Tobias Ide. "Armed conflict causes long-lasting environmental harms". In: *Environment and Security* (2025).

[46] Erika Weinthal and Jeannie Sowers. "Targeting infrastructure and livelihoods in the West Bank and Gaza". In: *International Affairs* 95.2 (2019), pp. 319–340.

[47] Anna Feuer. "Environmental warfare tactics in irregular conflicts". In: *Perspectives on Politics* 21.2 (2023), pp. 533–549.

[48] Mikael Hiberg Naghizadeh. "The uses for fire data and satellite images in monitoring, detecting, and documenting collective political violence". In: *Research & Politics* 11.3 (2024).

[49] Erik C Duncan, Sergii Skakun, Ankit Kariryaa, and Alexander V Prishchepov. "Detection and mapping of artillery craters with very high spatial resolution satellite imagery and deep learning". In: *Science of Remote Sensing* 7 (2023), p. 100092.

[50] Bakhtiar Feizizadeh et al. "An integrated object-based-deep learning approach applied for mapping armed conflict impacts and land scars". In: *Science of Remote Sensing* (2025), p. 100257.

[51] Lina Eklund and Pinar Dinc. "Fires as collateral or means of war: challenges of environmental peacebuilding in the Kurdistan Region of Iraq". In: *Ecology and Society* 29.3 (2024), art25.

[52] He Yin, Lina Eklund, Dimah Habash, Mazin B. Qumsiyeh, and Jamon Van Den Hoek. "Evaluating war-induced damage to agricultural land in the Gaza Strip since October 2023 using PlanetScope and SkySat imagery". In: *Science of Remote Sensing* 11 (2025), p. 100199.

[82] Luis Felipe Mantilla and Zorana Knezevic. "Explaining intentional cultural destruction in the Syrian Civil War". In: *Journal of Peace Research* 59.4 (2022), pp. 562–576.
[83] Melanie Sauter and Scott Gates. *Attacks on healthcare in Ukraine: Evidence from Geospatial Analysis*. OSF Preprints. Preprint, version 1. 2025.
[84] Evan Muzzall, Brian Perlman, Leonard S Rubenstein, and Rohini J Haar. "Overview of attacks against civilian infrastructure during the Syrian civil war, 2012–2018". In: *BMJ Global Health* 6.10 (2021), e006384.
[85] Jeannie Sowers, Erika Weinthal, and Neda Zawahri. "Targeting environmental infrastructures, international law, and civilians in the new Middle Eastern wars". In: *Security Dialogue* 48.5 (2017), pp. 410–430.
[86] Brian Perlman, Shalean M. Collins, and Jamon Van Den Hoek. "Public health implications of satellite-detected widespread damage to WASH infrastructure in the Gaza Strip". In: *PLOS Global Public Health* 5.2 (2025). Ed. by Nancy Angeline Gnanaselvam, e0004221.
[87] Victor Mackenhauer Olsen et al. "The impact of conflict-driven cropland abandonment on food insecurity in South Sudan revealed using satellite remote sensing". In: *Nature Food* 2.12 (2021), pp. 990–996.
[88] Paulo J. Murillo-Sandoval, Kristina Van Dexter, Jamon Van Den Hoek, David Wrathall, and Robert Kennedy. "The end of gunpoint conservation: forest disturbance after the Colombian peace agreement". In: *Environmental Research Letters* 15.3 (2020), p. 034033.
[89] Ritwik Gupta et al. "Creating xBD: A dataset for assessing building damage from satellite imagery". In: *Proceedings of the IEEE/CVF conference on computer vision and pattern recognition workshops*. 2019, pp. 10–17.
[90] Jihyeon Lee et al. *Assessing post-disaster damage from satellite imagery using semi-supervised learning techniques*. arXiv preprint. arXiv:2011.14004. 2020.
[91] Cheryl W. J. Tay et al. "Rapid flood and damage mapping using synthetic aperture radar in response to Typhoon Hagibis, Japan". In: *Scientific Data* 7.1 (2020).
[92] Shahrzad Gholami et al. "On the Deployment of Post-Disaster Building Damage Assessment Tools using Satellite Imagery: A Deep Learning Approach". In: *2022 IEEE International Conference on Data Mining Workshops (ICDMW)*. IEEE, 2022, pp. 1029–1036.
[93] Akhyar Akhyar et al. "Deep artificial intelligence applications for natural disaster management systems: A methodological review". In: *Ecological Indicators* 163 (2024), p. 112067.
[94] Jamon Van Den Hoek. "The City is the Medium and Satellite Imagery Are a Prism". In: *Urban Remote Sensing*. John Wiley Sons, Ltd, 2021. Chap. 15, pp. 325–333.
[95] Eric J Fielding et al. "Surface ruptures and building damage of the 2003 Bam, Iran, earthquake mapped by satellite synthetic aperture radar interferometric correlation". In: *Journal of Geophysical Research: Solid Earth* 110.B3 (2005).
[96] Simon Plank. "Rapid damage assessment by means of multi-temporal SAR—A comprehensive review and outlook to Sentinel-1". In: *Remote Sensing* 6.6 (2014), pp. 4870–4906.
[97] Sang-Ho Yun et al. "Rapid Damage Mapping for the 2015Mw 7.8 Gorkha Earthquake Using Synthetic Aperture Radar Data from COSMO–SkyMed and ALOS-2 Satellites". In: *Seismological Research Letters* 86.6 (2015), pp. 1549–1556.
[98] Jungkyo Jung, Duk-jin Kim, Marco Lavalle, and Sang-Ho Yun. "Coherent Change Detection Using InSAR Temporal Decorrelation Model: A Case Study for Volcanic

Ash Detection". In: *IEEE Transactions on Geoscience and Remote Sensing* 54.10 (2016), pp. 5765–5775.

[99] Jungkyo Jung, Sang-Ho Yun, Duk-jin Kim, and Marco Lavalle. "Damage-Mapping Algorithm Based on Coherence Model Using Multitemporal Polarimetric–Interferometric SAR Data". In: *IEEE Transactions on Geoscience and Remote Sensing* 56.3 (2018), pp. 1520–1532.

[100] Eric Jameson Fielding and Jungkyo Jung. "Damage Proxy Mapping with SAR interferometric coherence change". In: *Procedia Computer Science* 239 (2024), pp. 2322–2328.

[101] Lars Bromley. "Relating violence to MODIS fire detections in Darfur, Sudan". In: *International Journal of Remote Sensing* 31.9 (2010), pp. 2277–2292.

[102] Mikael Hiberg Naghizadeh. "What we don't see: Uncovering intercommunal violence through remote sensing". In: *Political Geography* 116 (2025), p. 103259.

[103] *Myanmar: Video and Satellite Evidence Shows New Fires Still Torching Rohingya Villages.* Amnesty International, 2017.

[104] *Burma: Military Torches homes near border.* Dhaka, Bangladesh: Human Rights Watch, 2017.

[105] Andrew Marx, Richard Windisch, and Jong Su Kim. "Detecting village burnings with high-cadence smallsats: A case-study in the Rakhine State of Myanmar". In: *Remote Sensing Applications: Society and Environment* 14 (2019), pp. 119–125.

[106] The Economist and Sondre Solstad. "The Economist war-fire model. First published in the article "A hail of destruction"". In: *The Economist* (2023).

[107] Liya Weldegebriel, Emnet Negash, Jan Nyssen, and David B Lobell. "Eyes in the sky on Tigray, Ethiopia-Monitoring the impact of armed conflict on cultivated highlands using satellite imagery". In: *Science of Remote Sensing* 9 (2024), p. 100133.

[108] O. Shumilova et al. "Environmental effects of the Kakhovka Dam destruction by warfare in Ukraine". In: *Science* 387.6739 (2025), pp. 1181–1186.

[109] Marc François Müller, Jim Yoon, Steven M. Gorelick, Nicolas Avisse, and Amaury Tilmant. "Impact of the Syrian refugee crisis on land use and transboundary freshwater resources". In: *Proceedings of the National Academy of Sciences* 113.52 (2016), pp. 14932–14937.

[110] S. S. Limaye, V. E. Suomi, C. Velden, and G. Tripoli. "Satellite Observations of Smoke from Oil Fires in Kuwait". In: *Science* 252.5012 (1991), pp. 1536–1539.

[111] Hailemariam Meaza et al. "The effects of armed conflict on natural resources and conservation measures in Tigray, Northern Ethiopia". In: *International Soil and Water Conservation Research* 13.2 (2025), pp. 463–474.

[112] Peter H. Gleick. "Water as a weapon and casualty of armed conflict: A review of recent water-related violence in Iraq, Syria, and Yemen". In: *WIREs Water* 6.4 (2019), e1351.

[113] Lina Eklund, Michael Degerald, Martin Brandt, Alexander V Prishchepov, and Petter Pilesjö. "How conflict affects land use: agricultural activity in areas seized by the Islamic State". In: *Environmental Research Letters* 12.5 (2017), p. 054004.

[114] Xi-Ya Li et al. "Civil war hinders crop production and threatens food security in Syria". In: *Nature Food* 3.1 (2022), pp. 38–46.

[115] Hannah R Kerner et al. "Satellite data shows resilience of Tigrayan farmers in crop cultivation during civil war". In: *Science of Remote Sensing* 10 (2024), p. 100140.

[116] Hailemariam Meaza et al. "Managing the environmental impacts of war: What can be learned from conflict-vulnerable communities?" In: *Science of The Total Environment* 927 (2024), p. 171974.

[117] Gary E. Machlis and Thor Hanson. “Warfare Ecology”. In: *BioScience* 58.8 (2008), pp. 729–736.
[118] Doug Weir et al. “Conservation policies must address an overlooked issue: how war affects the environment”. In: *Nature* 634.8034 (2024), pp. 538–541.
[119] Emilio Guida. *The Use of Satellites in Humanitarian Contexts*. NCHS Paper. Norwegian Centre for Humanitarian Studies, 2021.
[120] Yi Lin et al. “Pixel-Level Quantification of Damage and Recovery Caused by the Russia-Ukraine Conflict Based on Nighttime Light Imagery”. In: *IEEE Journal of Selected Topics in Applied Earth Observations and Remote Sensing* (2024).
[121] Xi Li, Shanshan Liu, Michael Jendryke, Deren Li, and Chuanqing Wu. “Night-Time Light Dynamics during the Iraqi Civil War”. In: *Remote Sensing* 10.6 (2018), p. 858.
[122] Zihao Zheng et al. “Estimates of power shortages and affected populations during the initial period of the Ukrainian-Russian conflict”. In: *Remote Sensing* 14.19 (2022), p. 4793.
[123] Wei Jiang, Guojin He, Tengfei Long, and Huichan Liu. “Ongoing Conflict Makes Yemen Dark: From the Perspective of Nighttime Light”. In: *Remote Sensing* 9.8 (2017), p. 798.
[124] Corinne Bara and Valerie Sticher. “The rural limits of conflict monitoring using nighttime lights”. In: *Humanities and Social Sciences Communications* 12.1 (2025), p. 905.
[125] Adam Lichtenheld. *Guilt by Location: Forced Displacement and Population Sorting in Civil Wars*. 1st ed. Cambridge University Press, 2024.
[126] Shiyu Wang and Xi Li. “Use of Nighttime Light Images in Evaluating Refugee Settlement”. In: *IEEE Journal of Selected Topics in Applied Earth Observations and Remote Sensing* 17 (2024), pp. 14122–14132.
[127] Xiao Hui Tai, Shikhar Mehra, and Joshua E Blumenstock. “Mobile phone data reveal the effects of violence on internal displacement in Afghanistan”. In: *Nature Human Behaviour* 6.5 (2022), pp. 624–634.
[128] Lisa Thalheimer and Woi Sok Oh. “An inventory tool to assess displacement data in the context of weather and climate-related events”. In: *Climate Risk Management* 40 (2023), p. 100509.
[129] Saeed Mhanna, Landon JS Halloran, François Zwahlen, Ahmed Haj Asaad, and Philip Brunner. “Using machine learning and remote sensing to track land use/land cover changes due to armed conflict”. In: *Science of the Total Environment* 898 (2023), p. 165600.
[130] Klaus Deininger et al. “Quantifying war-induced crop losses in Ukraine in near real time to strengthen local and global food security”. In: *Food Policy* 115 (2023), p. 102418.
[131] M. Enenkel et al. “Emergencies do not stop at night: Advanced analysis of displacement based on satellite-derived nighttime light observations”. In: *IBM Journal of Research and Development* 64.1/2 (2020), 8:1–8:12.
[132] Lisa Thalheimer et al. “Prioritizing involuntary immobility in climate policy and disaster planning”. In: *Nature Communications* 16.1 (2025), p. 2581.
[133] Nita Bharti, Xin Lu, Linus Bengtsson, Erik Wetter, and Andrew J. Tatem. “Remotely measuring populations during a crisis by overlaying two data sources”. In: *International Health* 7.2 (2015), pp. 90–98.
[134] Chaoqing Huang et al. “Mapping of nighttime light trends and refugee population changes in Ukraine during the Russian–Ukrainian War”. In: *Frontiers in Environmental Science* 11 (2023). Publisher: Frontiers.

[135] Xi Li and Deren Li. “Can night-time light images play a role in evaluating the Syrian Crisis?” In: *International Journal of Remote Sensing* 35.18 (2014), pp. 6648–6661.
[136] Frank D. W. Witmer and John O’Loughlin. “Detecting the Effects of Wars in the Caucasus Regions of Russia and Georgia Using Radiometrically Normalized DMSP-OLS Nighttime Lights Imagery”. In: *GIScience & Remote Sensing* 48.4 (2011), pp. 478–500.
[137] Fadwa Hodali, Krishna Karra, Caroline Alexander, and Denise Lu. “An Environmental Crisis Haunts the Ruins of Gaza”. In: *Bloomberg* (2025).
[138] Kristin Spröhnle et al. “Earth Observation-Based Dwelling Detection Approaches in a Highly Complex Refugee Camp Environment — A Comparative Study”. In: *Remote Sensing* 6.10 (2014), pp. 9277–9297.
[139] Kristin Spröhnle, Eva-Maria Fuchs, and Patrick Aravena Pelizari. “Object-Based Analysis and Fusion of Optical and SAR Satellite Data for Dwelling Detection in Refugee Camps”. In: *IEEE Journal of Selected Topics in Applied Earth Observations and Remote Sensing* 10.5 (2017), pp. 1780–1791.
[140] Getachew Workineh Gella et al. “Mapping of dwellings in IDP/refugee settlements from very high-resolution satellite imagery using a mask region-based convolutional neural network”. In: *Remote Sensing* 14.3 (2022), p. 689.
[141] Omid Ghorbanzadeh, Alessandro Crivellari, Dirk Tiede, Pedram Ghamisi, and Stefan Lang. “Mapping Dwellings in IDP/Refugee Settlements Using Deep Learning”. In: *Remote Sensing* 14.24 (2022), p. 6382.
[142] Nando Metzger et al. “Fine-grained population mapping from coarse census counts and open geodata”. In: *Scientific Reports* 12.1 (2022), p. 20085.
[143] Nando Metzger, Rodrigo Caye Daudt, Devis Tuia, and Konrad Schindler. “High-resolution population maps derived from Sentinel-1 and Sentinel-2”. In: *Remote Sensing of Environment* 314 (2024), p. 114383.
[144] Hannah K. Friedrich and Jamon Van Den Hoek. “Breaking ground: Automated disturbance detection with Landsat time series captures rapid refugee settlement establishment and growth in North Uganda”. In: *Computers, Environment and Urban Systems* 82 (2020), p. 101499.
[145] Stina Högbladh. *UCDP Georeferenced Event Dataset Codebook Version 24.1.* Uppsala Conflict Data Program, Uppsala University, 2024.
[146] Matthew A Baum and Yuri Zhukov. “Filtering revolution: Reporting bias in international newspaper coverage of the Libyan civil war”. In: *Journal of Peace Research* 52.3 (2015), pp. 384–400.
[147] Shawn Davies, Therése Pettersson, and Magnus Öberg. “Organized violence 1989–2022, and the return of conflict between states”. In: *Journal of Peace Research* 60.4 (2023), pp. 691–708.
[148] Megan Price and Patrick Ball. “Selection bias and the statistical patterns of mortality in conflict”. In: *Statistical Journal of the IAOS* 31.2 (2015), pp. 263–272.
[149] Center for International Earth Science Information Network. *GRID3 Settlement Extents.* 2026.
