# Supplementary material for: Advancing conflict research and response through satellite-derived data
Source: Nature. 2026 Sep 9;657(8133):891–9. doi: 10.1038/s41586-026-11004-6 (PMC13600924; doi:10.1038/s41586-026-11004-6)
Supplement: Supplementary file 3 [file 41586_2026_11004_MOESM3_ESM.pdf]

nature portfolio

Peer Review File

Advancing conflict research and response through satellite-derived data

Corresponding Author: Dr Valerie Sticher, Dr Corinne Bara

Any redactions in this file are there to maintain patient confidentiality, the confidentiality of unpublished data, or to remove third-party material.

This file contains all reviewer reports in order by version, followed by all author rebuttals in order by version.

Attachments originally included by the reviewers as part of their assessment can be found at the end of this file.

Version 1:

Reviewer comments:

Referee #1

(Remarks to the Author)
I am writing to provide a review for the article "Advancing conflict research and response through satellite-derived data". This perspective article has the goal to outline how satellite-driven data can help conflict research advance from where it is now. The authors make the point that, up-until now, text-based measurements of conflict have dominated research which comes with a set of specific issues. The article argues that satellite data can help mitigate these issues and advance conflict research.

The main contribution, apart from a detailed literature review, is the conceptual discussion of how the new data could be used: as complementary data, for data triangulation and incremental improvement, for data enrichment through common analytical units, or multimodal data fusion. These are discussed with some examples.

Let me first stress that I think this article has the potential to be significant contribution and could show the path forward for the integration of new data types into conflict studies – exactly at time where the character of armed conflict is changing, increased use of heavy weaponry which elevates the potential of this article.

However, I found the article surprisingly superficial. I really don't know what the reader is supposed to take away. The introduction and conclusion are strangely sterile. The authors themselves write the main take away is that "incorporating satellite-derived data into conflict research unlocks new potential for understanding armed conflict more comprehensively and accurately. This is because satellite-derived data offer comparative advantages precisely where existing text-based data fall short." This is very general on the one hand and simply not true on the other. I know this will come across as a negative stance but I am genuinely optimistic this perspective article is worth writing and publishing.

Text-based data relies on reports. It can therefore provide accounts of news events like battles, one-sided or state-based violence (UCDP), violence against civilians, sexual violence, riots (ACLED) etc. It can provide destruction data but this is not how it is typically used because news interest is much more in other features. Satellite data can provide destruction and displacement data and, importantly, its precision is not necessarily driven by news attention cycles. Crucially, this means is not more of the same or a different measurement of the same – it is a completely new world opening up with unknown overlap! The nature of the data generating process implies very specific things about how the new data source can help us understand conflict. Some thoughts: How much overlap will there be most likely? Isn't the attention of satellites also directed? My sense is that displacement, fatalities and destruction are in a very interesting three-way relationship – as buildings get destroyed population is displaced and fatalities should decrease. What does it mean conceptually that we are always measuring destruction events with the satellite data? What is the variation in destruction in the armed conflict data? My guess is this has a lot to do with weapon type but also with strategy/tactics? How? A lot is known about this even without additional data work – just from the conflict studies literature. So, the authors can be a lot more specific about what destruction data would add. Displacement data is even more interesting I think technologically but a whole lot of issues need to be discussed regarding what feature exactly can be extracted and how this would add bias.

Even if the conflict studies perspective is not discussed in that much detail it would still be possible to demonstrate the implications purely from a data perspective. One example with multi-city or multi-region panel showing the different event types would be a great way to demonstrate how satellite data could be used to study to improve our understanding of conflict. If we hold the UCDP/ACLED data next to the satellite panel data for Ukraine, Sudan or Israel – what would we see? I am sure it would be really fascinating and would demonstrate the reader really effectively what the authors mean specifically by the categories discussed in Figure 2.

The discussion around Figure 1 illustrates my concerns. Of course, the spatial resolutions of destruction data are much higher than text-based violence data can be (subject to satellite images being available). But the two are not measuring the same type of event at all. Figure 1 is like showing night-light has higher spatial granularity than GDP data without discussing the relationship between the two. When do battles in the city destroy buildings and when do they not? If we have reliable methods of tracking destruction down to five-day granularity why do the authors not show a weekly timeline of the UCDP/ACLED data next to the destruction data for the case in Figure 1? I found this extremely puzzling.

Human coding is, astonishingly, not central to the conceptual discussion. None of the methods to generate conflict data are completely automated. The authors acknowledge that the main text-based methods are human validated by experts. No humanitarian agency acts upon automated data alone. So, where it matters satellite data is still human based. Discussing data types, their complementarity or fusion without the human at the centre is therefore not revealing the full picture. All elements in Figure 2 are conducted by humans in the end – so how will satellite data make the humans better? How can automation be made more complementary to what humans "see" at the moment? The discussion of high-resolution vs. low resolution imagery illustrates this in a powerful way. Humans work with high resolution. This means the high resolution builds the core validation for low resolution images – whether it is automated or not. How to best combine the sources? It's domain transfer problems galore. I am completely onboard with the promise of higher revisit rates but how this interacts with validation at high resolution is not discussed in enough detail.

Referee #2

(Remarks to the Author)
The manuscript addresses an important and timely topic: how satellite-derived data can enhance conflict research by complementing traditional, text-based event datasets. The idea of organizing different integration strategies into a four-level framework is helpful at a conceptual level, and the overall motivation of the paper is well grounded. However, in its current form, the manuscript does not meet the criteria for publication in Nature. Below, I explain the main concerns in detail.
1. The review is not comprehensive. The paper does not fully cover the state of the field. While it cites many relevant studies, the treatment is uneven. Several recent and influential works on operational satellite monitoring of conflict impacts, AI-based damage detection, and practical use of geospatial data in humanitarian contexts are either missing or only briefly mentioned. The discussion of existing tools and datasets tends to be illustrative rather than analytical, and there is little effort to compare approaches, evaluate limitations, or draw general lessons.
As a result, the review gives a high-level overview but does not offer a systematic synthesis of what has been achieved, what gaps remain, and how these should be addressed.
2. The conceptual framework lacks originality and depth. The proposed four-level integration scheme (complementary use, triangulation, enrichment, fusion) is logically structured, but not analytically strong. It reads as a classification rather than a conceptual framework. The levels are not grounded in existing theory on data integration, multimodal learning, or geospatial fusion. No clear criteria are given for what separates one level from another. The framework is not applied to real-world cases to show how it can be used in practice or evaluated. The framework is useful for organizing examples but does not offer new insight into the dynamics of conflict, the logic of integration, or the methodological trade-offs involved.
3. The treatment of advanced methods is speculative, despite the title of the manuscript. The final integration level (data fusion) is described as a promising frontier, but the discussion remains theoretical. The paper does not include any examples of successful fusion models applied in conflict research. Instead, it draws from unrelated domains such as medicine and ecology. While these analogies are interesting, they do not demonstrate feasibility in the conflict domain, nor do they provide a roadmap for how such approaches could be adapted. Without working examples or technical detail, the argument for fusion remains hypothetical.
4. The manuscript touches on important challenges, such as uncertainty, detection bias, and the ethical risks of satellite surveillance. However, these issues are only briefly acknowledged and not examined in depth. For example, the following questions are not covered. What are the risks of misinterpretation when relying on satellite indicators? How should conflicting signals from text and imagery be handled? What governance or validation structures are needed if satellite data are to inform high-stakes decisions? The paper could be strengthened by addressing these questions more directly, rather than assuming integration is always beneficial.
5. The paper discusses the potential value of satellite data in conflict research, but does not show where or how this integration has made a difference in real-world settings. There is no assessment of whether satellite-derived insights have influenced humanitarian response, policy decisions, or public understanding in recent conflicts. This limits the contribution as a forward-looking review.

Referee #3

(Remarks to the Author)
Thank you for the opportunity to read this piece, which has been submitted as a review article for Nature. Generally, I think

this is a well-written and fair essay on a timely subject that would be of interest to many readers. I am, however, a conflict scholar and, like the authors, I find this kind of review quite interesting!

I know that the goal of the journal is to have these types of pieces be broad, however, so I recommend the authors do more in the beginning of their essay to 'frame up' the importance of this work. Mostly, what kind of phenomena or evidence or empirical conclusions are we missing right now through the data limitations the authors outline? Or, perhaps there is a human rights angle to be made here – i.e., that not only our studies are incomplete but our information about the nature, scale, and destruction of war is poorly documented in general which matters for all kinds of media creation and decision making. Further, as evidenced by timely political changes in today's modern era, researchers cannot rely on governments to report and track this information which makes it more important that scholars have ways of triangulating data to increase data quality on this important subject.

The authors appropriately cite existing literature on conflict processes and are clearly familiar with relevant, most-used data sources in the field. I was surprised to not see more of the spatial or network literature here, which seems quite relevant to the question of data quality and the consideration of conflict as a complex, multi-pronged process.

I'd like to know more about attribution. Namely what issues will scholars face as they try to combine satellite data on war damage, for example, with event data from newstreams? The authors write that 'we must deepen our understanding of when, why, and how conflict actors destroy structures…" (p.6) but they say little about how this would work in practice and how we will be able to assign specific acts of destruction to specific actors. Similarly, I wanted a bit more 'show rather than tell' from the authors in the section on displacement. My understanding of data on population flows during conflict is that the data is mostly terrible and unreliable. Is the suggestion here then to combine satellite data with IDP data, for example? Or to replace it? I did not fully follow the first sentence of the last paragraph in this section ("Combing data on …"). Can the authors be more specific here?

Finally, I have concerns about the precision of satellite data and am left wondering if the authors are over-selling it. I do not have much familiarity working with satellite data, however, so my comments might be limited here. Nevertheless, satellite data carries some known (general) limitations. Data quality issues arise from sensors, geometric distortions from satellite positions are common, and there seems to be a great deal of temporal variation due to things like weather and cloud cover. While some limitations are discussed (p.10) I found it concerning that these other more common limitations were not discussed, particularly since attributing physical changes to conflict could be a fraught enterprise.

Best of luck with revisions.

Referee #4

(Remarks to the Author)
The authors effectively highlight that text-based data can offer insights into the who (event actors) and why (motivations), based on narrative descriptions. However, it falls short in accurately capturing what, where, and when due to limitations such as reporting delays, vague spatial references, and a lack of quantitative detail regarding damage or casualties.
Conversely, while satellite data lacks narrative context and cannot typically answer the who or why, it excels at providing precise temporal and spatial information (when, where, and potentially what), particularly when using high-resolution commercial imagery.
The authors proposed four methods of integrating both data sources in conflict research , Complementary integration , which is simple and accommodates broad use; data triangulation which is simply using satellite data to improve the quality of conflict data: data enrichment which integrates both into a single dataset but for a common research unit e.g. for environmental conflict ; and data fusion, which is used to jointly capture a single concept. That is, in conflict research , once a clear definition of what needs to be detected, the model can be trained to identify for instance whether a destruction is an armed conflict destruction in a particular region or not. The text data provides the 'who' and 'why' while the satellite data answers the question of 'where', 'what', and 'when'. For instance, to verify whether armed conflicts occur in a specific location, a model is trained with multimodal data.
I found the analysis of the two data sources to be well-researched and clearly presented. The proposed integration strategies are both practical and inspiring. However, I would have appreciated a more in-depth discussion or evaluation of these four methods. While this may be outside the manuscript's current scope, assessing the complexity and feasibility of data fusion would have added value. For instance, how can we reliably distinguish between conflict-related destruction and damage from natural hazards, especially when access to high-quality satellite data is restricted?
good overview of the characteristics/shortcomings of satellite-derived data and text-based data for conflict research. Furthermore, the authors have successfully presented extant research into the integrative use of these two different data types. I particularly agree with their hierarchical classification of the presented categories of integration, namely;
• Complementary data use
• Data triangulation and incremental improvement
• Data enrichment through common analytical units
• Multimodal data fusion.

A minor comment I have is that the authors have not highlighted the potential challenges that each of the categories possesses. For Multimodal Data Fusion, one major challenge occurs when we go beyond basic computer vision models (for straightforward object detection/classification) to incorporating advanced satellite image processing capabilities (e.g., optical + SAR + Lidar fusion, SAR analysis, etc.) in a fully automated pipeline, as suggested.

Overall, though the concepts may not be entirely new, I did enjoy the paper. The suggestion is white pragmatic, and I am encouraged to further explore the data fusion approach.

Version 2:

Reviewer comments:

Referee #1

(Remarks to the Author)
see attachment

Referee #2

(Remarks to the Author)
The paper looks at how satellite data can be integrated with text-based conflict event datasets and presents a four-level framework of complementary use, triangulation, enrichment, and fusion. The topic is important and timely, and the manuscript covers a wide range of methods in a clear way. However, the overall contribution is limited. The first three levels of the framework are not new. Similar ideas have already been published, for example in Donnay et al. (2019, Journal of Conflict Resolution 63:1337–1364), which explained how conflict datasets can be combined by using complementary coverage, cross-validating overlaps, and adding unique attributes from different sources. The fourth level, fusion, goes a little further, but here it is described only in general terms without concrete evidence or examples. For a Perspective article in Nature, which should provide a distinctive and forward-looking argument, this overlap with existing work and the lack of originality is a serious weakness.
The paper also suffers from weak empirical support. The case studies on Ukraine and Gaza are descriptive and do not provide systematic validation or quantitative results. There is no demonstration that the proposed framework actually improves reliability, accuracy, or scope of conflict monitoring. The methodological points are reasonable, but they remain at the level of categorizing approaches rather than showing new insights or paradigm shifts. The work reads more like a structured review than a Perspective.
The conclusions are also not well supported. The article repeats the promise of multimodal integration but does not show how it would be implemented in practice or why it would transform conflict research in a fundamental way. Important issues such as error rates, uncertainties, validation challenges, and ethical risks are mentioned only briefly. The manuscript would need much stronger evidence and clearer vision to reach the level required for Nature.
In summary, this is a careful overview of current methods, but it represents methodological improvements rather than paradigm-changing ideas. It lacks originality, rests on insufficient evidence, and does not deliver the strong forward-looking vision expected from a Nature Perspective. It could be valuable as a review article in a specialist journal, but in its current form it does not meet the standards of novelty and impact for Nature.

Referee #3

(Remarks to the Author)
I very much appreciate the revisions completed on this manuscript by the authors. The contribution of the piece is made clear by being submitted as a perspectives piece. The quality of the argument is strong and compelling enough to gain attention if published in Nature. I do not think the authors need to be asked for further revisions. My only remaining recommendation is to edit section 4.3 to be a bit more precise and transparent about the feasibility of combining satellite data with PRIO grid data (or other widely used geo-referenced event data). I realize the authors are limited on room, but I really wanted to see a case example to illustrate what the data would look like in this case grounded in the violence against civilians questions the author frame a lot of their piece around.

Version 4:

Reviewer comments:

Referee #1

(Remarks to the Author)
Thank you for taking my comments into account. I can clearly see the contribution now and I think this will be a very useful paper to bring the debate forward. My comments are only minor now (all circle around the same issue) given the paper has very clear strengths now.

First, I think there is substantial work on capturing non-lethal events with text which are not consistently mentioned. ACLED here is leading with riots and sexual violence but projects like GDELT, ICEWS and POLCAT all show that event data generated from text can be quite rich. The key constraint is indeed the focus on news events as a driver of what becomes

data and here is where the satellite data adds tremendously. I just make this point to warn of looking too dismissive of the text data in the beginning of the paper – this data is very rich and is used to track all kinds of events “below” fatalities. The point really is that news reporting and even eyewitness reports that are reported to NGOs will focus on different types of events than building and crop destruction which could take place exactly where no human other than the perpetrators are present. This point is made very nicely in the case studies but has not made it to the earlier part of the article I feel.

Second, this directly brings me to the data-generating process Figure. I think it has many nice elements but I don’t fully agree on how this is done. The first point in text analysis is: “Depends on political regime, media access, humanitarian presence, communications infrastructure, and perceived newsworthiness” whereas the satellite side the first point is “Events with physical manifestations”. This does not suggest a symmetry in the way the data-generating process is tracked and evaluated. Day and night cycles, seasonal changes in vegetation, technical machine learning problem with missing labels, cloud coverage and satellite angles are more similar to news bias and availability. Or should it be "eyewitnesses available" for the first point for text? In my opinion, the point here should really be thinking about how the data is captured through sensors and processed and compare this to how eyewitness reports reach the database of the researchers. The authors admit later that automated coding is lacking for most conflicts - precisely because the labels and technology are still lacking. This is currently reflected by a yellow box in Figure 1 that makes it look like a minor issue. But it is a massive bias. Figure 1 is valuable both as a vision – if we can improve destruction data further to reach fuller coverage – and as a description of reality with all the abovementioned biases. But it is not clear what it wants to be. It looks like depicting reality for text and like proposing a vision for destruction data.

Third, my previous point can be seen in the case selection of Ukraine/Myanmar – was there always coverage on all time slices/locations? Or was there an satellite operator/purchase that was needed to get the images of the villages that were destroyed in Myanmar? What were the underlying organizational biases in UNOSAT here? I really like the cases and they are powerful but I think they are clearly hand-picked and it is not clear from this what would happen with a systematic adoption/availability of this sort of data. I think Figure 1 could play a role for that – to evaluate what could be missing from satellites and how the two sets are truly complements. The displacement example, which is now even implemented (amazing) can really help. Eyewitness reports are scarce exactly where violence is most vicious. Displacement is most permanent where destruction is taking place. So, destruction data would really help us understand displacement data differently. News event data misses this because displacement is negatively correlated with eyewitnesses (mortality risk is a direct driver of data availability for text data).

Turning towards the cases – I think this is a very strong part of the paper as it now stands. I really liked all the discussion here and I also went through the methodology and appendix section and learned a lot. This is great work. My only nag is that the lack of connection between the Figure 1 and this section. The discussion in the cases is very rich. The Myanmar case relies on hand coded data, Ukraine on a true panel dataset (!) of destruction data. These are very different things and it is not clear what data generating process underlies the UNOSAT manual coding – is this the universe of burned villages in this conflict? Could it be that news and eyewitness accounts played a role here? The authors themselves write towards the end of the article that only some countries are coded with destruction data. So here we seem to be still on a technological frontier. What drives whether destruction data is available? This is not discussed systematically but it is discussed for the text data.

This brings me to my final point. I think it is exactly in the interaction between conceptual thinking about the interactions between text and image, ordering the literature along these lines and also ordering the analysis part into this where I see the clearest way to contribute. The case studies need to be elevated with some sort of a framework that helps us to understand where satellite data adds. But at the moment the authors seem to inflate the availability and readiness of destruction data and do not systematically mention that most conflicts and most areas are not covered. What is the bias in this selection?

(Remarks on code availability)

Referee #3

(Remarks to the Author)
This article offers an important contribution to the study of armed conflict. The main point of the piece is to argue that satellite data offers a new and complementary data stream to existing text-based sources. This core argument is, overall, a success. The article is original, thoughtful, and high quality.

The authors could improvement the article in two ways.

First, for the "improvement" approach, can the authors clarify the assumption that killing and destruction occur in the same location? In the case of Ukraine, for example, it is unclear how to think about prior destruction that predates current day events. This also relates to measurement error, both off-the-shelf event data (from news stories) and satellite data are subject to measurement error. The combination of the two data streams produces a third category of error-- what does this look like, how does it amplify uncertainty when you think about the error together rather than each as categorically separate? Is there a dependency structure here that matters for inference? I.e., are the errors systemically correlated? (Low reporting environments correlated with poor satellite detection?)

Second, while the authors acknowledge case selection limitations in the 'way forward' section the mention is very brief.

Perhaps in the supplemental materials there could be a table that suggests which cases these methods would excel in, which they would not, and why? Wouldn't the methods work less well for conflicts taking place in dense forests, with low tech violence, and/or a lot of clouds? It is notable that neither of the cases used in for illustration are in Africa or Latin America. I think there is a bit of a tendency of the authors to oversell the contribution.

Nevertheless, I commend the authors on a clear and novel approach.

(Remarks on code availability)

Referee #5

(Remarks to the Author)
The authors compare satellite imagery & methods to media-reported events for studying conflict. The manuscript is well-written and provides three ways to integrate the data to provide a richer picture of conflicts. The authors provide novel examples for two of the integration methods and outline a path forward for the third. The approach should be of interest to many conflict researchers. The authors have done a good job bringing together data and methods to provide examples of what they are proposing. I have the following suggestions that should be addressed prior to publication:

The biggest weakness I see is that the authors do not mention survey methods or interviews or other boots-on-the-ground fieldwork as a way of measuring the effects of conflict. This could be addressed by constraining the scope of the manuscript to only consider 'remote' conflict studies, but I don't think this what the authors intended. Instead, I think it makes more sense to add a discussion about these other ways of measuring conflict.

Examples include work from researchers such as John O'Loughlin, Gerard Toal, and Kristen Bakke who regularly conduct large surveys with many 1000s of respondents in active or recent war zones. Door-to-door surveys are very effective, but for places too risky, phone surveys can be effective. Other large surveys such as Afrobarometer are regularly used to study conflict in Africa.

These other contributions to conflict are important since the manuscript concludes with a call for "a fuller picture of violence in armed conflict". Such a fuller picture cannot be achieved without talking with people on the ground. For instance, rape is a common weapon of war that rarely gets picked up in the kind of news media described in the manuscript. Other effects such as psychological trauma associated with witnessing violence or losing family/friends to the war are similarly not captured.

I'm not sure exactly where these kinds of less-visible effects from violence fit into Supp Info A. Seems like it is a direct human effect of conflict, but none of your data discussions address it. Perhaps the 3 categories identified need to be re-thought or explained more fully.

Adding in these dimensions & methods of data collection will likely require modifying Fig. 1a, or coming up with some sort of justification for why you are not including it as part of the study of conflict data generation. Or perhaps phone surveys would count as 'Remote sensors' on Fig. 1b, but that would really throw things off.

And even if you want to exclude data collected from surveys and interviews, there is also an emerging field of 'digital fieldwork' that should be incorporated as a way of studying conflict. Here are some recent relevant citations (I am not affiliated with either):
Limonier, K., & Audinet, M. (2025). From restricted to digital fieldwork: A renewed methodological framework for Russian studies after the full-scale invasion of Ukraine. Communist and Post-Communist Studies, 1–15. https://doi.org/10.1525/cpcs.2025.2474792
Sereda, V., & Mikheieva, O. (2025). How (not) to study a war-affected society: Challenges of knowledge production in Ukraine and elsewhere. Nationalities Papers, 1–20. https://doi.org/10.1017/nps.2024.56

A few more thoughts on Fig. 1 since it is ripe for picking at. Overall, I like the concept of the figure, though as I mention above, it is incomplete. For the Remote sensors side, I think it would be worth adding "Detection uncertainty" to the Manual annotation box since there are plenty of war effects that are not obvious to the human eye in the imagery. For instance, is that a bomb crater in the field? Is that structure damaged, or does it just have an oddly shaped/colored roof?

Also, still on the Remote sensors side, the Automated coding items seem to be specific to SAR data analysis. I guess I am thinking this based on "Underrepresentation of certain types of events and small objects". With computer vision and AI methods, I think we are at a point where it would be possible to automate the Manual annotation approach using optical satellite imagery. It might be expensive, but someone could use all high-res cloud free imagery over a large (global?!) conflict zone and have an AI annotate areas of damage. Indeed, I just made this suggestion to someone at a conference last week who had full access to Maxar/Vantor's high res imagery.

Analysis Comments

At the end of Section 3.1, the authors claim that "Russian forces destroyed substantially more buildings when taking over territory than when Ukraine recaptured territory". But I don't see where this is normalized by area. Perhaps I am missing something, but does Fig. 3c just reflect that more territory was captured by Russia than by Ukraine? If it's just a reflection of

total area captured, then the conclusion does not seem very interesting.

Related, isn't it likely that territory recaptured by Ukraine had already undergone considerable destruction during the prior fighting, so there was less opportunity for more destruction? Furthermore, even if more buildings were destroyed when Ukraine re-took the area, the SAR CCD method would not detect it if it had already marked that pixel area as 'damaged'.

Also, the error bars for Fig. 3c are not defined.

For Fig 5c & 5d, I wasn't able to easily figure out where the Estimated damage numbers came from. I assume it is described in section 5, but can you please make this clearer?

Though I did not run all of the provided code, I reviewed the Python and R code and did not find anything particularly problematic.

Minor:

Fig. 1 -- some of the arrows/lines need to be cleaned up.

Appendix A is never cited in the text.

It would be helpful to add labels to each of the Fig 4 & 5 panels to denote the different townships. Also in Figs D6 & D7.

(Remarks on code availability)
I did not run the code due to time constraints. The readme.md file appears to be sufficiently detailed for this to be a successful endeavor, but different machine configurations can always pose a challenge. Given the number of co-authors on the manuscript, I suggest asking each of them to run the code on their own machines since they have more of an incentive for publication than I do.

Version 5:

Reviewer comments:

Referee #1

(Remarks to the Author)
I read the new version and the replies with great interest. All my comments were addressed.

Minor points:

I am sorry to raise this so late — I should have spotted it earlier — but I don't find Figure 5 particularly intuitive. I think the authors mean to describe the relative shapes of the fatality and destruction series here. Perhaps this could be conveyed better with per-period numbers rather than cumulative ones? Relatedly, I find it confusing that the top panel is in absolute terms while the bottom panel is relative to the total. At the very least, I think it would help to have clearer labels (e.g. "fatalities" instead of "best") and perhaps a visual cue flagging where the authors want the reader to focus.

Data sources in Figures 3, D1 and D2 are listed as [9, 31], but as far as I can tell the territorial control (and tessellation) data come from VIINA 2.0, which is cited as a separate reference in the Methods 69? Perhaps the 31 reference presents the data as well so this is just minor point.

I had some trouble reconciling the sample used in the Appendix D tests with the Methods. The tables are based on 95 Russian and 148 Ukrainian cells, whereas the Methods report 200 and 967 first stable transitions for the frontline period. I assume the difference is a restriction to populated areas, but the intermediate steps are not spelled out. A short sample-construction summary would help.

(Remarks on code availability)

Referee #5

(Remarks to the Author)
Thanks to the authors for their careful attention to my prior suggestions. I like the changes to Figure 1.

The only additional suggestion I have relates to terminology and engaging a bit with an existing literature. The first part of the 'Improvement' section (the Fig. 2 examples) seems to be a form of dasymetric mapping or areal interpolation. I suggest

acknowledging this connection to prior work with a citation to Eicher & Brewer (2001) or Mennis (2003) and any other work (maybe Wright 1936) you find relevant.

(Remarks on code availability)
I did not look at the code for this round of review. I did not see the code as part of the submitted documents for review, and the zenodo link appeared to have restricted access. Even if I had access, I would not have been able to spend much time reviewing it.

Version 6:

Reviewer comments:

Referee #5

(Remarks to the Author)
For this review, I did a quick check of the main text file (no additional comments), and focused my efforts on the large code and data repository available via zenodo.

Overall, the code is very well organized and easy to read with mostly self-documenting variable and function names. A few detailed comments here.

The requirements.txt file is duplicated in the 02_ukraine_improvement directory. This is a bit confusing. It seems like it is only necessary for the python code in the subdirectory, so it probably makes sense to delete the top level version of the file and update the README.md to point users to the subdirectory.

In the myanmar_analyses.R code, there appears to be a naming conflict in the comments, with references to Fig. D4 and Fig. D5, but then the actual figures written are FigED1c and Fig ED2c. This is the classic, "comments lie, code doesn't't" problem. Related, I think the README.md file has a typo on line 45:

Extended Data Figs. 1c-e; 5c-e; 6; and 7a-c.
==>
Extended Data Figs. 1c-e; 2c-e; 6; and 7a-c.

With some effort, I was able to run all of the R and Python code on my Windows machine. A few suggestions to hopefully make this process easier for future users:

The run_main.sh bash script is nice, but many Windows users do not have a shell environment installed. I know you can't handle every configuration, but since your project is a combination of R and Python, you might suggest that people use Positron to run the code and then have them use the Git Bash interface in the TERMINAL. This is probably easier than using WSL or Cygwin. In the future, a better solution might be to provide a single Python (or R) top level file that runs everything else. That would remove the bash dependency altogether.

To get it to work for me, I made the following changes:

a) Multiple changes to run_main.sh to get it to find Rscript quickly. See attached revised file.

b) Change line 16 in run_figures2.sh to point to "python" instead of "python3"

c) Add a UTF argument to the path.write_text calls in statistical_tests_first_gains.py and spatial_robustness_first_gains.py files:

path.write_text("\n".join(lines) + "\n")
==>
path.write_text("\n".join(lines) + "\n", encoding="utf-8")

(Remarks on code availability)
See above code-specific comments and the attached file, run_main.sh.

Open Access This Peer Review File is licensed under a Creative Commons Attribution 4.0 International License, which permits use, sharing, adaptation, distribution and reproduction in any medium or format, as long as you give appropriate credit to the original author(s) and the source, provide a link to the Creative Commons license, and indicate if changes were made.
In cases where reviewers are anonymous, credit should be given to 'Anonymous Referee' and the source.
The images or other third party material in this Peer Review File are included in the article's Creative Commons license, unless indicated otherwise in a credit line to the material. If material is not included in the article's Creative Commons license and your intended use is not permitted by statutory regulation or exceeds the permitted use, you will need to obtain permission directly from the copyright holder.
To view a copy of this license, visit https://creativecommons.org/licenses/by/4.0/

Revision Memo

Dear editors, dear reviewers:

Thank you for the opportunity to revise this manuscript and for your thoughtful comments, which have greatly helped us strengthen it.

We are submitting a substantially revised version of the manuscript that incorporates your feedback. In particular, we have developed a stronger conceptual argument and a clearer framing of the main contribution of integrating satellite-derived data into conflict research; strengthened the discussion of each data integration category; included more recent literature; ensured that our discussion of existing approaches is analytical rather than descriptive; and more clearly outlined the limitations and challenges of satellite-derived data for conflict research. We have made every effort to address all reviewer comments while staying within the word limit.

Please note that we are resubmitting this manuscript as a Perspective article and have revised it to emphasize a forward-looking research agenda in this emerging field.

Below, we respond to each individual comment and explain how it was addressed in the revised version. We hope these revisions address your main concerns and remain open to further changes if required.

Editor comments:

Dear Dr Sticher,

Your Review, "Advancing conflict research and response through satellite-derived data", has now been seen by four referees, whose comments are appended below. You will see that our reviewers find your piece both timely and interesting. Nevertheless, they have raised points which we feel should be addressed to their satisfaction before we can make a final decision on publication.

In particular, you will see that although referees are generally enthusiastic about the paper, they raise important concerns about the contributions to the literature and clarity of the argument. As these are concerns we share, we will only be able to consider a revision that clearly demonstrates the following:

-More depth of the discussion of each proposal/classification category (all reviewers)

We have considerably expanded the data integration section to provide greater depth to the discussion of each category (which in the article we now call data

integration types). While the four categories themselves remain unchanged, we now make clear that this is a classification scheme that we propose, organized along a single dimension: the degree to which the two data modalities are linked in the process of integration. We now also much more clearly link the categories to the overarching conceptual framework of the article. Specifically, we discuss *complementarity* of text-based and satellite-derived data (when the two data types capture different types of events) and *overlap* (when the two data types capture different manifestations of the same event). We then show how data integration leverages complementarity (category 1 and 3), overlap (category 2), or both (category 4) to advance our analytical capabilities and insights.

To make the discussion more substantial and to address the reviewers' comments, we have also adopted a consistent structure across categories: We first describe the approach in greater detail and clarify how it differs from the others. We then assess whether it primarily leverages complementarity or overlap between satellite-derived and text-based data. Each category now also includes a concrete example that illustrates its added value in practice and highlights the kinds of research questions that would become answerable through this type of integration, but are difficult to answer with existing data. Finally, we conclude each discussion by going through technical data integration challenges, alongside conceptual challenges, that is, the steps that need to be taken for this type of integration to lead to meaningful insights. This way, each category now not only describes a promising path but also outlines the conditions under which it could realistically be implemented to advance conflict research.

-Discussion about larger questions in relation to data validity and attribution (all reviewers)

We agree that the previous version of the manuscript was lacking sufficient information about data validity and attribution and have strengthened this throughout the text. In Section 3.2, we are more specific about what aspects of displacement satellite-derived data can capture and how this helps us better understand conflict dynamics. In Section 4, in each data integration category, we discuss challenges of integration, including aspects of data validity and attribution, drawing clearer connections between what the data represents and how we can link it to known events from text-based data. We clarify the distinction between complementarity and overlap in text-based and satellite-derived data, and show how complementarity can be leveraged to capture a broader spectrum of conflict-related violence, while overlap can be leveraged to attribute spatial or other information to known events, thus strengthening existing data. Finally, in section 5, a new section on Challenges and Limitations, we discuss limitations related to satellite-derived data and how they affect data validity.

-The inclusion of more recent literature and network/complexity literature (R2, R3)

We have revised the manuscript to incorporate more recent literature, particularly in Section 3, where we provide an overview of approaches and the types of phenomena they can capture. The discussion now includes a more comprehensive discussion of the literature about damage to the natural environment, and we have added a more extensive overview of recent work on approaches to collect data on displacement. Please note that, since we are resubmitting this as a Perspective rather than a Review, our aim is not to provide a comprehensive survey of existing studies but to highlight the most relevant efforts that shape the way forward.

We have also added literature on spatial dynamics, including references to the local turn in conflict studies (Section 4, Complementary data use) and on the complexity-oriented and integrated study of conflict-related violence (Section 4, Data enrichment). However, based on our understanding of the network literature in conflict research, network studies are concerned with how actors relate to each other, and we do not see particular promise in satellite-derived data for this specific point. We are open to integrating literature on this angle if we misunderstood the comment (see response to R3).

-The need to develop a more conceptual argument (R1, R3)

We agree that the previous version lacked a clear conceptual argument and thank R1 and R3 for their excellent suggestions. In the revised manuscript, we have introduced a clear conceptual framework and streamlined it throughout the paper. Specifically, we distinguish between two types of targets of conflict-related violence—human and material—and demonstrate how violence directed at these targets can be complementary or overlapping. When violence is complementary—for instance, when actors destroy crops and houses to render an area uninhabitable, without killing people—satellite-derived data can help capture a broader spectrum of conflict-related violence. When violence overlaps—for example, when houses are bombed, resulting in both destruction and fatalities—such data can add spatial precision to existing conflict event datasets or enrich satellite-derived evidence with contextual information and narrative depth. In Section 4, we discuss how different levels of data integration draw on complementarity (category 1 & 3), overlap (category 2), or both (category 4) to enable a new research agenda and enhance our insights into long-standing questions.

-A discussion on the limitations of the data (human validation from R1, general limitations of satellite data from R3).

We have added a new section, Section 5 (Challenges and Limitations), to do justice to this point. In this section, we focus on the limitations of satellite-derived rather than text-based data, as the limitations of text-based data have been extensively documented and are a key rationale for writing this Perspective (as discussed in the

introduction and in Section 2). Alongside technical and conceptual challenges, we also discuss dilemmas that researchers face, including trade-offs between manual and automatic detection and challenges of human validation, especially for contexts where no reference data is available.

Although reviewers assessed this text as a Review, we still see a case for a Perspective as well based on the four proposals. Any of these formats work for now, and depending on the scope of the revisions, it could go either way. The difference is mainly whether you think the text's contributions are more in the literature review of the field (Review) or if in the proposal to integrate satellite data (discussing how, in what cases, etc., as the four proposals to make this a Perspective).

Thank you for your guidance. We see our primary contribution in the latter: outlining how different approaches to collect satellite-derived data generate new insights into armed conflict dynamics, and how such data can be integrated to complement and enrich existing conflict event data. Accordingly, we are resubmitting the manuscript as a Perspective article.

Please also note that some reviewers are asking for more illustrations of the points, which we agree. That said, given the format guidelines, these texts should not carry out data analysis; the examples should be from published findings elsewhere or through a conceptual example, but not with data collection and processing.

We have included two new figures in the manuscript. Figure 1 is a conceptual illustration of the differences of text-based and satellite-derived data. It replaces Figure 1 from the previous manuscript (see response to comment from R1). Figure 2 includes two examples of damage assessments that have been published under a Creative Commons license (CC BY-NC-ND 4.0); for-profit use can be requested.

We therefore invite you to revise your manuscript, taking these points into account. At the same time, we hope you might be willing to make some editorial changes that will ensure accessibility to our broad audience. In addition, as we estimate that your Review currently exceeds our standard page limit by some margin, and we must ask that you shorten it. This can be achieved by attention to the following points.

We have done our best to ensure accessibility to the broad audience of Nature readers and are open to further revisions by the editors. We have shortened the text and follow the formatting guidelines (not included in this revision memo).

Referee #1:

I am writing to provide a review for the article "Advancing conflict research and response through satellite-derived data". This perspective article has the goal to

outline how satellite-driven data can help conflict research advance from where it is now. The authors make the point that, up-until now, text-based measurements of conflict have dominated research which comes with a set of specific issues. The article argues that satellite data can help mitigate these issues and advance conflict research.

The main contribution, apart from a detailed literature review, is the conceptual discussion of how the new data could be used: as complementary data, for data triangulation and incremental improvement, for data enrichment through common analytical units, or multimodal data fusion. These are discussed with some examples.

Let me first stress that I think this article has the potential to be significant contribution and could show the path forward for the integration of new data types into conflict studies – exactly at time where the character of armed conflict is changing, increased use of heavy weaponry which elevates the potential of this article.

Thank you very much for your overall assessment and for seeing the potential in our Perspective.

However, I found the article surprisingly superficial. I really don't know what the reader is supposed to take away. The introduction and conclusion are strangely sterile. The authors themselves write the main take away is that "incorporating satellite-derived data into conflict research unlocks new potential for understanding armed conflict more comprehensively and accurately. This is because satellite-derived data offer comparative advantages precisely where existing text-based data fall short." This is very general on the one hand and simply not true on the other. I know this will come across as a negative stance but I am genuinely optimistic this perspective article is worth writing and publishing.

We agree that the previous version of the manuscript lacked a clear central message. In the revised version, we place much stronger emphasis on how satellite-derived data enables us to capture a broader spectrum of conflict-related violence, thereby opening up entirely new analytical possibilities, as detailed below. We have streamlined this focus throughout the text, resulting in a stronger argument for why and how the integration of satellite-derived data can advance the field of conflict research. We have also revised the introduction to underscore the timeliness of our agenda, including reference to the increased use of heavy weapons and drone warfare in contemporary conflicts. In the conclusion we removed some of the more technical discussions and instead emphasize the bigger picture and three areas of efforts needed to advance our research agenda responsibly. More generally, space permitting, we have added real-world examples of what conflict research and

humanitarian actors miss without satellite-derived data, making the text more "hands-on" and hopefully less superficial or sterile.

Text-based data relies on reports. It can therefore provide accounts of news events like battles, one-sided or state-based violence (UCDP), violence against civilians, sexual violence, riots (ACLED) etc. It can provide destruction data but this is not how it is typically used because news interest is much more in other features. Satellite data can provide destruction and displacement data and, importantly, its precision is not necessarily driven by news attention cycles. Crucially, this means is not more of the same or a different measurement of the same – it is a completely new world opening up with unknown overlap! The nature of the data generating process implies very specific things about how the new data source can help us understand conflict. Some thoughts: How much overlap will there be most likely? Isn't the attention of satellites also directed? My sense is that displacement, fatalities and destruction are in a very interesting three-way relationship – as buildings get destroyed population is displaced and fatalities should decrease. What does it mean conceptually that we are always measuring destruction events with the satellite data? What is the variation in destruction in the armed conflict data? My guess is this has a lot to do with weapon type but also with strategy/tactics? How? A lot is known about this even without additional data work – just from the conflict studies literature. So, the authors can be a lot more specific about what destruction data would add. Displacement data is even more interesting I think technologically but a whole lot of issues need to be discussed regarding what feature exactly can be extracted and how this would add bias.

Thank you for this thoughtful comment and for the great suggestions regarding the complementarity and overlap of the two data types, which helped us clarify our conceptual framework. We are now much more explicitly discussing data complementarity (satellite-derived and text-based data capturing different kinds of events) and overlap (the two data types capturing the same events, but recording different manifestations or consequences of the violence).

In the introduction, we take up the central message by stating that: " Pursuing these forms of integration represents a critical research frontier that could not only mitigate limitations of existing data but unlock entirely new analytical capabilities, enabling researchers to examine how different forms of violence interact and shape realities on the ground."

In Section 2, we introduce two main types of targets—human and material—and show how they can be complementary or overlapping. Building on your example, we discuss the potential three-way relationship between fatalities, displacement and destruction, arguing that the lack of systematic data means that we do not fully understand these dynamics and interactions.

In Section 3, we are clearer regarding what we can and cannot capture through satellite-derived data, by outlining how this data type fundamentally differs from text-based data.

We then show how satellite-derived data could enable us to get a fuller picture of conflict-related violence. We discuss how damage to the built and natural environment can result from violence addressed at either humans or material targets, resulting in either complementary or overlap between text-based and satellite-derived data. We also discuss when and how satellite-derived data can capture the human toll of violence beyond material damage. Focusing on displacement, we argue that while satellite-derived data does not capture displacement directly, it can provide important information about origins, destinations, and trajectories.

In Section 4, we show how the different categories of integration can help us leverage complementarity and overlap. Category 1, complementary use, and category 3, data enrichment, enable us to understand a broader spectrum of violence and study how types of violence relate, by leveraging the fact that satellite-derived data can capture different kinds of events (complementarity). Category 2, triangulation and improvement, enables us to get more spatially precise text-based data and more contextually rich satellite-derived data, when the data captures different manifestations of the same events (overlap). Category 4, fusion, draws from both complementarity and overlap.

It is also in Section 4 (Triangulation) that we briefly pick up on an important question you raised, namely that overlap depends on the type of warfare, weapon type, strategy/tactics.

In Section 6, Way Forward, one of our key points now relates to awareness that choices by researchers shape what types of violence we are able to capture, and where. We call on researchers to extend research to cover regions and types of events that receive less attention, even when models are more difficult to train and validate (due to limited reference data and potentially more challenging settings, such as infrastructure-poor regions). Importantly, while the availability of commercial imagery may be shaped by factors such as international interest in a conflict, open-access satellite data is typically available with global coverage, enabling such research.

Even if the conflict studies perspective is not discussed in that much detail it would still be possible to demonstrate the implications purely from a data perspective. One example with multi-city or multi-region panel showing the different event types would be a great way to demonstrate how satellite data could be used to study to improve our understanding of conflict. If we hold the UCDP/ACLED data next to the satellite panel data for Ukraine, Sudan or Israel – what would we see? I am sure it would be

really fascinating and would demonstrate the reader really effectively what the authors mean specifically by the categories discussed in Figure 2.

Thank you for this suggestion. We agree that adding a multi-city or multi-region panel showing different event types would help demonstrate how satellite-derived data can improve our understanding of armed conflicts, especially when it shows different combinations of different types of events (fatalities, destroyed buildings, destroyed crops, displacement; contexts where these events overlap and where they capture manifestations of different events). We thought carefully about how we could do this while adhering to the editorial guidelines, which preclude any data analysis for this type of article (see Editor comment). Unfortunately we were not able to do an actual panel without additional data analysis, as there is no ready-made data available for cross-context comparison (a point that we now make more clear in Section 6). However, we have added a new Figure, Figure 1, that conceptually illustrates what text-based data captures in comparison to satellite-derived data, drawing on the case of Ukraine. Furthermore, we believe that the examples that we added in Section 4 (data integration) make it clearer how the different levels of data integration would enhance our understanding of armed conflicts.

The discussion around Figure 1 illustrates my concerns. Of course, the spatial resolutions of destruction data are much higher than text-based violence data can be (subject to satellite images being available). But the two are not measuring the same type of event at all. Figure 1 is like showing night-light has higher spatial granularity than GDP data without discussing the relationship between the two. When do battles in the city destroy buildings and when do they not? If we have reliable methods of tracking destruction down to five-day granularity why do the authors not show a weekly timeline of the UCDP/ACLED data next to the destruction data for the case in Figure 1? I found this extremely puzzling.

Thank you for this comment. The aim of Figure 1 (in the previous manuscript) was to illustrate just one particular aspect: how destruction data is spatially explicit and precise, compared to text-based data, which is often only attributed to a centroid of a town (or even more aggregated level). However, we understand that the figure appeared as one-sided, as it only portrayed one specific aspect of the data. We also realize that the figure was not ideal as it was based on manual coding (see next point), whereas we focus primarily on the potential of automatization. We have therefore decided to remove the figure from the manuscript. Instead, we now illustrate more conceptually the different characteristics of text-based data and satellite-derived data, by drawing on the case of Ukraine (new Figure 1). This also includes an example from automated building destruction analysis in Ukraine (Figure 1, panel d). In addition, the new Figure 2 shows spatial and temporal patterns of tree crop and greenhouse damage in Gaza, providing the reader another example of the types of satellite-derived data that can be collected.

Human coding is, astonishingly, not central to the conceptual discussion. None of the methods to generate conflict data are completely automated. The authors acknowledge that the main text-based methods are human validated by experts. No humanitarian agency acts upon automated data alone. So, where it matters satellite data is still human based. Discussing data types, their complementarity or fusion without the human at the centre is therefore not revealing the full picture. All elements in Figure 2 are conducted by humans in the end – so how will satellite data make the humans better? How can automation be made more complementary to what humans “see” at the moment? The discussion of high-resolution vs. low resolution imagery illustrates this in a powerful way. Humans work with high resolution. This means the high resolution builds the core validation for low resolution images – whether it is automated or not. How to best combine the sources? It’s domain transfer problems galore. I am completely onboard with the promise of higher revisit rates but how this interacts with validation at high resolution is not discussed in enough detail.

Thank you for this important comment. We agree that the question of human detection/validation versus automation is important, and that this was not given sufficient attention in the previous version of the manuscript.

We now more explicitly discuss this in the manuscript, including through a discussion of the tradeoff between visual inspection and automated detection in the new Section 5 (Challenges and Limitations). We believe that there is a fundamental difference between text-based and satellite-derived data when it comes to automation. Text data, such as media reports, are only generated when something happens, so we get a selection of information that we process. Human coding is feasible because it only looks at a subset—those that mention an event or manifestations of it (which is also why we miss many events, particularly those that are not involving fatalities). By contrast, satellite data are generated frequently for the entire globe, and we have data to process whether or not something happened. While this makes systematic analysis independent of human reporting possible, it is not feasible for human operators to exhaustively and repeatedly go through all the data. This is why visual inspection is usually only done for smaller areas, for assessments of specific cases by humanitarian actors, and occasionally for case studies in conflict research. However, given resource constraints, automated analysis is the only way we view as feasible for large-scale data collections covering entire countries and longer time periods—i.e. the only way to achieve data collections comparable to existing text-based conflict event data (whether the latter are coded manually or not). This is a key reason why now is an opportune moment to engage in this type of research: because there have been important advancements in the automated detection of conflict events.

The comment is also relevant with regard to data integration. Because we focus on systematic data for conflict research, in Section 4, we focus on integration at scale,

meaning approaches that have potential for being developed into automated pipelines. We agree that we are not at the point where readily available pipelines are available that could do this, so this should be read as a forward-looking (and in the case of data fusion, more speculative) element, in line with the new format of a Perspective article. We agree that a more detailed discussion would be desirable about what exactly this would entail–which parts would be easy to automate, and which steps of integration and validation will continue to require human input. Unfortunately, we could not afford to expand the discussion of these points due to the limited word count.

Finally, in Section 6, Way Forward, we highlight that even for automated processes, human choices include the generation of satellite-derived data—through coding decisions and through reference data.

Referee #2:

The manuscript addresses an important and timely topic: how satellite-derived data can enhance conflict research by complementing traditional, text-based event datasets. The idea of organizing different integration strategies into a four-level framework is helpful at a conceptual level, and the overall motivation of the paper is well grounded. However, in its current form, the manuscript does not meet the criteria for publication in Nature. Below, I explain the main concerns in detail.

1. The review is not comprehensive. The paper does not fully cover the state of the field. While it cites many relevant studies, the treatment is uneven. Several recent and influential works on operational satellite monitoring of conflict impacts, AI-based damage detection, and practical use of geospatial data in humanitarian contexts are either missing or only briefly mentioned. The discussion of existing tools and datasets tends to be illustrative rather than analytical, and there is little effort to compare approaches, evaluate limitations, or draw general lessons.
As a result, the review gives a high-level overview but does not offer a systematic synthesis of what has been achieved, what gaps remain, and how these should be addressed.

Thank you for your assessment and for highlighting the elements missing from the review. We agree that our earlier discussion of existing approaches was not sufficiently analytical and that we did not provide a systematic synthesis of what has been achieved, the gaps that remain, and how these should be addressed. In the revised version, we have strengthened the discussion of approaches to capturing conflict events through satellite-derived data and added several recent influential works. Most of these changes are in Section 3:

- In the paragraphs on damage to the built environment, we now more clearly discuss the comparative advantages of different approaches and explain why some are particularly suitable for large-scale data collection in the context of armed conflict, where damage is dispersed both temporally and geographically.

- In the paragraphs on damage to the natural environment, we incorporated several influential recent works and show how they capture different manifestations—both deliberate environmental destruction and longer-term consequences of conflict dynamics more broadly.

- In the section on displacement, we added further relevant studies and more clearly show what kinds of data they enable us to collect in relation to displacement. We also highlight recent work that applies innovative approaches to mobility data collection, albeit often in highly context-specific ways.

To our knowledge, beyond the studies that we cite—which generally focus on selected conflict contexts–no operational satellite monitoring of conflict impacts currently exists. There is also little work on data integration specifically focusing on conflicts. Where appropriate, we point to approaches from other fields to illustrate the potential and raise the question of what domain transfer would entail.

Please note that we have resubmitted this article as a Perspective rather than a Review. Accordingly, our aim is not to provide a comprehensive review of approaches and tools, but to focus on those that, in our assessment, shape the field or hold particular promise for moving it forward. Moreover, while we acknowledge that case studies on practical applications in humanitarian contexts exist and are very valuable, given the focus of our Perspective, we have prioritized studies that contribute to advancing large-scale data collections on conflict events.

2. The conceptual framework lacks originality and depth. The proposed four-level integration scheme (complementary use, triangulation, enrichment, fusion) is logically structured, but not analytically strong. It reads as a classification rather than a conceptual framework. The levels are not grounded in existing theory on data integration, multimodal learning, or geospatial fusion. No clear criteria are given for what separates one level from another. The framework is not applied to real-world cases to show how it can be used in practice or evaluated. The framework is useful for organizing examples but does not offer new insight into the dynamics of conflict, the logic of integration, or the methodological trade-offs involved.

Thank you for your critical evaluation of the conceptual framework. We have addressed this in two ways. First, we introduce a much clearer conceptual

framework early in the manuscript, by introducing two types of targets of conflict-related violence and showing how satellite-derived data can either capture different types of events (complementarity) or different manifestations of the same event (overlap). We take this up in the data integration part, showing how the different levels of data integration allow us to either leverage complementary (category 1 & 3), overlap (category 2), or both (category 4).

We also clarify that the four categories should be understood as a classification rather than a fully developed conceptual framework. In our naming of the types we were mindful of existing categorizations, but given our broad understanding of data integration, no existing scheme in data integration or multimodal learning fully captured all four types. What we do is that we now explain more clearly what distinguishes the types from one another, while also acknowledging that these are intended as prototypes rather than mutually exclusive categories. To make the framework more concrete, we have added real-world cases for each category and strengthened the discussion of what new insights these integrations could bring that are not currently possible. Finally, we conclude each discussion by outlining the methodological trade-offs, addressing both technical and conceptual challenges of each particular category of data integration.

3. The treatment of advanced methods is speculative, despite the title of the manuscript. The final integration level (data fusion) is described as a promising frontier, but the discussion remains theoretical. The paper does not include any examples of successful fusion models applied in conflict research. Instead, it draws from unrelated domains such as medicine and ecology. While these analogies are interesting, they do not demonstrate feasibility in the conflict domain, nor do they provide a roadmap for how such approaches could be adapted. Without working examples or technical detail, the argument for fusion remains hypothetical.

We agree that data integration remains to some extent speculative, particularly in the subsection on data fusion. At this stage, there is little work on data integration in conflict studies, and our prototypes are necessarily forward-looking. Where appropriate, we draw on approaches from other fields to illustrate the potential and to raise the question of what domain transfer would entail. This applies especially to data fusion, as the Reviewer notes. To provide more concrete ways forward and to ground this in emerging insights, we have now complemented the data fusion section with a brief discussion of the requirements and challenges that would arise if methods from other fields were transferred to the conflict domain.

Please note that we have resubmitted this article as a Perspective rather than a Review, in order to reflect the forward-looking and speculative nature of our argument.

4. The manuscript touches on important challenges, such as uncertainty, detection bias, and the ethical risks of satellite surveillance. However, these issues are only briefly acknowledged and not examined in depth. For example, the following questions are not covered. What are the risks of misinterpretation when relying on satellite indicators? How should conflicting signals from text and imagery be handled? What governance or validation structures are needed if satellite data are to inform high-stakes decisions? The paper could be strengthened by addressing these questions more directly, rather than assuming integration is always beneficial.

Thank you for your observation and suggestion on questions that could be covered. We acknowledge that uncertainty, bias, and ethical risks were only briefly mentioned in the previous version of the manuscript. We have addressed this in the current version. We now dedicate an entire section, Section 5 (Challenges and Limitations) to discuss issues related to uncertainty, the potential for bias, and how research attention can shape what we are able to capture in our data.

In the concluding Section 6, we have added a paragraph on ethical issues. We call on researchers to conduct ethical assessments to ensure that new approaches do not create new vulnerabilities or reinforce existing biases, discuss risks of misuse, show what type of satellite data and type of analysis raise particular concern, and make suggestions on steps that can be taken to mitigate risks of misuse.

Finally, we agree that the step from integrated data to high-stakes decisions based on these data represents yet another challenge. This Perspective is primarily focusing on conflict research, and we have therefore more generally highlighted responsibilities researchers have in communicating uncertainties to those using the data.

5. The paper discusses the potential value of satellite data in conflict research, but does not show where or how this integration has made a difference in real-world settings. There is no assessment of whether satellite-derived insights have influenced humanitarian response, policy decisions, or public understanding in recent conflicts. This limits the contribution as a forward-looking review.

We agree that the previous version included relatively few examples of how integration of satellite-derived data has influenced humanitarian response, policy decisions, or public understanding in recent conflicts. In the current version, we are providing more real-life examples (primarily in Sections 3 and 4), drawing on the experience of co-authors from international organizations. However, it is important to note that our Perspective is forward-looking and focuses primarily on the future potential of large-scale data collections, rather than insights from case studies using

satellite data. As we now explicitly state in the manuscript (Section 6), the integration of satellite-derived data into conflict analysis lies at the frontier of three research fields (remote sensing, computer science, and conflict research), with no ready-made solutions and datasets available for immediate use. Accordingly, many of the potential benefits are still speculative at this moment, and we have framed them accordingly.

Referee #3:

Thank you for the opportunity to read this piece, which has been submitted as a review article for Nature. Generally, I think this is a well-written and fair essay on a timely subject that would be of interest to many readers. I am, however, a conflict scholar and, like the authors, I find this kind of review quite interesting!

I know that the goal of the journal is to have these types of pieces be broad, however, so I recommend the authors do more in the beginning of their essay to 'frame up' the importance of this work. Mostly, what kind of phenomena or evidence or empirical conclusions are we missing right now through the data limitations the authors outline? Or, perhaps there is a human rights angle to be made here – i.e., that not only our studies are incomplete but our information about the nature, scale, and destruction of war is poorly documented in general which matters for all kinds of media creation and decision making. Further, as evidenced by timely political changes in today's modern era, researchers cannot rely on governments to report and track this information which makes it more important that scholars have ways of triangulating data to increase data quality on this important subject.

Thank you very much for your assessment and your suggestion on how to frame-up the essay. We have thoroughly revised the manuscript to ensure that we have a stronger framing and are much clearer in presenting what we can gain from integrating satellite-derived data into conflict research. In the introduction and conclusion, we now clarify that integrating satellite-derived data not only mitigates limitations of existing data, but enables us to cover a new spectrum of conflict-related violence, opening up entirely new analytical possibilities. In Section 2, we show how violence that targets humans can interact with material targets, and what we can learn from studying these relationships. In Section 4, for each integration category, we comment on what new questions or empirical insights we would be able to study with such integration.

Thank you also for your comment regarding governments tracking and reporting information. In section 4, subsection on triangulation and improvement, we now discuss a common sourcing dilemma that conflict event data providers face–that local sources are often the most granular, but also potentially the most biased

sources of information–and show how satellite-derived data could help mitigate this dilemma.

The authors appropriately cite existing literature on conflict processes and are clearly familiar with relevant, most-used data sources in the field. I was surprised to not see more of the spatial or network literature here, which seems quite relevant to the question of data quality and the consideration of conflict as a complex, multi-pronged process.

Thank you for your comment and for pointing out specific literature. We have added references to spatial literature, in particular literature that discusses the local turn in conflict studies (Section 4, Complementary data use) and on the complexity-oriented and integrated study of conflict-related violence (Section 4, Data enrichment).

With regard to network literature, we were not entirely certain if we understood your comment correctly. In our understanding, network literature in conflict studies is concerned with how different types of actors relate. We do not see particular promise with regard to this literature emerging from satellite-derived data. Please let us know if we missed an important angle, in which case we are happy to integrate this literature into the Perspective.

I'd like to know more about attribution. Namely what issues will scholars face as they try to combine satellite data on war damage, for example, with event data from newstreams? The authors write that 'we must deepen our understanding of when, why, and how conflict actors destroy structures…" (p.6) but they say little about how this would work in practice and how we will be able to assign specific acts of destruction to specific actors. Similarly, I wanted a bit more 'show rather than tell' from the authors in the section on displacement. My understanding of data on population flows during conflict is that the data is mostly terrible and unreliable. Is the suggestion here then to combine satellite data with IDP data, for example? Or to replace it? I did not fully follow the first sentence of the last paragraph in this section ("Combing data on …"). Can the authors be more specific here?

Thank you for your suggestions and for raising the issue of attribution. Attributing specific acts of destruction to specific actors is not a task where we see satellite-derived data to excel–even for manually coded, text-based data this is often difficult. We removed the quoted sentence as it may be misunderstood. While we believe that in some cases, perpetrators and potentially victims can be inferred from patterns observable in satellite data, the best information on actors will likely always come from text-based data. In Section 4, data triangulation, we now much more clearly highlight how matching text-based to satellite-based data could enhance our understanding of attribution. We also clarify what this means in practice, i.e., that this would entail record linking, and refer to related literature in conflict event data linking/integration.

We also strongly revised the section on displacement, to clarify concrete opportunities of using satellite-derived data to collect relevant data. As we now clarify, “Satellite data cannot directly measure displacement, but can help us identify origins and destinations, estimate population sizes, and—in combination with other data— offer insights into population movements.” We specify the approaches and highlight limitations of collecting such information. Whether or not such data is then combined with other existing information depends on the data integration category, as outlined in Section 4. In our assessment, data on displacement will always be strongest if it combines different data sources.

Finally, I have concerns about the precision of satellite data and am left wondering if the authors are over-selling it. I do not have much familiarity working with satellite data, however, so my comments might be limited here. Nevertheless, satellite data carries some known (general) limitations. Data quality issues arise from sensors, geometric distortions from satellite positions are common, and there seems to be a great deal of temporal variation due to things like weather and cloud cover. While some limitations are discussed (p.10) I found it concerning that these other more common limitations were not discussed, particularly since attributing physical changes to conflict could be a fraught enterprise.

Thank you for this important observation. We realize that the limitations were not given sufficient discussion in the previous version of the manuscript, and have now dedicated a new section (Section 5) to challenges and limitations. Here, we focus on specific limitations related to the collection of conflict-related data, as well as some more general limitations, such as how data quality issues arise from sensors, and the effects of cloud coverage, which can lead to seasonal patterns and disparities between cloud-prone and arid regions.

It is also true that some issues arise from distortions from satellite positions. One implication is the geolocation accuracy of different data. However, while the geolocation accuracy of satellite products is worse than measurements on the ground, their spatial precision is still extremely high compared to traditional conflict event data. For example, the measured geolocation accuracy of the Sentinel-2 Level-1C product is <11m at 95.5% confidence level while their multitemporal registration accuracy (i.e., how well multiple Sentinel-2 Level-1C products of the same area are co-registered on top of each other) is <5m 95.5% confidence level (see here). By contrast, text-based data is usually not available with precise geo-location. Instead, events are often attributed to a centroid of a town or—especially for rural areas—even only attributed to a district or a province. Accordingly, compared to text-based data, the spatial precision of satellite-derived data is extraordinarily high. We have therefore not listed this as a limitation. However, one area where the difference between measurement on the ground and measured geolocation accuracy of satellite data matters is when multiple data

sources are combined to detect conflict events. Because satellites have varying levels of positional errors, simply overlaying their data can produce highly uncertain results, requiring careful accounting for these errors in analysis. We have considered including this point in the revised text, but ultimately decided that it is primarily a technical issue that remote sensing experts are well aware of, and have removed it due to the very limited word count of the publication format. We do, however, make the more general point that automated detection of conflict events requires technical expertise, part of the reason why we see this as a truly interdisciplinary research agenda.

Best of luck with revisions.

Thank you!

Referee #4:

The authors effectively highlight that text-based data can offer insights into the who (event actors) and why (motivations), based on narrative descriptions. However, it falls short in accurately capturing what, where, and when due to limitations such as reporting delays, vague spatial references, and a lack of quantitative detail regarding damage or casualties.

Conversely, while satellite data lacks narrative context and cannot typically answer the who or why, it excels at providing precise temporal and spatial information (when, where, and potentially what), particularly when using high-resolution commercial imagery.

The authors proposed four methods of integrating both data sources in conflict research , Complementary integration , which is simple and accommodates broad use; data triangulation which is simply using satellite data to improve the quality of conflict data: data enrichment which integrates both into a single dataset but for a common research unit e.g. for environmental conflict ; and data fusion, which is used to jointly capture a single concept. That is, in conflict research , once a clear definition of what needs to be detected, the model can be trained to identify for instance whether a destruction is an armed conflict destruction in a particular region or not. The text data provides the ‘who’ and ‘why’ while the satellite data answers the question of ‘where’, ‘what’, and ‘when’. For instance, to verify whether armed conflicts occur in a specific location, a model is trained with multimodal data.
I found the analysis of the two data sources to be well-researched and clearly presented. The proposed integration strategies are both practical and inspiring.

Thank you very much for your overall assessment.

However, I would have appreciated a more in-depth discussion or evaluation of these four methods. While this may be outside the manuscript's current scope, assessing the complexity and feasibility of data fusion would have added value. For instance, how can we reliably distinguish between conflict-related destruction and damage from natural hazards, especially when access to high-quality satellite data is restricted?

We have thoroughly revised and expanded the data integration section to strengthen the conceptual discussion of each of the four levels. For each data integration level, we have added a discussion on what it would take, both technically and conceptually, to achieve this level of integration. As data fusion in conflict studies has not yet been attempted, we build on other fields to raise questions on what domain transfer would entail, addressing also your important point about the complexity and feasibility of such endeavours

Regarding the challenges of distinguishing between conflict-related destruction and damage from natural hazards, we included this as an example of ambiguities that will likely remain in the data. We have taken up your example of natural hazards again in the data integration section to argue that data integration can potentially help address this, by leveraging broader information from texts regarding where and when violence has likely taken place. Specifically, we have added a sentence on this in Section 4, triangulation and improvement, and in Section 4, data fusion.

good overview of the characteristics/shortcomings of satellite-derived data and text-based data for conflict research. Furthermore, the authors have successfully presented extant research into the integrative use of these two different data types. I particularly agree with their hierarchical classification of the presented categories of integration, namely;
• Complementary data use
• Data triangulation and incremental improvement
• Data enrichment through common analytical units
• Multimodal data fusion.

A minor comment I have is that the authors have not highlighted the potential challenges that each of the categories possesses. For Multimodal Data Fusion, one major challenge occurs when we go beyond basic computer vision models (for straightforward object detection/classification) to incorporating advanced satellite image processing capabilities (e.g., optical + SAR + Lidar fusion, SAR analysis, etc.) in a fully automated pipeline, as suggested.

We agree with this assessment and have added potential challenges for each category of integration in Section 4, addressing both technical and conceptual aspects (new last paragraph in each type/category). Data fusion is the most

speculative type we propose, with no existing work in conflict studies to draw on, and we have therefore expanded the discussion of challenges and requirements in that part in particular (last paragraph in Section 4: Data Fusion). However, given the limited word count of the Perspective, we were unable to go into much detail to discuss the challenges involved in incorporating a full multi-sensor, multi-data pipeline.

On a more general level, we pick up your comment in the last paragraph of the Perspective. Even for basic computer vision models—let alone fully automated multi-modal pipelines—we are not yet at a point in conflict research where ready-made global solutions and data are available for immediate use. This is why everything we propose is forward-looking. We hope this addresses the spirit of your comment, namely that the technical challenges are substantial, and we have tried to highlight these throughout the piece.

Overall, though the concepts may not be entirely new, I did enjoy the paper. The suggestion is white pragmatic, and I am encouraged to further explore the data fusion approach.

Thank you!

Revision Memo

Dear Editors and Reviewers,

Thank you for your thoughtful comments on our earlier manuscript and for the opportunity to submit a revised version. Please find the new manuscript attached.

We agree that empirical illustrations are essential to demonstrate how the integration of text-based and satellite-derived data can be implemented in practice. To enable such illustrations, we are resubmitting the manuscript as an *Analysis*, which is a more natural fit for our core contributions: outlining concrete pathways to data integration, demonstrating their practical application, and highlighting the novel analytical opportunities they enable.

The article follows a deductive structure: we first motivate the need for data integration by highlighting the inherent limitations of text-based and satellite-derived data when used in isolation. We then outline concrete pathways along three levels of integration and empirically illustrate each alongside the insights gained through such integration. This empirical, analytical lens is deliberate, demonstrating on a practical level the unique potential of using satellite-derived data in the study of violent conflicts.

Our case studies—Ukraine and Myanmar—showcase the distinct and novel insights data integration can generate in conflicts with different information environments: Ukraine as a data-rich context and Myanmar as a data-poor one. Our third level of integration, multi-modal data fusion, is explicitly forward-looking and reflects the next research frontier; we therefore limit its empirical illustration to a text-based example.

Given the addition of empirical illustrations and the shorter word limit of the new format, we now present only the core arguments and findings in the main text, moving extended discussions—such as a more detailed overview of the state of the field—into the supplementary information.

We believe these revisions clarify the paper’s contribution and offer a way forward for advancing conflict research beyond a fatality-centric focus toward a multi-modal, multi-dimensional approach to studying violence in armed conflict. Your feedback has been essential in helping us to reshape and strengthen this contribution.

Please find our detailed responses to individual editor and reviewer comments below.

Editor comments:

Dear Dr Sticher,

I apologize for the delay in sending our decision. Your manuscript, "Advancing conflict research and response through satellite-derived data", has now been seen by two of the original referees, whose comments are attached below. In the light of their advice we have decided that we cannot offer to publish the manuscript in Nature in its current form but we are open to a new version that addresses our points below.

While the referees find your work of some interest, they raise concerns about the novelty of the approach and its potential to be executed without some form of illustration or validation. The other two referees became unresponsive despite our many chasers, so we had to proceed with the advice we got. We feel that these reservations are sufficiently important as to preclude publication of the present study in Nature, and we are accordingly closing the file as a Perspective. This is a very specific format that, as we've discussed, does not allow for data analysis. However, given that the referees keep raising this point in this round, we believe that the proposal would benefit from a recategorization into an Analysis. Analyses are priority-setting evidence advances, which means that there is a proposal with data (however preliminary) that shows how it's done — and in that case, this would address the referee concerns with the illustration and will also provide you with some flexibility of what you'd like to show. I would be happy to schedule a call where we can discuss these points if you prefer.

Should you be interested in following this route and further work would allow you to address these concerns, we would be happy to look at a revised manuscript (unless, of course, something similar has by then been accepted at Nature or appeared elsewhere). At such a time, you can request that the manuscript file be reopened for resubmission by simply sending me an email to that effect (stating manuscript number). In the case of eventual publication, the received date would be that of the revised paper. We would contact the some two referees that provided comments but we will have to look for others with overlapping experience with R3 and R4 as we did not hear back from them.

I am sorry that we cannot respond more positively in this occasion, and we hope that you find our referees' comments helpful.

Thank you for your assessment, the suggestion to recategorize the manuscript as an *Analysis*, and the clear guidance on how to proceed. We agree that the Analysis format

is better suited to highlighting the paper's core contribution. Adopting it and adding empirical illustrations required a substantial reworking of the manuscript, but this process allowed us to make our approach and analytical framework more concrete and transparent. We believe these additions sharpen the argument and more clearly demonstrate how the proposed approach can be implemented in practice.

Referee #1:

The article has improved dramatically. I congratulate the authors.

However, I am still concerned about the fact that the conceptual framework is somewhat superficial. It has no formal elements and does not talk about the data generating process in detail. Reading the guidelines by the editor I could see that no data analysis is allowed in the format here. This closes a big potential avenue to illustrate the points of the paper and give them more depth (see below). So, in what follows I will try to bounce some ideas. I know this is not great refereeing in the sense that I am asking a lot but please take the following as inspiration and an attempt for shifting the debate into more concrete territory to clarify my point from the first review.

Thank you for your overall assessment and for engaging so carefully with the manuscript. With the recategorization of the paper as an Analysis, we are now able to illustrate our arguments empirically and develop them in greater depth. We have also included a new section on data generation processes, alongside a new conceptual figure, as outlined below.

Perhaps my previous report was too vague but I think we are missing a map and this is what I would ask of a perspective article. Some ideas, please don't take them literally:

> 1) What are all the measurable dimensions of conflict we could care about and how could they relate to the conflict process? Then after the where are satellites adding and what should be the priority? Some ideas: tents/temporary housing, refugee trails, cars, transport trucks, road destruction, building destruction, vegetation destruction, fires, rubble, night light, market activity, military equipment, weapon craters.

Thank you for this concrete suggestion. We agree that the previous version of the manuscript did not sufficiently clarify the breadth of phenomena that satellite-derived data could, in principle, capture, nor our rationale for prioritizing specific dimensions (damage to the built and natural environment). We address this comment in two places in the revised manuscript.

First, in the main text, we open subsection 2.2 by discussing the wide range of data relevant to conflict-related violence, conceptually distinguishing between three aspects: (1) data on actors and instruments of violence (for example, troop movements or weapon systems), (2) data capturing the direct effects of violence (such as destruction of the built and natural environment), and (3) data on indirect effects (such as declines in agricultural production or market activity). We then explain why we see the greatest potential—and therefore set our priority—in collecting data on the second aspect, the direct effects of violence.

Second, building on this overview, we expand the discussion of these three aspects in the supplementary information (Section A, Data on three aspects of violence). While this section is not intended to be exhaustive, it provides concrete examples of the types of data each aspect may entail.

In addition, Section B of the supplementary information offers a more detailed discussion of satellite-derived data capturing material damage (Section B.1, Approaches to capture war damage) and violence against people (Section B.2, Capturing the human toll).

2) Combine this perhaps with a discussion on dimensions such as: importance for policy, importance for research, measurability with satellites, comparative advantage over text to derive a score of what could be a priority going forward.

This is a great suggestion but given the new article format as an Analysis we can move beyond a conceptual discussion and conduct empirical analyses based on existing data. We have therefore shifted the focus of the second part of the manuscript to demonstrating how data integration can be implemented in practice, outlining concrete pathways across three levels of integration. This approach also enables us to empirically illustrate the analytical opportunities and insights that data integration can generate, rather than assessing the importance of individual dimensions for research and policy in the abstract. This new empirical focus of the paper allows us to address the points you raised, but in a more concrete, hands-on empirical manner, which has significantly strengthened the contribution.

3) I paste the Global Registry of Violent Deaths (GReVD) conceptualization of how different violence measurement types could interact to illustrate how detailed others in this sector have approached similar topics. I know this is not a perfect fit as the goal here was something else. But perhaps this inspires the authors to more.

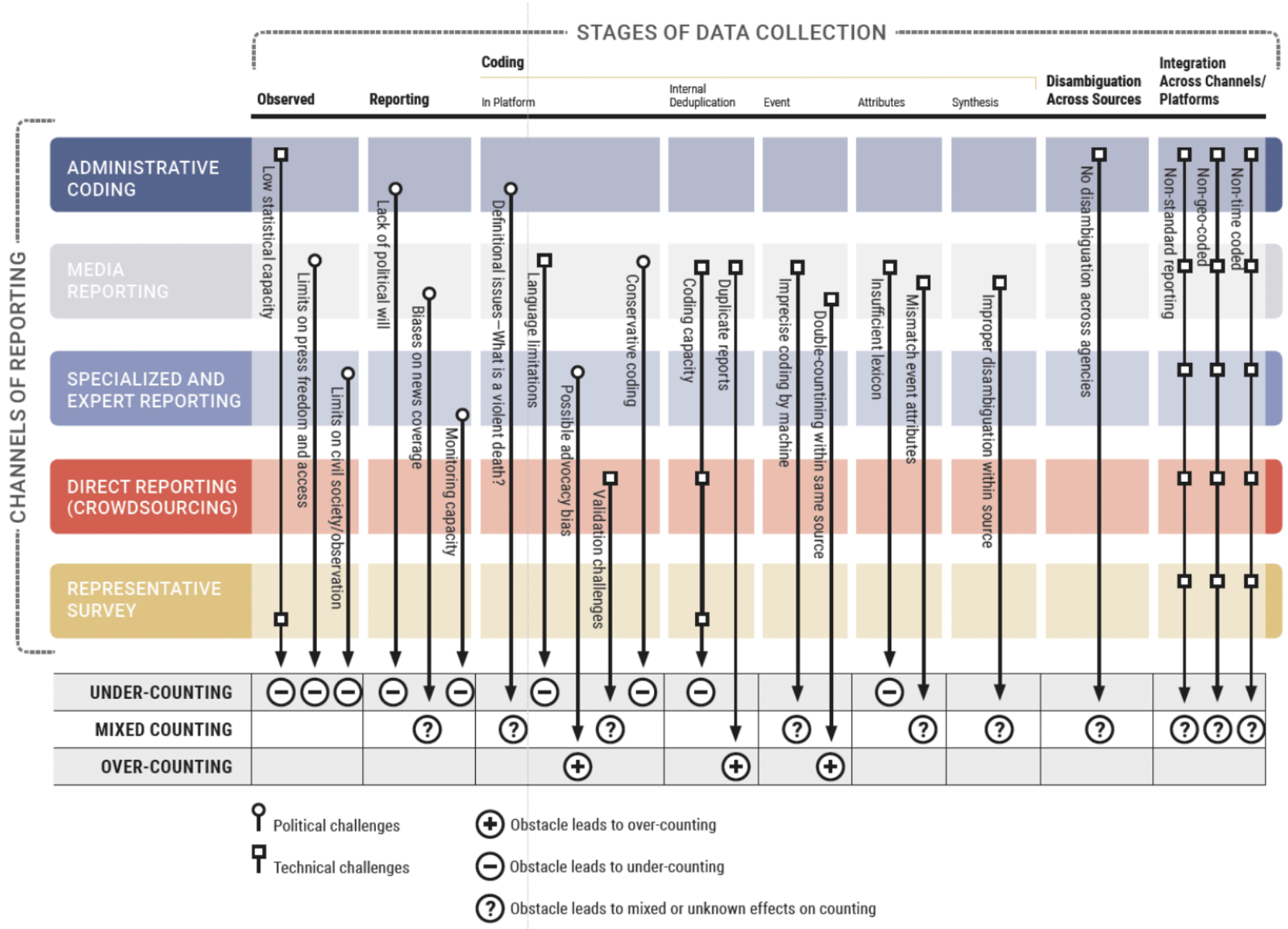

Thank you for sharing this conceptual graph, which inspired the development of Figure 1 in the revised manuscript. This new figure provides an overview of the data generation processes underlying text-based and satellite-derived data, and how these processes give rise to distinct strengths and inherent limitations. Given the word limit of the Analysis format, we keep the discussion of data generation processes and their limitations in the main text concise; the figure allows us to convey their complexity and to show how the resulting limitations can only be addressed through data integration. Complementing this schematic overview in Figure 1, the Supplementary Information provides a more detailed discussion of the data generation processes and related limitations.

I was really surprised there is no data now. I understand this is the steer of the editor. But if this data and analysis does not exist then at the heart of the article and the “ways forward” it should be at least mentioned that we do not have panel data that would allow us understand how satellite features like destruction data could complement other data. I was under the impression that someone must have generated destruction panel data from a war zone like Gaza or Ukraine with all the many ongoing projects in this area. The fact that a large team like this is not able to muster a single a dataset means that there is a very clear identifiable gap that should be mentioned. Putting it starkly: **we currently have no destruction data that could be merged with the news event data**

to make it possible to analyze how destruction data can add to the analysis of conflict. In section 6 the team goes into ethical issues and datasets being specific to one context. But the elephant in the room is clearly that no data can be deployed to analyze destruction over time and locations. So, the way forward is to generate data like this. I personally find this very motivating.

Thank you for this encouraging comment. It highlights a central tension we seek to address: demonstrating how data integration can move the field forward while operating in an emerging research area where many approaches remain proof-of-concepts, often fine-tuned for single contexts, with no available cross-country, analysis-ready panel datasets. Aligning text-based conflict event data with satellite-derived war damage data also requires substantial data processing to reconcile different spatial and temporal resolutions and to ensure conceptual compatibility, which we were unable to do under the old manuscript format.

The new manuscript format allows us to address the central concerns you raised head-on. In the main text, we show how integrating heterogeneous data linked by common time and location enables analyses of spatial and temporal dynamics that cannot be captured by any single data source alone. We provide concrete illustrations of how reliance on one data type can obscure temporal variation (Myanmar) or mask important differences across cities (Ukraine). These insights are novel, substantively highly relevant empirical contributions, which in our view convincingly demonstrate the value added and necessity of considering satellite-derived data in the study of violent conflicts. In the methods section, we then discuss the key trade-offs involved in aggregating and disaggregating data to a shared unit of analysis.

Navigating between promise and feasibility, we draw on algorithmically derived war-damage data for Ukraine and manually annotated data from UNOSAT, supplemented with automatically derived fire data, for Myanmar. This combination allows us to demonstrate pathways to integration and its analytical benefits across diverse settings. While the Ukraine example already involves quantitative analysis, we discuss issues of scalability for the Myanmar case in the Supplementary Information, and explicitly note the broader gap in developing generalizable approaches to collect data on a wide range of conflict contexts as a key priority for future research. These examples thus at the same time clearly demonstrate the potential of the kind of integrated analysis we advocate for while also emphasizing the need to prioritize methodological and substantive research in this area.

Referee #2:

The paper looks at how satellite data can be integrated with text-based conflict event datasets and presents a four-level framework of complementary use, triangulation, enrichment, and fusion. The topic is important and timely, and the manuscript covers a wide range of methods in a clear way. However, the overall contribution is limited. The first three levels of the framework are not new. Similar ideas have already been published, for example in Donnay et al. (2019, Journal of Conflict Resolution 63:1337–1364), which explained how conflict datasets can be combined by using complementary coverage, cross-validating overlaps, and adding unique attributes from different sources. The fourth level, fusion, goes a little further, but here it is described only in general terms without concrete evidence or examples. For a Perspective article in Nature, which should provide a distinctive and forward-looking argument, this overlap with existing work and the lack of originality is a serious weakness.

We agree that the previous version of the manuscript did not sufficiently clarify the paper's core contribution. As you note, elements of the original framework overlapped with prior work on integrating text-based conflict event data, including Donnay et al. (2019).

In the revised manuscript, we address this concern directly. Our core contribution is to propose concrete pathways of integrating text-based and satellite-derived data along three levels of integration, demonstrate how these pathways can be implemented in practice, and show the analytical opportunities and insights they enable. While existing approaches focus on integration across datasets of the same type—leveraging overlapping coverage, triangulation, or the addition of attributes within text-based sources—our contribution centers on integration across fundamentally different modalities. This poses distinct methodological challenges, but also enables qualitatively different forms of inference because the underlying information is orthogonal.

To make this distinction clearer, we revised both the conceptual framing and the structure of the framework. We now explicitly situate our approach relative to Donnay et al. (2019) in the main text, clarifying which elements build on existing ideas and which extend beyond them. We also removed the framework level focused on complementarity (co-use), which has been addressed elsewhere, and instead now focus on three approaches that require explicit linking across data types.

We further strengthened the manuscript by empirically illustrating these forms of integration and by expanding the conceptual discussion. The revised Methods section

details the concrete analytical decisions required to integrate text-based and satellite-derived data, including trade-offs related to spatial and temporal resolution and how we navigated these trade-offs in our examples.

We also deepened the discussion of the most advanced level of integration—multi-modal data fusion—by anchoring it more clearly in the specific challenges of conflict research and by outlining a concrete path forward, including the benefits and challenges this approach entails. A new conceptual figure visually clarifies these ideas. Multi-modal data fusion represents the next research frontier in our field. Unlike the preceding approaches, it cannot yet be demonstrated through empirical applications. We therefore include a text-based example that builds on the Myanmar case to illustrate the types of patterns such fusion could reveal when drawing on both text and satellite data.

The paper also suffers from weak empirical support. The case studies on Ukraine and Gaza are descriptive and do not provide systematic validation or quantitative results. There is no demonstration that the proposed framework actually improves reliability, accuracy, or scope of conflict monitoring. The methodological points are reasonable, but they remain at the level of categorizing approaches rather than showing new insights or paradigm shifts. The work reads more like a structured review than a Perspective.

We agree that the lack of empirical illustrations in the previous version limited the paper's ability to demonstrate the practical value of the proposed framework, a limitation largely driven by the constraints of the Perspective format. We therefore, in line with your feedback and following the advice of the editors, resubmit the manuscript as an Analysis, which allows us to empirically demonstrate different approaches to data integration.

In the revised manuscript, we remove the earlier examples from Ukraine and Gaza, which were descriptive and drawn from existing studies, and replace them with tailored, original empirical illustrations from Ukraine and Myanmar. These cases are used to show how the proposed framework generates distinct insights using different approaches to integration. Specifically, we illustrate how spatial refinement of text-based data can yield new analytical insights (Myanmar), how adding contextual information from texts to satellite-derived data can improve our understanding of targeting decisions (Ukraine), and how linking text-based and satellite-derived sources through a shared unit of analysis reveals new insights into spatial and temporal variation in violence (Ukraine and Myanmar).

We have also streamlined the manuscript to reduce review-style content in the main text. The main text now provides analytical synthesis that directly supports the paper's central argument, while material that primarily summarizes existing approaches has been moved to the Supplementary Information.

The conclusions are also not well supported. The article repeats the promise of multimodal integration but does not show how it would be implemented in practice or why it would transform conflict research in a fundamental way. Important issues such as error rates, uncertainties, validation challenges, and ethical risks are mentioned only briefly. The manuscript would need much stronger evidence and clearer vision to reach the level required for Nature.

Thank you for your assessment and for pointing out the elements that were underdeveloped. We fully agree that the conclusions were not well supported in the previous version of the manuscript, and addressed your concerns in three main ways.

First, we added a dedicated section on the fundamentally different data-generation processes of text-based and satellite-derived data, and discuss how these processes give rise to distinct strengths and inherent limitations (Section 2.3). This discussion is supported by a conceptual overview in Figure 1, with a more detailed treatment of the processes and associated sources of error and uncertainty provided in the Supplementary Information (Appendix C).

Second, we discuss ethical risks more explicitly in Section 3.3 (Multi-modal data fusion). While more narrow applications allow researchers to control training data and impose targeted limitations, the versatile nature of foundation models pose a more challenging setting. Moreover, advances in generative AI now make it possible to create realist synthetic satellite imagery. We discuss the implications of these challenges and highlight key mechanisms researchers should employ to deal with them.

Third, we rewrote the concluding section (Section 4, Way forward) to articulate two concrete priorities that provide a clear vision for how this research agenda can be implemented and scaled. Combined with the new empirical illustrations and more explicit discussion of limitations, uncertainties, and ethical considerations, these revisions strengthen both the evidentiary basis and the forward-looking contribution of the manuscript.

In summary, this is a careful overview of current methods, but it represents methodological improvements rather than paradigm-changing ideas. It lacks originality, rests on insufficient evidence, and does not deliver the strong forward-looking vision

expected from a Nature Perspective. It could be valuable as a review article in a specialist journal, but in its current form it does not meet the standards of novelty and impact for Nature.

We acknowledge that the previous version of the manuscript leaned too heavily on review-style content, lacked empirical evidence, and that the pathways for our forward-looking research agenda were not sufficiently developed. We are confident that the current version addresses these concerns by presenting a clear, evidence-based priority-setting advance. It lays out concrete pathways to data integration, provides empirical illustrations of their implementation, and demonstrates the unique insights this approach can generate—for example, revealing stark differences in targeting patterns between Russian and Ukrainian forces that remain hidden when text-based and satellite-derived data are used in isolation.

Revision Memo

Dear Editors and Reviewers,

Thank you for your thoughtful comments on our earlier manuscript and for the opportunity to submit a revised version. Please find the new manuscript attached.

We agree that empirical illustrations are essential to demonstrate how the integration of text-based and satellite-derived data can be implemented in practice. To enable such illustrations, we are resubmitting the manuscript as an *Analysis*, which is a more natural fit for our core contributions: outlining concrete pathways to data integration, demonstrating their practical application, and highlighting the novel analytical opportunities they enable.

The article follows a deductive structure: we first motivate the need for data integration by highlighting the inherent limitations of text-based and satellite-derived data when used in isolation. We then outline concrete pathways along three levels of integration and empirically illustrate each alongside the insights gained through such integration. This empirical, analytical lens is deliberate, demonstrating on a practical level the unique potential of using satellite-derived data in the study of violent conflicts.

Our case studies—Ukraine and Myanmar—showcase the distinct and novel insights data integration can generate in conflicts with different information environments: Ukraine as a data-rich context and Myanmar as a data-poor one. Our third level of integration, multi-modal data fusion, is explicitly forward-looking and reflects the next research frontier; we therefore limit its empirical illustration to a text-based example.

Given the addition of empirical illustrations and the shorter word limit of the new format, we now present only the core arguments and findings in the main text, moving extended discussions—such as a more detailed overview of the state of the field—into the supplementary information.

We believe these revisions clarify the paper's contribution and offer a way forward for advancing conflict research beyond a fatality-centric focus toward a multi-modal, multi-dimensional approach to studying violence in armed conflict. Your feedback has been essential in helping us to reshape and strengthen this contribution.

Please find our detailed responses to individual editor and reviewer comments below.

Editor comments:

Dear Dr Sticher,

I apologize for the delay in sending our decision. Your manuscript, "Advancing conflict research and response through satellite-derived data", has now been seen by two of the original referees, whose comments are attached below. In the light of their advice we have decided that we cannot offer to publish the manuscript in Nature in its current form but we are open to a new version that addresses our points below.

While the referees find your work of some interest, they raise concerns about the novelty of the approach and its potential to be executed without some form of illustration or validation. The other two referees became unresponsive despite our many chasers, so we had to proceed with the advice we got. We feel that these reservations are sufficiently important as to preclude publication of the present study in Nature, and we are accordingly closing the file as a Perspective. This is a very specific format that, as we've discussed, does not allow for data analysis. However, given that the referees keep raising this point in this round, we believe that the proposal would benefit from a recategorization into an Analysis. Analyses are priority-setting evidence advances, which means that there is a proposal with data (however preliminary) that shows how it's done — and in that case, this would address the referee concerns with the illustration and will also provide you with some flexibility of what you'd like to show. I would be happy to schedule a call where we can discuss these points if you prefer.

Should you be interested in following this route and further work would allow you to address these concerns, we would be happy to look at a revised manuscript (unless, of course, something similar has by then been accepted at Nature or appeared elsewhere). At such a time, you can request that the manuscript file be reopened for resubmission by simply sending me an email to that effect (stating manuscript number). In the case of eventual publication, the received date would be that of the revised paper. We would contact the some two referees that provided comments but we will have to look for others with overlapping experience with R3 and R4 as we did not hear back from them.

I am sorry that we cannot respond more positively in this occasion, and we hope that you find our referees' comments helpful.

Thank you for your assessment, the suggestion to recategorize the manuscript as an *Analysis*, and the clear guidance on how to proceed. We agree that the Analysis format

is better suited to highlighting the paper's core contribution. Adopting it and adding empirical illustrations required a substantial reworking of the manuscript, but this process allowed us to make our approach and analytical framework more concrete and transparent. We believe these additions sharpen the argument and more clearly demonstrate how the proposed approach can be implemented in practice.

Referee #1:

The article has improved dramatically. I congratulate the authors.

However, I am still concerned about the fact that the conceptual framework is somewhat superficial. It has no formal elements and does not talk about the data generating process in detail. Reading the guidelines by the editor I could see that no data analysis is allowed in the format here. This closes a big potential avenue to illustrate the points of the paper and give them more depth (see below). So, in what follows I will try to bounce some ideas. I know this is not great refereeing in the sense that I am asking a lot but please take the following as inspiration and an attempt for shifting the debate into more concrete territory to clarify my point from the first review.

Thank you for your overall assessment and for engaging so carefully with the manuscript. With the recategorization of the paper as an Analysis, we are now able to illustrate our arguments empirically and develop them in greater depth. We have also included a new section on data generation processes, alongside a new conceptual figure, as outlined below.

Perhaps my previous report was too vague but I think we are missing a map and this is what I would ask of a perspective article. Some ideas, please don't take them literally:

1) What are all the measurable dimensions of conflict we could care about and how could they relate to the conflict process? Then after the where are satellites adding and what should be the priority? Some ideas: tents/temporary housing, refugee trails, cars, transport trucks, road destruction, building destruction, vegetation destruction, fires, rubble, night light, market activity, military equipment, weapon craters.

Thank you for this concrete suggestion. We agree that the previous version of the manuscript did not sufficiently clarify the breadth of phenomena that satellite-derived data could, in principle, capture, nor our rationale for prioritizing specific dimensions (damage to the built and natural environment). We address this comment in two places in the revised manuscript.

First, in the main text, we open subsection 2.2 by discussing the wide range of data relevant to conflict-related violence, conceptually distinguishing between three aspects: (1) data on actors and instruments of violence (for example, troop movements or weapon systems), (2) data capturing the direct effects of violence (such as destruction of the built and natural environment), and (3) data on indirect effects (such as declines in agricultural production or market activity). We then explain why we see the greatest potential—and therefore set our priority—in collecting data on the second aspect, the direct effects of violence.

Second, building on this overview, we expand the discussion of these three aspects in the supplementary information (Section A, Data on three aspects of violence). While this section is not intended to be exhaustive, it provides concrete examples of the types of data each aspect may entail.

In addition, Section B of the supplementary information offers a more detailed discussion of satellite-derived data capturing material damage (Section B.1, Approaches to capture war damage) and violence against people (Section B.2, Capturing the human toll).

> 2) Combine this perhaps with a discussion on dimensions such as: importance for policy, importance for research, measurability with satellites, comparative advantage over text to derive a score of what could be a priority going forward.

This is a great suggestion but given the new article format as an Analysis we can move beyond a conceptual discussion and conduct empirical analyses based on existing data. We have therefore shifted the focus of the second part of the manuscript to demonstrating how data integration can be implemented in practice, outlining concrete pathways across three levels of integration. This approach also enables us to empirically illustrate the analytical opportunities and insights that data integration can generate, rather than assessing the importance of individual dimensions for research and policy in the abstract. This new empirical focus of the paper allows us to address the points you raised, but in a more concrete, hands-on empirical manner, which has significantly strengthened the contribution.

3) I paste the Global Registry of Violent Deaths (GReVD) conceptualization of how different violence measurement types could interact to illustrate how detailed others in this sector have approached similar topics. I know this is not a perfect fit as the goal here was something else. But perhaps this inspires the authors to more.

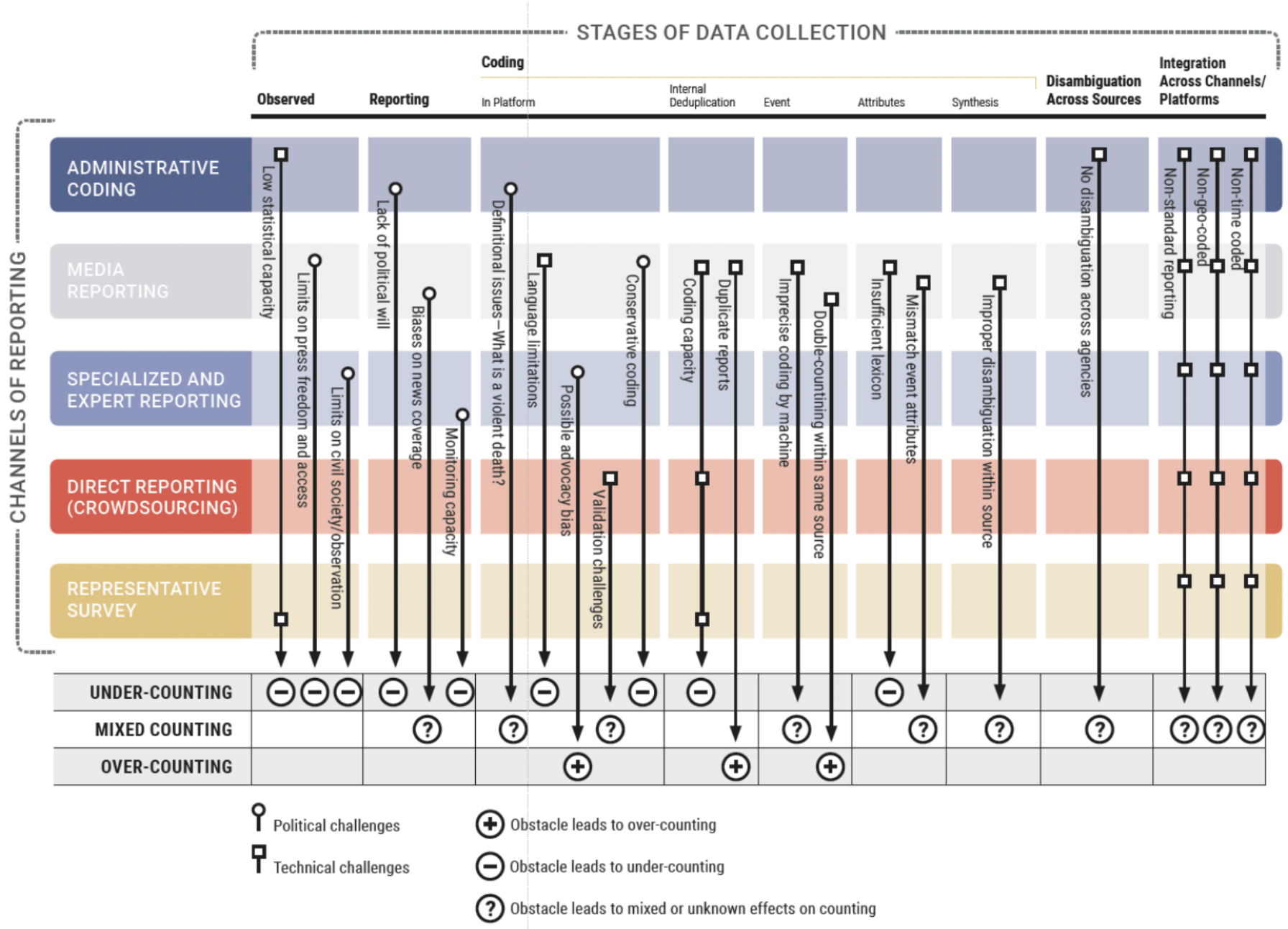

Thank you for sharing this conceptual graph, which inspired the development of Figure 1 in the revised manuscript. This new figure provides an overview of the data generation processes underlying text-based and satellite-derived data, and how these processes give rise to distinct strengths and inherent limitations. Given the word limit of the Analysis format, we keep the discussion of data generation processes and their limitations in the main text concise; the figure allows us to convey their complexity and to show how the resulting limitations can only be addressed through data integration. Complementing this schematic overview in Figure 1, the Supplementary Information provides a more detailed discussion of the data generation processes and related limitations.

I was really surprised there is no data now. I understand this is the steer of the editor. But if this data and analysis does not exist then at the heart of the article and the "ways forward" it should be at least mentioned that we do not have panel data that would allow us understand how satellite features like destruction data could complement other data. I was under the impression that someone must have generated destruction panel data from a war zone like Gaza or Ukraine with all the many ongoing projects in this area. The fact that a large team like this is not able to muster a single a dataset means that there is a very clear identifiable gap that should be mentioned. Putting it starkly: **we currently have no destruction data that could be merged with the news event data**

to make it possible to analyze how destruction data can add to the analysis of conflict. In section 6 the team goes into ethical issues and datasets being specific to one context. But the elephant in the room is clearly that no data can be deployed to analyze destruction over time and locations. So, the way forward is to generate data like this. I personally find this very motivating.

Thank you for this encouraging comment. It highlights a central tension we seek to address: demonstrating how data integration can move the field forward while operating in an emerging research area where many approaches remain proof-of-concepts, often fine-tuned for single contexts, with no available cross-country, analysis-ready panel datasets. Aligning text-based conflict event data with satellite-derived war damage data also requires substantial data processing to reconcile different spatial and temporal resolutions and to ensure conceptual compatibility, which we were unable to do under the old manuscript format.

The new manuscript format allows us to address the central concerns you raised head-on. In the main text, we show how integrating heterogeneous data linked by common time and location enables analyses of spatial and temporal dynamics that cannot be captured by any single data source alone. We provide concrete illustrations of how reliance on one data type can obscure temporal variation (Myanmar) or mask important differences across cities (Ukraine). These insights are novel, substantively highly relevant empirical contributions, which in our view convincingly demonstrate the value added and necessity of considering satellite-derived data in the study of violent conflicts. In the methods section, we then discuss the key trade-offs involved in aggregating and disaggregating data to a shared unit of analysis.

Navigating between promise and feasibility, we draw on algorithmically derived war-damage data for Ukraine and manually annotated data from UNOSAT, supplemented with automatically derived fire data, for Myanmar. This combination allows us to demonstrate pathways to integration and its analytical benefits across diverse settings. While the Ukraine example already involves quantitative analysis, we discuss issues of scalability for the Myanmar case in the Supplementary Information, and explicitly note the broader gap in developing generalizable approaches to collect data on a wide range of conflict contexts as a key priority for future research. These examples thus at the same time clearly demonstrate the potential of the kind of integrated analysis we advocate for while also emphasizing the need to prioritize methodological and substantive research in this area.

Referee #2:

The paper looks at how satellite data can be integrated with text-based conflict event datasets and presents a four-level framework of complementary use, triangulation, enrichment, and fusion. The topic is important and timely, and the manuscript covers a wide range of methods in a clear way. However, the overall contribution is limited. The first three levels of the framework are not new. Similar ideas have already been published, for example in Donnay et al. (2019, Journal of Conflict Resolution 63:1337–1364), which explained how conflict datasets can be combined by using complementary coverage, cross-validating overlaps, and adding unique attributes from different sources. The fourth level, fusion, goes a little further, but here it is described only in general terms without concrete evidence or examples. For a Perspective article in Nature, which should provide a distinctive and forward-looking argument, this overlap with existing work and the lack of originality is a serious weakness.

We agree that the previous version of the manuscript did not sufficiently clarify the paper's core contribution. As you note, elements of the original framework overlapped with prior work on integrating text-based conflict event data, including Donnay et al. (2019).

In the revised manuscript, we address this concern directly. Our core contribution is to propose concrete pathways of integrating text-based and satellite-derived data along three levels of integration, demonstrate how these pathways can be implemented in practice, and show the analytical opportunities and insights they enable. While existing approaches focus on integration across datasets of the same type—leveraging overlapping coverage, triangulation, or the addition of attributes within text-based sources—our contribution centers on integration across fundamentally different modalities. This poses distinct methodological challenges, but also enables qualitatively different forms of inference because the underlying information is orthogonal.

To make this distinction clearer, we revised both the conceptual framing and the structure of the framework. We now explicitly situate our approach relative to Donnay et al. (2019) in the main text, clarifying which elements build on existing ideas and which extend beyond them. We also removed the framework level focused on complementarity (co-use), which has been addressed elsewhere, and instead now focus on three approaches that require explicit linking across data types.

We further strengthened the manuscript by empirically illustrating these forms of integration and by expanding the conceptual discussion. The revised Methods section

details the concrete analytical decisions required to integrate text-based and satellite-derived data, including trade-offs related to spatial and temporal resolution and how we navigated these trade-offs in our examples.

We also deepened the discussion of the most advanced level of integration—multi-modal data fusion—by anchoring it more clearly in the specific challenges of conflict research and by outlining a concrete path forward, including the benefits and challenges this approach entails. A new conceptual figure visually clarifies these ideas. Multi-modal data fusion represents the next research frontier in our field. Unlike the preceding approaches, it cannot yet be demonstrated through empirical applications. We therefore include a text-based example that builds on the Myanmar case to illustrate the types of patterns such fusion could reveal when drawing on both text and satellite data.

The paper also suffers from weak empirical support. The case studies on Ukraine and Gaza are descriptive and do not provide systematic validation or quantitative results. There is no demonstration that the proposed framework actually improves reliability, accuracy, or scope of conflict monitoring. The methodological points are reasonable, but they remain at the level of categorizing approaches rather than showing new insights or paradigm shifts. The work reads more like a structured review than a Perspective.

We agree that the lack of empirical illustrations in the previous version limited the paper's ability to demonstrate the practical value of the proposed framework, a limitation largely driven by the constraints of the Perspective format. We therefore, in line with your feedback and following the advice of the editors, resubmit the manuscript as an Analysis, which allows us to empirically demonstrate different approaches to data integration.

In the revised manuscript, we remove the earlier examples from Ukraine and Gaza, which were descriptive and drawn from existing studies, and replace them with tailored, original empirical illustrations from Ukraine and Myanmar. These cases are used to show how the proposed framework generates distinct insights using different approaches to integration. Specifically, we illustrate how spatial refinement of text-based data can yield new analytical insights (Myanmar), how adding contextual information from texts to satellite-derived data can improve our understanding of targeting decisions (Ukraine), and how linking text-based and satellite-derived sources through a shared unit of analysis reveals new insights into spatial and temporal variation in violence (Ukraine and Myanmar).

We have also streamlined the manuscript to reduce review-style content in the main text. The main text now provides analytical synthesis that directly supports the paper's central argument, while material that primarily summarizes existing approaches has been moved to the Supplementary Information.

The conclusions are also not well supported. The article repeats the promise of multimodal integration but does not show how it would be implemented in practice or why it would transform conflict research in a fundamental way. Important issues such as error rates, uncertainties, validation challenges, and ethical risks are mentioned only briefly. The manuscript would need much stronger evidence and clearer vision to reach the level required for Nature.

Thank you for your assessment and for pointing out the elements that were underdeveloped. We fully agree that the conclusions were not well supported in the previous version of the manuscript, and addressed your concerns in three main ways.

First, we added a dedicated section on the fundamentally different data-generation processes of text-based and satellite-derived data, and discuss how these processes give rise to distinct strengths and inherent limitations (Section 2.3). This discussion is supported by a conceptual overview in Figure 1, with a more detailed treatment of the processes and associated sources of error and uncertainty provided in the Supplementary Information (Appendix C).

Second, we discuss ethical risks more explicitly in Section 3.3 (Multi-modal data fusion). While more narrow applications allow researchers to control training data and impose targeted limitations, the versatile nature of foundation models pose a more challenging setting. Moreover, advances in generative AI now make it possible to create realist synthetic satellite imagery. We discuss the implications of these challenges and highlight key mechanisms researchers should employ to deal with them.

Third, we rewrote the concluding section (Section 4, Way forward) to articulate two concrete priorities that provide a clear vision for how this research agenda can be implemented and scaled. Combined with the new empirical illustrations and more explicit discussion of limitations, uncertainties, and ethical considerations, these revisions strengthen both the evidentiary basis and the forward-looking contribution of the manuscript.

In summary, this is a careful overview of current methods, but it represents methodological improvements rather than paradigm-changing ideas. It lacks originality, rests on insufficient evidence, and does not deliver the strong forward-looking vision

expected from a Nature Perspective. It could be valuable as a review article in a specialist journal, but in its current form it does not meet the standards of novelty and impact for Nature.

We acknowledge that the previous version of the manuscript leaned too heavily on review-style content, lacked empirical evidence, and that the pathways for our forward-looking research agenda were not sufficiently developed. We are confident that the current version addresses these concerns by presenting a clear, evidence-based priority-setting advance. It lays out concrete pathways to data integration, provides empirical illustrations of their implementation, and demonstrates the unique insights this approach can generate—for example, revealing stark differences in targeting patterns between Russian and Ukrainian forces that remain hidden when text-based and satellite-derived data are used in isolation.

Revision Memo

Dear editors, dear reviewers:

Thank you for the constructive comments and for the invitation to submit a revised version of this Analysis. Please find the new manuscript attached. The Analysis offers a concrete pathway to broaden our understanding of conflict-related violence through the integration of text-based conflict event data with satellite-derived data.

We have addressed each comment in detail in the responses below. Two changes shape the revision most substantially. First, we elevate the case studies through a conceptual framework that links the data generation processes of text-based and satellite-derived data to our three proposed approaches to integration, showing how each approach addresses a specific gap in single-source data. Building on this, we distinguish between information-rich and information-poor environments and show how the gaps that satellite-derived data bridge, and the analytical weight they carry, shift across these environments.

Second, we treat text-based and satellite-derived data more symmetrically throughout. We now frame text-based data around the source-level dependence on eyewitnesses being present, and willing and able to share information, which mirrors the source-level dependence on sensors for satellite-derived data. Figure 1 reflects this symmetry, and a new discussion in the main text distinguishes three main types of limitations of satellite-derived data and clarifies which can be mitigated and which are inherent to the source.

With these revisions, the Analysis sets a clear research agenda by providing both a conceptual framework and concrete applications for how the integration of text-based and satellite-derived data can advance our understanding of armed conflict. Your feedback has been essential in helping us sharpen this contribution.

Editor comments:

Dear Dr Sticher,

Thank you for your patience while we received our reviewer reports. Your manuscript, "Advancing conflict research and response through satellite-derived data", has now been seen by three referees (two from the previous rounds and a new R5 as R2 and R4 were unresponsive this round). You will see from their comments below that while they find your work of interest, some important points are raised. We are interested in the possibility of publishing your study in Nature, but would like to consider your response to these concerns in the form of a revised manuscript before we make a final decision on publication.

Thank you for the invitation to submit a revised version of the manuscript and for the constructive comments.

We therefore invite you to revise your manuscript taking into account the following points:

-The conceptual thread that binds the cases and the argument together more strongly.

We have revised the manuscript to build a clear conceptual thread that elevates the case studies and binds the data generation discussion to the data integration framework. We do this in two ways.

First, we clarify the relationship between the limitations of single-source data and the three approaches to integration we propose: each approach bridges a specific gap in single-source data. Second, we distinguish between information-rich and information-poor environments and show how the gaps that satellite-derived data bridge, and the analytical weight they carry, shift across these environments. We discuss these revisions in detail in response to R1’s final comment.

-A reorganization of Figure 1 in line with R1 and R5's comments.

We have reorganized Figure 1 in line with the comments by R1 and R5. We created symmetry between the discussion of the two data types by expanding the source-level description for satellite-derived data, so that each source box now describes how a phenomenon must register, through an eyewitness or a sensor, before it can enter the data generation process. We added labels for the three stages of the process (source, processing, and output), to clarify where each constraint applies, and refined several other labels. We also revised the accompanying text to discuss three main types of limitations of satellite-derived data. Our revisions are detailed in the responses to R1's and R5's comments on Figure 1.

-The discussion regarding survey data (R5), self-selection biases (R1) and measurement errors (R3).

We have addressed each of these points in the revised manuscript, as discussed in our responses to R1, R3, and R5 below. The revisions include a clearer scoping of our Analysis to systematic, retrospective data collection across cases and time, a more direct treatment of how attention-driven reporting and eyewitness availability shape what enters text-based datasets, and a new discussion of three types of limitations of satellite-derived data, including how their measurement errors relate to those of text-based data.

-The rest of the reviewer comments.

We address all reviewer comments, as detailed in our responses below.

Referee #1

Thank you for taking my comments into account. I can clearly see the contribution now and I think this will be a very useful paper to bring the debate forward. My comments are only minor now (all circle around the same issue) given the paper has very clear strengths now.

First, I think there is substantial work on capturing non-lethal events with text which are not consistently mentioned. ACLED here is leading with riots and sexual violence but projects like GDELT, ICEWS and POLCAT all show that event data generated from text can be quite rich. The key constraint is indeed the focus on news events as a driver of what becomes data and here is where the satellite data adds tremendously. I just make this point to warn of looking too dismissive of the text data in the beginning of the paper – this data is very rich and is used to track all kinds of events "below" fatalities. The point really is that news reporting and even eyewitness reports that are reported to NGOs will focus on different types of events than building and crop destruction which could take place exactly where no human other than the perpetrators are present. This point is made very nicely in the case studies but has not made it to the earlier part of the article I feel.

Thank you for your positive assessment and for your helpful comments throughout this review process.

We appreciate your observation about existing work on capturing non-lethal events, and agree that the earlier framing sections did not acknowledge this consistently. We also see how those sections could read as dismissive of text-based data. This was not our intention; several of us regularly use text-based data, and some have contributed to producing it. We think your suggestion to frame the limitation around news reporting and the presence of eyewitnesses is the right way to sharpen the argument. We have revised the manuscript to address your comment in three main ways.

First, we have rewritten Section 2.1, which deals specifically with text-based data. We now state more clearly that what enters a dataset is based on what gets reported, so that attention-driven news reporting shapes which event types are captured. As you note, this news-driven character of reporting is the core constraint. We cite research showing that non-lethal events are less likely to be reported, and that when they are, they are often recorded only in aggregate terms (e.g. that crop destruction occurred in a given region or by a given actor, but not precisely where).

As part of the same revision, we have added examples of data collection efforts that extend beyond fatalities, including the references you suggested. We also mention emerging approaches and discuss what they may help address. At the same time, we state that, because these data collection efforts still depend on human reporting, they do not remove the underlying limitation, which is most pronounced for non-lethal events. For the same reason, we retain the statement that much conflict research remains fatality-focused. Many conflict researchers continue to rely on fatalities as their main, and often sole, indicator of violence intensity,

especially for subnational analysis across conflict contexts, because non-lethal events are far less consistently captured.

Second, we now state more directly that reporting depends on eyewitnesses who are present, and willing and able to share what they observe. This is a core reason why satellite-derived data offers a complementary perspective, because it does not depend on such presence, nor on the willingness and ability to share information by those on the ground. In addition to clarifying this in the main text, we have also integrated this point in Figure 1 (see comment below).

Third, we now discuss three main types of limitations of satellite-derived data explicitly, rather than only listing limitations in Figure 1 and the supplementary information. This does not relate directly to non-lethal events in text-based data, but it reinforces that we do not privilege one data type over the other: both have distinct strengths and limitations, and integration is what allows the strengths of each to be used and the limitations addressed.

Second, this directly brings me to the data-generating process Figure. I think it has many nice elements but I don’t fully agree on how this is done. The first point in text analysis is: “Depends on political regime, media access, humanitarian presence, communications infrastructure, and perceived newsworthiness” whereas the satellite side the first point is “Events with physical manifestations”. This does not suggest a symmetry in the way the data-generating process is tracked and evaluated. Day and night cycles, seasonal changes in vegetation, technical machine learning problem with missing labels, cloud coverage and satellite angles are more similar to news bias and availability. Or should it be "eyewitnesses available" for the first point for text? In my opinion, the point here should really be thinking about how the data is captured through sensors and processed and compare this to how eyewitness reports reach the database of the researchers. The authors admit later that automated coding is lacking for most conflicts - precisely because the labels and technology are still lacking. This is currently reflected by a yellow box in Figure 1 that makes it look like a minor issue. But it is a massive bias. Figure 1 is valuable both as a vision – if we can improve destruction data further to reach fuller coverage – and as a description of reality with all the abovementioned biases. But it is not clear what it wants to be. It looks like depicting reality for text and like proposing a vision for destruction data.

Thank you for these observations, which identify a real problem with the previous version of Figure 1. You are right that it depicted the data generation process asymmetrically, especially at the source side. We also did not consistently discuss the limitations of satellite-derived limitations in the accompanying text, so together, this risked suggesting that satellite-derived data are less susceptible to limitations than text-based data. That is not our argument. Both data types have important limitations; these arise from different mechanisms, and addressing them is precisely what motivates data integration. We have revised the figure and the accompanying text accordingly.

First, we have created symmetry between the two sides by expanding the source-level description for satellite-derived data, which the previous version conceptualized too narrowly. The revised figure mirrors the structure used for text-based data. As you suggest, the source box for text-based data now centers on eyewitnesses: "Depends on eyewitnesses being present, willing, and able to share information; affected by factors such as political regime type, media access, and communications infrastructure." The corresponding box for satellite-derived data reads: "Depends on satellite viewing angle and resolution; affected by physical obstructions (e.g., vegetation, rooftops) and cloud and lighting conditions (for optical sensors)." With these revisions, each source box now describes how a phenomenon must register, through an eyewitness or a sensor, before it can enter the data generation process. Two boxes beneath the satellite source box specify constraints particular to commercial and open-access satellite data, since the choice between them currently involves important trade-offs that we detail in the supplementary information. We have also refined several other labels and added labels for the three stages of the data generation process, source, processing, and output, to clarify where each constraint applies.

Second, we agree that the previous version was ambiguous regarding whether to depict the reality or a vision. With our revisions, Figure 1 describes the data generation process as it stands today. The color coding adds a distinction within that reality: red marks limitations that are inherent to the source and cannot be overcome through innovation and technical improvement. Orange (previously yellow) marks limitations that are real and consequential but can, in principle, be reduced. We replaced the yellow boxes by orange boxes to avoid signaling that these limitations are minor (see also changes in accompanying text, next paragraph). For example, the scarcity of reference data, that models are limited to a few settings, and limited spatial resolution of open-access data today are major limitations. We classify them as orange because reference data can be expanded, models can be trained to generalize across settings, and open-access resolution may improve as new satellite missions and non-profit initiatives expand high-resolution coverage. Red limitations, by contrast, cannot be overcome through innovation and can only be addressed through data integration.

Third, the main text now addresses the limitations of satellite-derived data directly, rather than leaving them to the figure and supplementary information. We now distinguish three types: (1) limitations arising from data availability and researcher choices; (2) limitations arising because events are not equally detectable across forms of violence and settings; and (3) limitations inherent to the source, which no technical, conceptual, or organizational innovation can overcome. We give examples and implications for each, and discuss the main factors that lead to these limitations in more detail in the supplementary information (Section C.2). This also sets up the data integration section, where the specific gap a researcher needs to bridge determines which form of integration is appropriate (see our response to your final comment).

Third, my previous point can be seen in the case selection of Ukraine/Myanmar – was there always coverage on all time slices/locations? Or was there an satellite operator/purchase that was needed to get the images of the villages that were destroyed in Myanmar? What were the

underlying organizational biases in UNOSAT here? I really like the cases and they are powerful but I think they are clearly hand-picked and it is not clear from this what would happen with a systematic adoption/availability of this sort of data. I think Figure 1 could play a role for that – to evaluate what could be missing from satellites and how the two sets are truly complements. The displacement example, which is now even implemented (amazing) can really help. Eyewitness reports are scarce exactly where violence is most vicious. Displacement is most permanent where destruction is taking place. So, destruction data would really help us understand displacement data differently. News event data misses this because displacement is negatively correlated with eyewitnesses (mortality risk is a direct driver of data availability for text data).

Thank you for raising this. We agree that the previous version did not adequately address the limited availability of satellite-derived data. The revised manuscript gives this much more weight, in particular in the new discussion of the three types of limitations of satellite-derived data, outlined above. The first type directly raises the limited availability of reference data and how researcher choices about where to train and validate models shape what becomes detectable. As we now state, this is linked to international attention, but unlike the uneven availability of human reporting, it can be addressed through deliberate efforts to expand reference data and to train and validate models in underrepresented contexts. Several co-authors of this Analysis are currently involved in such efforts. We address case selection and the UNOSAT data generation process in more detail in response to your next comment.

We also appreciate the connection you draw between displacement and eyewitness presence. We now make this point explicitly in the manuscript, by adding this sentence: “In information-poor environments, satellite-derived data carry more of the analytical weight to begin with, and even more so when violence itself decreases reporting as populations are killed and flee.”

Turning towards the cases – I think this is a very strong part of the paper as it now stands. I really liked all the discussion here and I also went through the methodology and appendix section and learned a lot. This is great work. My only nag is that the lack of connection between the Figure 1 and this section. The discussion in the cases is very rich. The Myanmar case relies on hand coded data, Ukraine on a true panel dataset (!) of destruction data. These are very different things and it is not clear what data generating process underlies the UNOSAT manual coding – is this the universe of burned villages in this conflict? Could it be that news and eyewitness accounts played a role here? The authors themselves write towards the end of the article that only some countries are coded with destruction data. So here we seem to be still on a technological frontier. What drives whether destruction data is available? This is not discussed systematically but it is discussed for the text data.

Thank you for raising this important issue, which the revised manuscript addresses directly.

On the specific question about UNOSAT: manually annotated data of this kind are only indirectly linked to news or eyewitness reports of specific events. For Rakhine State, UNOSAT

systematically mapped the entire territory for the reported time period, not only villages reported as burned. The broader issue is that decisions about where to conduct such mappings are enabled by the availability of very high-resolution imagery and driven by demand, which in turn is linked to international attention. Reference data is usually best available for conflicts that receive sustained attention, such as Ukraine and Gaza, and often scarce or absent for underreported conflicts, precisely where satellite-derived data would be most valuable.

We are also more explicit about our case selection. We deliberately chose contrasting information environments, Ukraine as an information-rich setting and Myanmar as an information-poor setting, to show how data integration adds value across both. Different forms of integration are better suited to different environments. For Ukraine, we draw on automated war damage assessments (Figure 1, panel b, right path); for Myanmar, on manually annotated data (Figure 1, panel b, left path). We also use fire data, which is publicly available at high temporal resolution, for the improvement of spatial precision of text-based data at a more aggregate level (Figure 2, f-h). Ukraine and Myanmar were selected from a limited pool of available data of each type. There is, however, a clear path to expanding both: investing in reference data for underrepresented conflicts, and using that data to train and validate automated approaches for those same contexts.

This brings me to my final point. I think it is exactly in the interaction between conceptual thinking about the interactions between text and image, ordering the literature along these lines and also ordering the analysis part into this where I see the clearest way to contribute. The case studies need to be elevated with some sort of a framework that helps us to understand where satellite data adds. But at the moment the authors seem to inflate the availability and readiness of destruction data and do not systematically mention that most conflicts and most areas are not covered. What is the bias in this selection?

Thank you for this observation. You are right that in the previous version, the discussion of the data generation processes and the case studies were not conceptually connected, beyond the limitations highlighting the need for data integration. The revised manuscript builds a conceptual thread that binds the cases and the argument together.

First, we clarify the relationship between the limitations of single-source data and the three forms of integration. Improvement, enrichment, and fusion each bridge a different gap. Improvement addresses cases where one data type covers the violence of interest but lacks a specific feature, such as the spatial precision missing from text-based data or the contextual narrative missing from satellite-derived data. Enrichment is needed when no single source captures the full scope of relevant violence, as in campaigns that combine killings, destruction, and displacement. Fusion, the deepest level of integration, jointly models text and satellite data to infer phenomena that neither source captures directly. We introduce this in the abstract and develop it in the introductory paragraph of Section 3, and we return to it in the case examples to show not only how integration can be done but how it advances our understanding of armed conflict.

Second, we now distinguish between information-rich and information-poor environments, linking the case studies directly to the uneven availability of human reporting. The two cases we present illustrate data integration in contrasting information environments (Myanmar as an information-poor, Ukraine as an information-rich example). Both cases were selected from the limited pool of conflicts for which manually annotated or automated war damage data are currently available, a bias we address directly in our discussion of reference data availability above. Integration adds value across information environments, but the gaps that satellite-derived data bridge shift: in information-poor settings, where text-based data are sparse, satellite-derived data carry more of the analytical weight. This becomes even more pronounced when populations who could report on violence are killed or displaced. Some integration applications also require fine-grained text-based data that is only available in information-rich environments. Our Ukraine improvement example shows this, where fine-grained text-based data on territorial control enable contextual insights that satellite-derived data alone cannot provide. We introduce the distinction between information environments in the second paragraph of Section 3 and apply it through the case studies.

Referee #3

This article offers an important contribution to the study of armed conflict. The main point of the piece is to argue that satellite data offers a new and complementary data stream to existing text-based sources. This core argument is, overall, a success. The article is original, thoughtful, and high quality.

The authors could improvement the article in two ways.

First, for the "improvement" approach, can the authors clarify the assumption that killing and destruction occur in the same location? In the case of Ukraine, for example, it is unclear how to think about prior destruction that predates current day events. This also relates to measurement error, both off-the-shelf event data (from news stories) and satellite data are subject to measurement error. The combination of the two data streams produces a third category of error-- what does this look like, how does it amplify uncertainty when you think about the error together rather than each as categorically separate? Is there a dependency structure here that matters for inference? I.e., are the errors systemically correlated? (Low reporting environments correlated with poor satellite detection?)

Thank you for these comments. We agree that the previous version did not clearly specify the co-occurrence assumption, and we have addressed this in the revised manuscript. We now state the condition explicitly: "Because satellite-derived data are spatially explicit, we can use them to refine the location of text-based data, provided that the form of violence captured in the text-based data co-occurs with the form captured in the satellite-derived data (see Section C.2), such as killings that take place alongside dwelling destruction." In the Myanmar example, we refer back to this assumption and explain why we believe it holds in that specific case. In

Section C.2 of the supplementary information, we expand on the discussion and include examples where such an assumption would not hold.

On measurement error, we appreciate your suggestion to think about how combining data types can produce a distinct category of error. The revised manuscript is much more explicit about the limitations of satellite-derived data. We have reorganized Figure 1 (see responses to R1 and R5) to create symmetry between how we treat text-based and satellite-derived data, and we now distinguish three types of limitations of satellite-derived data in Section 2.2. Some mirror those of text-based data but stem from different mechanisms, which is itself relevant to your question about systematic correlation. We discuss which limitations can be overcome through technical or organizational innovation and which are inherent to the source, and what each implies for research. Where errors correlate, as we discuss for the urban bias, researchers need to account for this correlation. Automated war damage assessments are currently fine-tuned for information-rich environments because this is where we tend to have reference data for training and validation. This is a limitation that can be addressed through deliberate efforts to expand reference data and develop methods that generalize to underrepresented conflict contexts. We clarify these relationships in Section 2.2 and return to them in the conclusion.

Second, while the authors acknowledge case selection limitations in the 'way forward' section the mention is very brief. Perhaps in the supplemental materials there could be a table that suggests which cases these methods would excel in, which they would not, and why? Wouldn't the methods work less well for conflicts taking place in dense forests, with low tech violence, and/or a lot of clouds? It is notable that neither of the cases used in for illustration are in Africa or Latin America. I think there is a bit of a tendency of the authors to oversell the contribution.

Nevertheless, I commend the authors on a clear and novel approach.

Thank you for your observation and overall assessment. We agree that the limited pool of conflicts for which data are available and automated assessments are fine-tuned is a major limitation. The revised manuscript treats this as one of the three main types of limitations of satellite-derived data in Section 2.2, rather than only mentioning it briefly in the conclusion (see our previous response). Figure 1 now includes physical obstructions, such as vegetation and rooftops, and cloud and lighting conditions for optical sensors. We have also expanded the discussion of these factors in Section C.2 of the supplementary information. We considered adding a table summarizing these challenges, but opted instead to add practical examples from countries affected by specific challenges, such as forms of violence in Mexico and Central America that are hard to detect through satellite-derived data, or cloud cover in the Congo Basin.

Referee #5

The authors compare satellite imagery & methods to media-reported events for studying conflict. The manuscript is well-written and provides three ways to integrate the data to provide

a richer picture of conflicts. The authors provide novel examples for two of the integration methods and outline a path forward for the third. The approach should be of interest to many conflict researchers. The authors have done a good job bringing together data and methods to provide examples of what they are proposing. I have the following suggestions that should be addressed prior to publication:

The biggest weakness I see is that the authors do not mention survey methods or interviews or other boots-on-the-ground fieldwork as a way of measuring the effects of conflict. This could be addressed by constraining the scope of the manuscript to only consider 'remote' conflict studies, but I don't think this what the authors intended. Instead, I think it makes more sense to add a discussion about these other ways of measuring conflict.

Examples include work from researchers such as John O'Loughlin, Gerard Toal, and Kristen Bakke who regularly conduct large surveys with many 1000s of respondents in active or recent war zones. Door-to-door surveys are very effective, but for places too risky, phone surveys can be effective. Other large surveys such as Afrobarometer are regularly used to study conflict in Africa.

Thank you for these comments. You are right that the previous version did not engage with survey methods, interviews, and other fieldwork-based approaches to measuring the effects of conflict. These methods produce some of the most analytically rich data we have on conflict dynamics, and our framing was too narrow in not acknowledging this.

The revised manuscript addresses the omission in two places. First, in the introductory paragraph of Section 2, we clarify the scope of our Analysis: we acknowledge that fieldwork and surveys offer invaluable insights into various conflict dynamics, and clarify that we focus on techniques that enable systematic, retrospective data collection on violent events across cases and over long time spans. Even large, recurring surveys such as Afrobarometer measure respondent’s attitudes and experiences rather than collecting data on violent events across time and space. Second, in the last paragraph of Section 2.1, we argue that, while approaches that bypass media intermediaries may improve coverage of underreported forms of violence, they ultimately still depend on witnesses being present, and willing and able to share information about violence (see also comment on digital fieldwork infrastructures).

These other contributions to conflict are important since the manuscript concludes with a call for "a fuller picture of violence in armed conflict". Such a fuller picture cannot be achieved without talking with people on the ground. For instance, rape is a common weapon of war that rarely gets picked up in the kind of news media described in the manuscript. Other effects such as psychological trauma associated with witnessing violence or losing family/friends to the war are similarly not captured.

I'm not sure exactly where these kinds of less-visible effects from violence fit into Supp Info A. Seems like it is a direct human effect of conflict, but none of your data discussions address it. Perhaps the 3 categories identified need to be re-thought or explained more fully.

Adding in these dimensions & methods of data collection will likely require modifying Fig. 1a, or coming up with some sort of justification for why you are not including it as part of the study of conflict data generation. Or perhaps phone surveys would count as 'Remote sensors' on Fig. 1b, but that would really throw things off.

We agree that these approaches are essential to a fuller understanding of violence in armed conflict, and that the techniques we focus on are not well suited to capture phenomena such as rape and psychological trauma. Satellite-derived data cannot address these gaps.

We address your comments in three places. First, in the main manuscript, we now reference sexual violence as a form of violence that is underreported, when we discuss emerging approaches that bypass media intermediaries (see comment below).

Second, we revised the concluding paragraph to clarify that the integration of text-based and satellite-derived data moves quantitative conflict research, specifically, toward a fuller picture of violence.

Third, in Appendix A of the supplementary information, we clarify how these forms of violence map onto the three aspects of violence we identify. We treat sexual violence as a direct and psychological trauma as an indirect effect of violence, and we now explicitly state that the approaches presented in our Analysis are not equally suited to capture all forms (for example, sexual violence).

We have not changed the basic structure of Figure 1, but we updated the top box of panel a to clarify that text-based data depend on eyewitnesses being present, and willing and able to share information (see next comment). Although we do not classify survey data and interviews as text-based data, they share the same source-level constraint: they rely on human reporting, and the source limitations therefore mirror those of text-based data.

And even if you want to exclude data collected from surveys and interviews, there is also an emerging field of 'digital fieldwork' that should be incorporated as a way of studying conflict. Here are some recent relevant citations (I am not affiliated with either):
Limonier, K., & Audinet, M. (2025). From restricted to digital fieldwork: A renewed methodological framework for Russian studies after the full-scale invasion of Ukraine. Communist and Post-Communist Studies, 1–15. https://doi.org/10.1525/cpcs.2025.2474792
Sereda, V., & Mikheieva, O. (2025). How (not) to study a war-affected society: Challenges of knowledge production in Ukraine and elsewhere. Nationalities Papers, 1–20. https://doi.org/10.1017/nps.2024.56

Thank you for these references. The revised manuscript now explicitly engages with emerging approaches such as digital fieldwork infrastructures (last paragraph of Section 2.1). We argue that, because they bypass media intermediaries, they can help improve

coverage of underreported forms of violence such as sexual violence. At the same time, because of their reliance on human reporting, they are still subject to the source-level limitation that we discuss above.

We have integrated the Limonier & Audinet (2025) reference in the digital fieldwork discussion, alongside an article on crowdseeding. We do not cite Sereda & Mikheieva (2025) directly, but their argument resonates with the point we now make about data collection in conflict-affected contexts that depends on witnesses being present on the ground: limited access to large parts of the population, sampling challenges due to displacement, and risks to research participants.

A few more thoughts on Fig. 1 since it is ripe for picking at. Overall, I like the concept of the figure, though as I mention above, it is incomplete. For the Remote sensors side, I think it would be worth adding "Detection uncertainty" to the Manual annotation box since there are plenty of war effects that are not obvious to the human eye in the imagery. For instance, is that a bomb crater in the field? Is that structure damaged, or does it just have an oddly shaped/colored roof?

Thank you for this pertinent observation. This was an important omission in the previous version, and we have included this label. To highlight the different nature of uncertainty in manual versus automated approaches, we use distinct labels: “Detection uncertainty” for manual annotation and “Probabilistic detection” for automated approaches.

Also, still on the Remote sensors side, the Automated coding items seem to be specific to SAR data analysis. I guess I am thinking this based on "Underrepresentation of certain types of events and small objects". With computer vision and AI methods, I think we are at a point where it would be possible to automate the Manual annotation approach using optical satellite imagery. It might be expensive, but someone could use all high-res cloud free imagery over a large (global?!) conflict zone and have an AI annotate areas of damage. Indeed, I just made this suggestion to someone at a conference last week who had full access to Maxar/Vantor's high res imagery.

Thank you for this comment. We fully agree that any type of satellite data, including very high-resolution optical imagery, can serve as input for war damage assessments, and that AI-based annotation of very high-resolution optical imagery is in principle feasible at scale. There are also increasing efforts to combine different types of satellite data in multi-modal approaches.

In our conceptualization of Figure 1, AI-annotated war damage fits into panel b, right side, since we view it as a form of automated war damage assessment rather than manual annotation. The distinction between commercial and open-access data is meant to highlight the current challenges associated with each type, not to suggest that only open-access data can be used for automated analysis. The revised figure makes this clearer: the arrows from both boxes lead

together at the end of the source block, indicating that either can serve as input to the processing stage.

We also fully agree that future approaches can mitigate some current limitations. The revised manuscript engages this directly in Section 2.2, where we introduce three types of limitations of satellite-derived data and distinguish which can be mitigated through technical or organizational innovation and which are inherent to the source. Your example speaks to the second type of limitations we discuss: some features make certain forms of violence in certain settings harder to detect, but this can be addressed as methods and reference data improve.

Analysis Comments

At the end of Section 3.1, the authors claim that "Russian forces destroyed substantially more buildings when taking over territory than when Ukraine recaptured territory". But I don't see where this is normalized by area. Perhaps I am missing something, but does Fig. 3c just reflect that more territory was captured by Russia than by Ukraine? If it's just a reflection of total area captured, then the conclusion does not seem very interesting.

This is an important point. Figure 3c shows damage associated with territorial change across populated areas, so the unit of observation is the populated area, not territory. We compared building counts in populated areas gained by Russian forces and retaken by Ukrainian forces across our period of analysis and found no systematic differences in size. However, we agree that this was not clear in the previous version, and we have addressed it directly by using normalized counts in the revised figure (see next comment).

Related, isn't it likely that territory recaptured by Ukraine had already undergone considerable destruction during the prior fighting, so there was less opportunity for more destruction? Furthermore, even if more buildings were destroyed when Ukraine re-took the area, the SAR CCD method would not detect it if it had already marked that pixel area as 'damaged'.

This is an important point and an omission in the previous version. The revised manuscript uses normalized counts of newly damaged buildings per 1,000 intact buildings across populated areas. The substantive findings remain unchanged. Please also note that some of the areas that experienced the heaviest damage during Russian offensives (where the problem of prior destruction would be most pertinent) were contested for multiple months and therefore do not meet our criteria of a stable first transition. These areas are excluded from the analysis.

In Appendix D of the supplementary information, we added a two-step statistical analysis comparing territorial changes associated with Russian versus Ukrainian gains, using the normalized counts. The analysis shows that, in the months around first stable transitions, Russian territorial gains are more often associated with building damage than Ukrainian gains, and that the number of newly damaged buildings per 1,000 intact buildings is substantially and significantly higher for Russian gains.

We have updated Figures 3c, D1, and D2 to show the new version with the normalized counts. The caption of Figure 3c and the Methods section (Section 5.1) now explain how we normalized and accounted for prior building damage. The main text clarifies that the differences we find are for our period of analysis, and Appendix D discusses the statistical findings and how we interpret them.

Also, the error bars for Fig. 3c are not defined.

We have added a clarification in the caption of Figure 3c that these are box plots and added Q1, m, and Q3 in the box plots.

For Fig 5c & 5d, I wasn't able to easily figure out where the Estimated damage numbers came from. I assume it is described in section 5, but can you please make this clearer?

We have now included a brief summary of the estimation method (adjusting for reported true positive rates) in the caption and refer to Section 5.1.

Though I did not run all of the provided code, I reviewed the Python and R code and did not find anything particularly problematic.

Thank you very much for checking the code.

Minor:

Fig. 1 -- some of the arrows/lines need to be cleaned up.

Thank you for noticing, we have addressed this and cleaned up Figure 1.

Appendix A is never cited in the text.

Thank you for noticing. We mistakenly cited Appendix B instead of Appendix A in the previous version of the manuscript (Section 2.2, second paragraph). We have now addressed this.

It would be helpful to add labels to each of the Fig 4 & 5 panels to denote the different townships. Also in Figs D6 & D7.

We agree that it is easier for readers to read the figures with the names of the townships and have made these adjustments to Figures 4, 5, D3, D6 and D7.

Remarks on code availability:

I did not run the code due to time constraints. The readme.md file appears to be sufficiently detailed for this to be a successful endeavor, but different machine configurations can always pose a challenge. Given the number of co-authors on the manuscript, I suggest asking each of

them to run the code on their own machines since they have more of an incentive for publication than I do.

Thank you for checking the README file and for the suggestion. We have successfully run the script on multiple computers with different configurations, primarily on macOS. We also reproduced all figures and results on a Windows machine, which required some additional configurations of system paths and dependencies. To simplify reproduction, we provide a shell script that runs the full analysis end-to-end. The script installs current R package versions from CRAN rather than relying on pinned versions, which avoids the system-dependency, compiler, and R-version incompatibilities that pinned installations can encounter. The R analysis scripts are deterministic, so current package versions reproduce the figures; in rare cases, changes to package defaults could cause minor differences. For users who want to reproduce the exact package versions, we also provide pinned renv lockfiles. The shell scripts are written for Bash, with installation instructions provided for macOS. The README documents all of this.

Revision Memo

Dear editors, dear reviewers:

Thank you for the in-principle offer to publish our manuscript as an Analysis in *Nature*. With armed conflict on the rise and technology reshaping how wars are waged, this is a fitting moment for an article that sets out a vision and a scalable path for using satellite-derived data to better understand and respond to armed conflict.

We respond to the editorial requests and the remaining referee comments below. Thank you for your engagement and your constructive comments throughout the review process.

Editor comments:

Dear Dr Sticher,

Your manuscript, "Advancing conflict research and response through satellite-derived data", has now been seen by our referees, and in the light of their advice I am delighted to say that we can in principle offer to publish it. First, however, we would like you to revise your paper to address the points made by the referees, and to make some editorial changes to your paper so that it is as brief as possible and complies with our Guide to Authors. No peer reviewed data should be removed altogether when making these changes.

We have prepared our material in line with these instructions, as set out below.

1-Please address the remaining referee comments.

We address all remaining comments point by point below.

2-Statistics: Please add direct comparisons to your quantitative descriptive results, i.e. where you say the amounts and how they are different, please add statistics to show whether these differences are statistically significant or not. When you do this, please be sure that all statistical details are reported in full (name of the test, test statistic, sample size/degrees of freedom, p value, effect size, when appropriate, etc., in the Results, etc.). Please also be sure to check the test assumptions and to correct for multiple comparisons, when necessary. If you do get non-significant results, please do not interpret them unless you add statistical support for the null (e.g. equivalence test or Bayes factors) and throughout the manuscript. If you have this information in a figure legend, please add it in the Main text and vice versa.

We now report the key statistical results in the main text and the full statistical detail in Extended Data Tables 1–3, and describe the methodology in the Methods section.

3-Please provide the manuscript in .docx format. Currently it is in PDF format.

We now submit the main manuscript in .docx format.

4-Please create a separate reference list for any methods references, making sure that the numbering continues from the main text references.

We now provide a separate reference list for the methods references, numbered continuously from the main text references.

5-Please remove the main figures from the article file and re-supply them individually in an acceptable format such as EPS, AI, PS, PDF, PPT, PSD or XLS (for graphs) with editable vector files.

We have removed the main figures from the article file and supply them individually in editable .ai and .eps formats, following the *Nature* artwork guidelines.

6-We have noticed that not all corresponding authors of the manuscript are ORCID linked. As part of our efforts to improve transparency in authorship, we now request that all authors identified as 'corresponding author' create and link their Open Researcher and Contributor Identifier (ORCID) with their account on the Manuscript Tracking System (MTS) prior to acceptance. For more information please visit http://www.springernature.com/orcid. If you have an alternative account which is ORCID linked, please contact calla.smith-triglia@us.nature.com

All corresponding authors are now ORCID-linked on the Nature MTS account.

7-Please provide a supplementary information guide as a separate word document.

We provide a supplementary information guide as separate word document.

8-There are potential third party rights issues in the figures. Please check the sources of all illustrations and clarify whether permissions are needed to adapt or reproduce them. Please make sure to include the relevant details in third party rights table when you resubmit (more information below). If Biorender or a similar software has been used, please also ensure to provide relevant licenses. In particular please check figure/s: Figure 1a-b, 2a-h, 3b, 6a-b, Fig. D4 a-e, D5a-e.

We have checked the rights for all flagged figures and provide the details in the Third Party Rights Table.

9-Please make sure to provide a third party rights table (more information below) when you resubmit. If Biorender or similar software has been used, please also ensure to provide relevant licenses.

We include the Third Party Rights Table in our submission.

10-Please provide a data availability statement and a code availability statement, in the main text of the manuscript. These should be as detailed as possible (links to access the test datasets that can be shared, syntax files, etc.).

We now include separate data and code availability statements in the main text. The readme file in the replication package gives instructions for replicating the statistical results and all figures.

11-The funding information has been merged with the acknowledgment section; please provide it as a separate section.

We now provide the funding information in a separate section.

12-

13-Please re-supply the Extended data figures individually in EPS, JPEG or TIF format.

We supply all Extended Data figures individually in EPS format.

14-The funding information has been merged with the acknowledgment section; please provide it as a separate section.

As above, the funding information now appears in a separate section.

15-Please ensure that the text size in all figures is at least 5 pt Arial.

Text size in all figures is now at least 5 pt Arial.

Referee #1

I read the new version and the replies with great interest. All my comments were addressed.

Minor points:

I am sorry to raise this so late — I should have spotted it earlier — but I don't find Figure 5 particularly intuitive. I think the authors mean to describe the relative shapes of the fatality and destruction series here. Perhaps this could be conveyed better with per-period numbers rather than cumulative ones? Relatedly, I find it confusing that the top panel is in absolute terms while the bottom panel is relative to the total. At the very least, I think it would help to have clearer labels (e.g. "fatalities" instead of "best") and perhaps a visual cue flagging where the authors want the reader to focus.

We agree that Figure 5 was hard to read, given the differing axes in the top and bottom panels, the small font, and the labels. We made three changes. First, we now report fatalities as a percentage of the pre-war population, parallel to how we report building damage (damaged buildings as a percentage of total buildings). Second, we relabeled the fatality estimates as 'Fatalities (high spatial precision)' and 'Fatalities (lower spatial precision)' and clarify in the caption which data we use. Third, we enlarged the font to make the labels and axis labels more readable. We considered switching to per-period numbers but retained the cumulative series, because the figure's main message is the overall scope of fatalities and destruction across the two cities. The cumulative form makes the end-state magnitude directly legible, while the slope still conveys the timing of accumulation that we mention in the discussion of encirclement and evacuation. We revised the caption title to reflect this focus on scope, and applied the same changes to Extended Data Fig. 8, which compares best and high fatality estimates.

Data sources in Figures 3, D1 and D2 are listed as [9, 31], but as far as I can tell the territorial control (and tessellation) data come from VIINA 2.0, which is cited as a separate reference in the Methods 69? Perhaps the 31 reference presents the data as well so this is just minor point.

We have corrected this inconsistency and now reference the data directly in the figure captions (now Fig. 3, Extended Data Fig. 3, and Extended Data Fig. 4).

I had some trouble reconciling the sample used in the Appendix D tests with the Methods. The tables are based on 95 Russian and 148 Ukrainian cells, whereas the Methods report 200 and 967 first stable transitions for the frontline period. I assume the difference is a restriction to populated areas, but the intermediate steps are not spelled out. A short sample-construction summary would help.

Thank you for noticing this omission. We restricted the analyses to populated areas, removing geometries with no buildings in our building layer. We have added two sentences to the Methods section explaining this restriction and reporting the number of populated areas it leaves for each actor and analysis period.

Referee #5

Thanks to the authors for their careful attention to my prior suggestions. I like the changes to Figure 1.

The only additional suggestion I have relates to terminology and engaging a bit with an existing literature. The first part of the 'Improvement' section (the Fig. 2 examples) seems to be a form of dasymetric mapping or areal interpolation. I suggest acknowledging this connection to prior work with a citation to Eicher & Brewer (2001) or Mennis (2003) and any other work (maybe Wright 1936) you find relevant.

Thank you for pointing us to this relevant literature. We agree that our spatial improvement approach relates to dasymetric mapping and now cite this body of work (footnote c and references 54, 55).

Remarks on code availability:

I did not look at the code for this round of review. I did not see the code as part of the submitted documents for review, and the zenodo link appeared to have restricted access. Even if I had access, I would not have been able to spend much time reviewing it.

We have created an updated replication package with the final figure numbering and a new file documenting the source, citation, and license of all third-party data. The link to this repository appears in the main manuscript and in the Code and Software Submission Checklist. The repository is currently under embargo; we will set the embargo date once the article is accepted and we have a publication date. In the meantime, access is restricted, and as before we will share the access link with the editorial office so reviewers can access all scripts and data.

The article has improved dramatically. I congratulate the authors.

However, I am still concerned about the fact that the conceptual framework is somewhat superficial. It has no formal elements and does not talk about the data generating process in detail. Reading the guidelines by the editor I could see that no data analysis is allowed in the format here. This closes a big potential avenue to illustrate the points of the paper and give them more depth (see below). So, in what follows I will try to bounce some ideas. I know this is not great refereeing in the sense that I am asking a lot but please take the following as inspiration and an attempt for shifting the debate into more concrete territory to clarify my point from the first review.

Perhaps my previous report was too vague but I think we are missing a map and this is what I would ask of a perspective article. Some ideas, please don't take them literally:

1) What are *all* the measurable dimensions of conflict we could care about and how could they relate to the conflict process? Then after the where are satellites adding and what should be the priority? Some ideas: tents/temporary housing, refugee trails, cars, transport trucks, road destruction, building destruction, vegetation destruction, fires, rubble, night light, market activity, military equipment, weapon craters.
2) Combine this perhaps with a discussion on dimensions such as: importance for policy, importance for research, measurability with satellites, comparative advantage over text to derive a score of what could be a priority going forward.
3) I paste the Global Registry of Violent Deaths (GReVD) conceptualization of how different violence measurement types could interact to illustrate how detailed others in this sector have approached similar topics. I know this is not a perfect fit as the goal here was something else. But perhaps this inspires the authors to more.

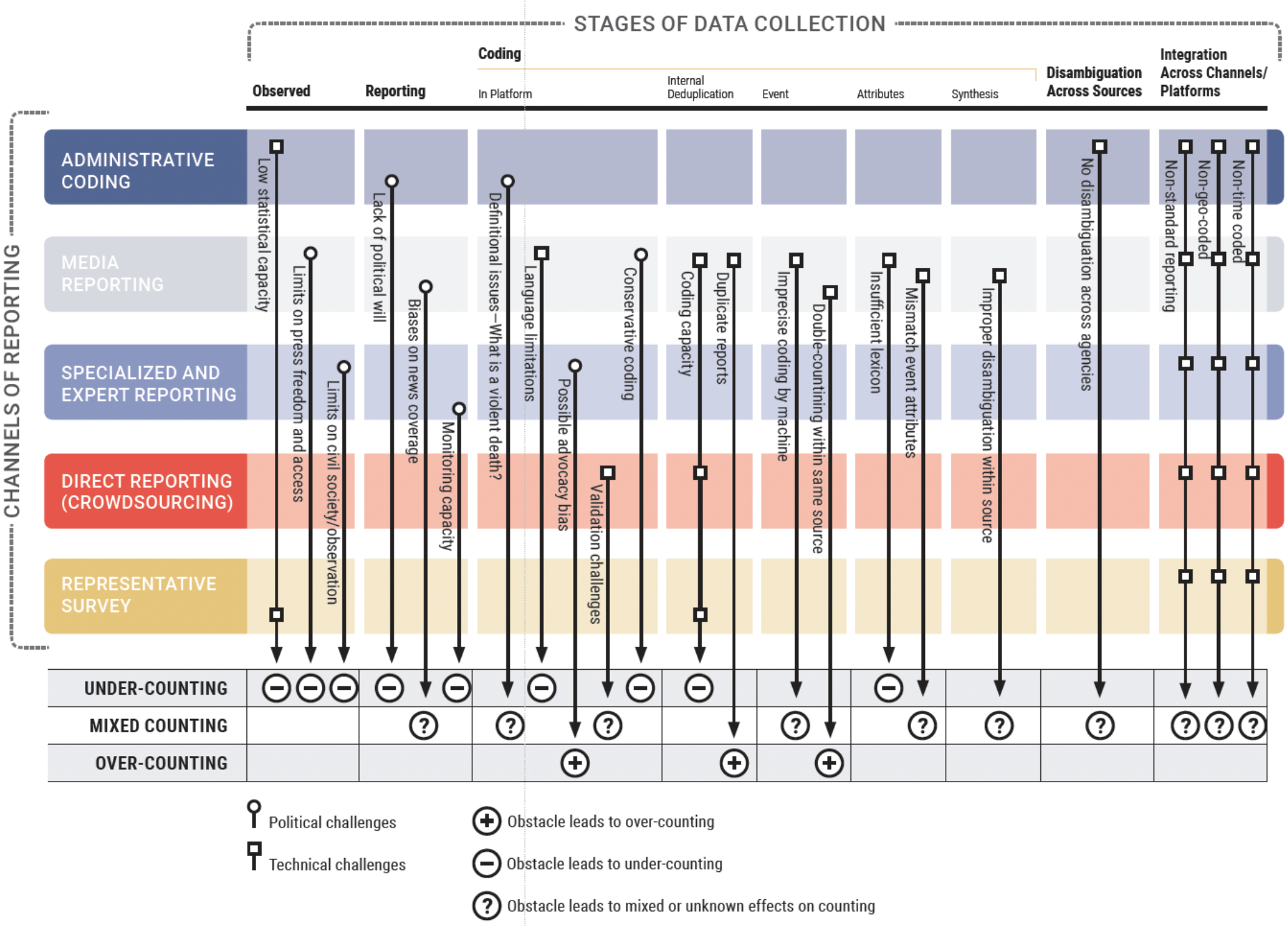

I was really surprised there is no data now. I understand this is the steer of the editor. But if this data and analysis does not exist then at the heart of the article and the "ways forward" it should be at least mentioned that we do not have panel data that would allow us understand how satellite features like destruction data could complement other data. I was under the impression that someone must have generated destruction panel data from a war zone like Gaza or Ukraine with all the many ongoing projects in this area. The fact that a large team like this is not able to muster a single a dataset means that there is a very clear identifiable gap that should be mentioned. Putting it starkly: **we currently have no destruction data that could be merged with the news event data to make it possible to analyze how destruction data can add to the analysis of conflict**. In section 6 the team goes into ethical issues and datasets being specific to one context. But the elephant in the room is clearly that no data can be deployed to analyze destruction over time and locations. So, the way forward is to generate data like this. I personally find this *very* motivating.

```
#!/usr/bin/env bash
# run_main.sh - Run all figure-generation scripts.
#
# Installs current R package versions from CRAN into the standard user
# library and runs every R script with --vanilla, which skips each
# subfolder's .Rprofile and keeps renv dormant. This avoids system-
# dependency and compiler incompatibilities that pinned installs can
create.
#
# A pinned renv lockfile is included in 01_myanmar/r_analysis and
# 03_ukraine_enrichment/r_analysis for users who want to reproduce the
# exact package versions. See the README for details.

set -euo pipefail

MAIN_DIR="$(cd "$(dirname "${BASH_SOURCE[0]}")" && pwd)"

# --- Find Rscript ---
if command -v Rscript >/dev/null 2>&1; then
  RSCRIPT="Rscript"
else
  # Check standard Program Files first (only if they exist)
  SEARCH_PATHS=()
  [[ -d "/c/Program Files/R" ]] && SEARCH_PATHS+=("/c/Program Files/R")
  [[ -d "/c/Program Files (x86)/R" ]] && SEARCH_PATHS+=("/c/Program Files
(x86)/R")

  RSCRIPT=""
  if [[ ${#SEARCH_PATHS[@]} -gt 0 ]]; then
    RSCRIPT="$(find "${SEARCH_PATHS[@]}" -type f -name "Rscript.exe"
2>/dev/null | sort -V | tail -n 1)"
  fi

  # Fall back to user home directory with limited depth if not found in
Program Files
  if [[ -z "${RSCRIPT}" ]]; then
    if [[ -d "${HOME}" ]]; then
      RSCRIPT="$(find "${HOME}" -maxdepth 5 -type f -name "Rscript.exe"
2>/dev/null | sort -V | tail -n 1)"
    fi
  fi
fi

if [[ -z "${RSCRIPT}" ]]; then
  echo "ERROR: Rscript not found. Please install R from https://cran.r-
project.org/"
  exit 1
fi

# --vanilla makes R skip startup files, including each subfolder's
# .Rprofile, which keeps renv dormant. The pinned renv lockfiles are
# therefore not used by this script; see the README for the renv path.

R_FLAGS="--vanilla"
export RSCRIPT R_FLAGS
```

```
# -------------------------------------------------------------

# ---- Install R packages once ----
# Both R subfolders use the same packages and the standard user library,
# so a single installation covers Step 1 and Step 3.

echo ">>> Installing R packages"
echo ""

"$RSCRIPT" $R_FLAGS "$MAIN_DIR/install_packages.R"

echo ""

# ---- Step 1: Myanmar Improvement and Enrichment figures ----

echo ">>> Running 01_myanmar/r_analysis/myanmar_analyses.R"
echo ""

cd "$MAIN_DIR/01_myanmar/r_analysis"
"$RSCRIPT" $R_FLAGS myanmar_analyses.R

echo ""
echo ">>> Step 1 complete."
echo ""

# ---- Step 2: Ukraine Improvement figures ----
echo ">>> Running 02_ukraine_improvement/run_figures2.sh"
echo ""

cd "$MAIN_DIR/02_ukraine_improvement"
bash run_figures2.sh

echo ""
echo ">>> Step 2 complete."
echo ""

# ---- Step 3: Ukraine Enrichment figures ----

echo ">>> Running 03_ukraine_enrichment/run_figures3.sh"
echo ""

cd "$MAIN_DIR/03_ukraine_enrichment"
bash run_figures3.sh

echo ""
echo ">>> Step 3 complete."
echo ""

echo "  All Done."
```
